# Machine learning combining multi-omics data and network algorithms identifies adrenocortical carcinoma prognostic biomarkers

**DOI:** 10.3389/fmolb.2023.1258902

**Published:** 2023-11-06

**Authors:** Roberto Martin-Hernandez, Sergio Espeso-Gil, Clara Domingo, Pablo Latorre, Sergi Hervas, Jose Ramon Hernandez Mora, Ekaterina Kotelnikova

**Affiliations:** Discovery and Translational Sciences (DTS), Clarivate Analytics, Barcelona, Spain

**Keywords:** ACC, multi-omics, machine learning, systems biology, survival analysis, prognostic biomarkers

## Abstract

**Background:** Rare endocrine cancers such as Adrenocortical Carcinoma (ACC) present a serious diagnostic and prognostication challenge. The knowledge about ACC pathogenesis is incomplete, and patients have limited therapeutic options. Identification of molecular drivers and effective biomarkers is required for timely diagnosis of the disease and stratify patients to offer the most beneficial treatments. In this study we demonstrate how machine learning methods integrating multi-omics data, in combination with system biology tools, can contribute to the identification of new prognostic biomarkers for ACC.

**Methods:** ACC gene expression and DNA methylation datasets were downloaded from the Xena Browser (GDC TCGA Adrenocortical Carcinoma cohort). A highly correlated multi-omics signature discriminating groups of samples was identified with the data integration analysis for biomarker discovery using latent components (DIABLO) method. Additional regulators of the identified signature were discovered using Clarivate CBDD (Computational Biology for Drug Discovery) network propagation and hidden nodes algorithms on a curated network of molecular interactions (MetaBase™). The discriminative power of the multi-omics signature and their regulators was delineated by training a random forest classifier using 55 samples, by employing a 10-fold cross validation with five iterations. The prognostic value of the identified biomarkers was further assessed on an external ACC dataset obtained from GEO (GSE49280) using the Kaplan-Meier estimator method. An optimal prognostic signature was finally derived using the stepwise Akaike Information Criterion (AIC) that allowed categorization of samples into high and low-risk groups.

**Results:** A multi-omics signature including genes, micro RNA's and methylation sites was generated. Systems biology tools identified additional genes regulating the features included in the multi-omics signature. RNA-seq, miRNA-seq and DNA methylation sets of features revealed a high power to classify patients from stages I-II and stages III-IV, outperforming previously identified prognostic biomarkers. Using an independent dataset, associations of the genes included in the signature with Overall Survival (OS) data demonstrated that patients with differential expression levels of 8 genes and 4 micro RNA's showed a statistically significant decrease in OS. We also found an independent prognostic signature for ACC with potential use in clinical practice, combining 9-gene/micro RNA features, that successfully predicted high-risk ACC cancer patients.

**Conclusion:** Machine learning and integrative analysis of multi-omics data, in combination with Clarivate CBDD systems biology tools, identified a set of biomarkers with high prognostic value for ACC disease. Multi-omics data is a promising resource for the identification of drivers and new prognostic biomarkers in rare diseases that could be used in clinical practice.

## Introduction

Adrenocortical carcinoma (ACC) is a rare and aggressive cancer that originates in the adrenal gland cortex. Among all adrenal tumors, adrenocortical carcinoma is one of the most prevalent cancers with one of the worst prognoses (Mansmann et al., 2004). Despite advancements in cancer research, very limited therapeutic options are available (Shariq and McKenzie, 2021). Although, it has been associated with other malignancies such as Li-Fraumeni or Beckwith-Wiedemann syndromes, the indication remains sporadic with an unknown cause. This underscores the critical need to identify robust prognostic biomarkers that can effectively stratify patients for personalized therapeutic approaches.

In recent years, several molecular pathways, such as Ghrelin and Wnt signaling, have been described in relation to ACC (Komarowska et al., 2018). Although some genes associated with the disease predisposition have been discovered, particularly IGF-2, is still unclear which factors contribute most significantly to the development of ACC (Libé and Chanson, 2007).

The limited curative treatments and life expectancy for rare diseases underline the existing caveats in trying to understand the multifactorial nature of these indications. To close this gap, combination of multiple omics has been demonstrated to be previously useful, offering comprehensive insights into the molecular landscape of a specific biological phenomenon (Subramanian et al., 2020). For example, it has been shown to be effective in early blood biomarker characterization for ovarian cancer detection (Xiao et al., 2022); as well as in tumor subtype classification (Pucher et al., 2019) and target discovery for successful centronuclear myopathy treatment (Djeddi et al., 2021). In the context of ACC, this approach has been recently shown to be useful for patient stratification (Guan et al., 2022).

By simultaneously analyzing various molecular layers, biomarkers derived from multi-omics analysis can provide a holistic view of the molecular modifications responsible for the disease. To tackle omics integration complexity, recent developments in machine learning and multivariate methods have helped ease the process (Wang et al., 2022). However, it is equally crucial to pinpoint the upstream regulators that drive these changes to gain a more complete understanding of the underlying mechanisms. Network algorithms, including Hidden Nodes (Dezso et al., 2009), Random Walk (Smedley et al., 2014), and Network Propagation (Vanunu et al., 2010), have emerged as valuable tools in this regard, as they allow the identification of pivotal genes that indicate prospective regulators of the observed alterations based on their topological significance. This additional layer of information may play a crucial role in unravelling the molecular alterations linked to diseases and may enhance prognostic accuracy.

To achieve effective patient stratification, it is essential to accurately categorize patients based on the aforementioned data modalities and inferred regulators. In the context of precision medicine, machine learning techniques have been widely employed for this purpose. Notably, among these techniques, the random forest algorithm efficiently and accurately addresses the inherent challenges of integrating diverse data sources (Assié et al., 2014).

Overall, the combination of multi-omics approaches, network algorithms, and machine learning methods offers a promising framework for enhancing our understanding of ACC and for improving patient stratification for personalized therapeutic strategies. In this study, we proposed a novel machine learning method based on Projection to Latent Structures (PLS) with sparse discriminant analysis, namely, DIABLO, integrating ACC RNA-seq, miRNA-seq and DNA methylation data for biomarker identification. To maximize target identification, we coupled it with Clarivate CBDD (Computational Biology for Drug Discovery) network algorithm pipelines using MetaBase™ molecular network, a high-quality and scientifically validated interactome with more than 300.000 interactions curated by Clarivate experts. Our results show the benefit of the usage of novel machine learning methods coupled with Clarivate bioinformatics workflows and network algorithms.

## Materials and methods

### Adrenocortical carcinoma data acquisition

GDC TCGA Adrenocortical Carcinoma (ACC) datasets (Zheng et al., 2016), and the corresponding phenotypic and clinical data, were downloaded from the UCSC Xena Browser (https://xenabrowser.net/datapages/) (Goldman et al., 2020). Three types of omics data were selected due to their complementarity and their suitability for the latent variable used approach: RNA-seq gene expression data normalized in Fragments Per Kilobase Million (FPKM) units, micro RNA expression data normalized in RPM units, and Illumina 450 K methylation microarray data as beta values. A total of 79 samples derived from different patients are shared among the three datasets.

### Multi-omics data preprocessing and integration analysis

Predictor variables with low variance across all the samples were filtered out from each dataset, selecting features with a median absolute deviation above the third quartile. Datasets from different types of omics were integrated using a multiblock Projection to Latent Structure—Discriminant Analysis (PLS-DA) approach. Specifically, the *block.splsda* function from Data Integration Analysis for Biomarker discovery using Latent variable approaches for Omics studies (DIABLO) framework (Singh et al., 2019), available within the mixOmics R library (v. 6.24.0), was used with default parameters. The relationship structure between inputted datasets was defined using a design matrix with value of 0.1, to prioritize the predictive ability of the model. Correlations of the latent variables with available clinical data and their significance was assessed using the *eigencorplot* function from the PCAtools R library (v. 2.12.0) with default parameters.

### Systems biology tools

MetaBase™ (v.4.8.0) a data repository of manually curated molecular interactions and signaling pathways from Clarivate Analytics, was used as the source of biological knowledge in this study. The database includes over 1.500 regulatory and diseases-specific pathway maps, and a network with more than 3.3 million of molecular interactions. Network algorithms were leveraged from the industry-leading systems biology consortium CBDD analytical library (v.17.2.0), developed by Clarivate Analytics. For the network propagation and hidden nodes analyses, network nodes with scores below 0.1 and 20 respectively, were dropped from the analysis.

### Machine learning classifier

A Random Forest (RF) model with repeated cross-validation (5 times) and 500 trees was trained based on 70% of the samples, using combinations of different features. The number of features randomly selected for each tree (mtry) ranged within 3, 5, 7. Selected division criteria was by “splitrule,” and the minimum node’s size for division ranged within 2, 4, 6. Computations were performed using the caret package (v.6.0–94) in R. Specifically, the “ranger” method and “AUC” metric were used in the training step.

### Survival analysis and construction of a prognostic model

Univariate association of the features with overall survival (OS) was estimated by log-rank test and the Kaplan-Meier method. Survival *coxph* function in Survival R package (version 3.5.5) was used to perform univariate and multivariate Cox Proportional-Harzards (CoxPH) regression analyses. Survival curves were drawn using survminer R package (version 0.4.9). Patients with low expression were used as the reference group for all the analyzed features. Weiss score and age (as continuous variable) were included in the multivariate Cox PH model to assess simultaneously the effect of the factors on survival time.

Then, we constructed an optimal prognostic signature associated with OS through sequential addition or elimination of features based on their performance using the Akaike Information Criterion (AIC) as proposed by Wagenmakers, Eric-Jan, and Simon Farrell (Wagenmakers and Farrell, 2004). Specifically, the *step* function with backwards selection from the stats R package was used, in which variable terms were evaluated for dropping at each step. Finally, the optimal prognostic model obtained was defined as risk score using the following equation:

$$OptMultiSig\;risk\;score=\sum_{i=1}^{n}coef\;OptMultiSig_{i}\times \mathrm{EXP}\;OptMultiSig_{i}$$

where OptMultiSig risk score is the prognostic risk score of ACC patients and coef OptMultiSig_i_ are the i^th^optimal multi-omics feature’s regression coefficient obtained from the step analysis. Based on the median risk score, ACC patients were divided into high- and low-risk groups. Next, we assessed the difference in survival between the two groups by using Kaplan-Meier method. We considered *p*-value <0.05 as statistically significant.

### Cortellis drug discovery intelligence biomarkers and drug targets

CDDI (https://www.cortellis.com/drugdiscovery) is a knowledgebase focused on pharma and drug development, developed and maintained by Clarivate Analytics. Biomarker data used in this study was downloaded from CDDI (June 2023). The database integrates biological, chemical, and pharmacological data on more than 620,000 molecules with demonstrated biological activity, and over 440,000 patent family records. Main information about biomarkers include data about biomarker type (proteomic, biochemical, etc.), development stage, context of their usage (diagnosis, risk detection, etc.), number of related drugs, related literature citations and patents, proof-of-mechanism, proof-of-concept, treatment/safety monitoring, and outcome measurement. Drug targets include annotation about structural data from the Protein Data Bank (PDB) and comprehensive data regarding the development phase (clinical phases, launched or withdrawn) of the associated drugs for different therapeutic areas or indications.

## Results

### Multi-omics data integration and biomarker identification

Gene expression and methylation data were used for the multi-omics integration. After pre-processing, 15.120 features were retained from RNA-Seq data, 553 from miRNA-Seq data, and 98.588 from DNA methylation data. Then, we removed samples corresponding to normal tissue and samples with missing data at the level of tumor stage diagnosis, keeping for the analysis 77 samples from 77 different patients. Information about the included samples and related metadata is available in Supplementary Table S1.

We used an N-integration approach with supervised learning within the DIABLO framework to identify highly correlated multi-omics features discriminating ACC at different stages. These features, which capture the maximum shared variation within each data type, are included in the latent components extracted by the framework. To control the imbalance of samples belonging to different disease stages, the initial four stages were grouped into two main stages (stages I-II and stages III-IV). Integration was performed using sparse Partial Least Squares Discriminant Analysis (sPLS-DA), which enables the selection of the most predictive or discriminative features in the data that assist in the classification of samples. We started generating sPLS-DA models with up to 5 components to estimate the classification error rate between stages with respect to the number of selected variables. This strategy allowed us to tune the number of components and variables from each dataset to be retained in the final model. A 3 × 3 design matrix was used to determine whether the datasets should be connected. The value of the design matrix was set at 0.1 as a default value to prioritize the discriminative ability of the model (Tenenhaus and Tenenhaus, 2014). A total of five components were left for use in the final model, including 46 features from both RNA-Seq and miRNA-Seq data, and 65 from DNA methylation data (Table 1).

**TABLE 1:** Optimal number of features to retain in the final model, from each omic dataset and for five components.

	RNA seq	miRNA seq	DNA methylation
**Component 1**	10	12	25
**Component 2**	7	5	5
**Component 3**	9	6	10
**Component 4**	5	14	5
**Component 5**	15	9	20

**FIGURE 1:**
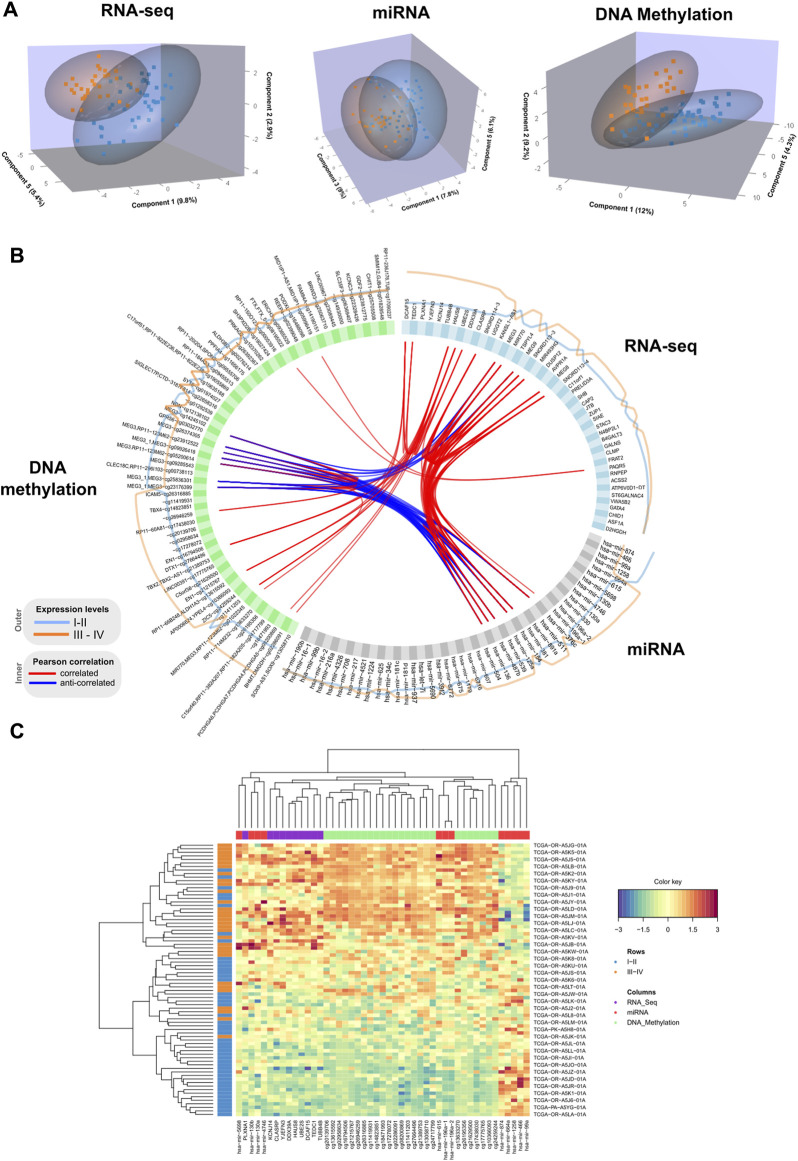
**(A)** 3D samples plots for RNA-Seq, micro RNA and DNA methylation data. For each OMICs dataset, samples are projected into the space spanned by the three components explaining most of the variance. Disease stage is represented with different colors. **(B)**. Circos plot representing correlations among features from different data types. Only variables with absolute correlations higher than 0.75 are shown. Outer lines represent the expression levels for each variable from the two different stage groups. **(C)**. Clustered heatmap using variables included in the component 1 from the multiblock sPLS-DA model. Samples are represented in rows, and features from different data type are represented in columns. Euclidean distance and Complete linkage methods are used for clustering. The standardized abundance level is shown by the color key.

We performed a final N-integrative supervised analysis using the defined features. At the single omics dataset level, the identified features explained a maximum of 30% of the total variance among the five components (Table 2). DNA methylation was the omics dataset that explained the highest amount of variance, followed by the miRNA-Seq and RNA-Seq datasets. Details regarding the features included in each component from the three omics datasets, including the obtained loadings, can be found in Tables 3–5. Visualization of the samples projected onto the three components reporting a higher explained variance allowed us to successfully account for the separation observed between the defined stages. (Figure 1A). The features included in the final model exhibited a high level of correlation (Figure 1B). Indeed, higher positive correlation levels were observed between RNA-Seq and micro RNA features, whereas DNA methylation features showed higher levels of negative correlation with both RNA-Seq and micro RNA features. Furthermore, we visualized the cluster structure resulting from the multi-omics features found in the first component of the sPLS-DA model, which included the larger number of features amongst the 5 defined components (47) (Figure 1C). The heatmap shows that samples (rows) from the same disease stage category tend to cluster together. Additionally, micro RNA and methylation blocks showed opposite abundance levels based on the disease stages, with the set of methylation sites being more abundant in III-IV stages, whereas the set of micro RNA’s was more abundant in I-II stages.

**TABLE 2:** Percentage of variance explained in the final model from each omic dataset and for five components.

	RNA seq	miRNA seq	DNA methylation
**Component 1**	9.8	7.8	12
**Component 2**	2.9	2.4	9.2
**Component 3**	1.9	9	1.5
**Component 4**	2.4	1.7	2.6
**Component 5**	5.4	6.1	4.3
**Total variance explained**	**22.4**	**27**	**29.6**

Bold values are the sum of percentages in the upper cells (% total variance explained).

**TABLE 3:** Correlated features from RNA-Seq dataset obtained after multi-omics data integration. Corresponding loadings are reported with the feature name.

Component 1	Component 2	Component 3	Component 4	Component 5
TEDCA (0.61)	ASF1A (0.49)	SNORD114-3 (−0.58)	PAQR5 (0.98)	ST6GALNAC4 (0.41)
DCAF15 (0.58)	ZUP1 (0.24)	MEG3 (−0.58)	ACSS2 (0.12)	CHID1 (0.33)
UBE2S (0.3)	GATA4 (0.23)	MEG9 (−0.39)	CAP2 (0.04)	GALNS (0.28)
HAUS8 (0.27)	TSPYL4 (0.07)	MIR770 (−0.37)	STAC3 (−0.16)	B4GALT3 (0.24)
PLXNA1 (0.21)	SIAE (−0.78)	MEG8 (−0.18)	KANSL1-AS1 (−0.02)	N4BP2L1 (0.22)
YJEFN3 (0.21)	ATP6V0D1-DT (−0.15)	SNORD113-3 (−0.08)		SHB (0.22)
KCNJ14 (0.17)	UGGT2 (−0.04)	C11orf1 (−0.03)		D2HGDH (0.21)
DDX39A (0.13)		SNORD113-4 (−0.03)		DUSP12 (0.16)
CLASRP (0.02)		MIR493HG (−0.03)		FRAT2 (0.13)
TUBB4B (0.01)				CLMP (0.12)
				AVPR1A (0.09)
				RNPEP (0.09)
				JTB (0.09)
				PRELID3A (0.01)
				VWA5B2 (−0.6)

**TABLE 4:** Correlated features from miRNA-Seq dataset and corresponding loadings obtained after multi-omics data integration. Corresponding loadings are reported with the feature name.

Component 1	Component 2	Component 3	Component 4	Component 5
hsa-mir-615 (0.59)	hsa-mir-1179 (0.38)	hsa-mir-539 (−0.7)	hsa-mir-675 (0.57)	hsa-mir-511 (0.1)
hsa-mir-130b (0.25)	hsa-mir-891a (−0.66)	hsa-mir-136 (−0.48)	hsa-mir-330 (0.34)	hsa-mir-937 (0.05)
hsa-mir-196a-2 (0.22)	hsa-mir-708 (−0.62)	hsa-mir-154 (−0.38)	hsa-mir-216a (0.18)	hsa-mir-190b (0.04)
hsa-mir-5698 (0.13)	hsa-mir-504 (−0.15)	hsa-mir-487b (−0.34)	hsa-mir-607 (0.16)	hsa-mir-1224 (−0.9)
hsa-mir-4746 (0.12)	hsa-mir-217 (−0.09)	hsa-mir-376c (−0.14)	hsa-mir-6716 (0.11)	hsa-mir-125a (−0.34)
hsa-mir-196a-1 (0.07)		hsa-mir-381 (−0.003)	hsa-mir-5690 (0.06)	hsa-mir-3912 (−0.18)
hsa-mir-130a (0.05)			hsa-mir-6772 (0.004)	hsa-mir-181c (−0.18)
hsa-mir-874 (−0.44)			hsa-mir-4326 (−0.48)	hsa-mir-181d (−0.14)
hsa-mir-99a (−0.36)			hsa-mir-625 (−0.38)	hsa-mir-99b (−0.09)
hsa-mir-1258 (−0.35)			hsa-mir-4521 (−0.3)	
hsa-mir-466 (−0.25)			hsa-mir-16–1 (−0.1)	
hsa-mir-664a (−0.03)			hsa-let-7i (−0.09)	
			hsa-mir-34c (−0.06)	
			hsa-mir-16–2 (−0.04)	

**TABLE 5:** Correlated features from DNA-Methylation dataset obtained after multi-omics data integration. Corresponding loadings are reported with the feature name.

Component 1	Component 2	Component 3	Component 4	Component 5
SOX9-AS1,SOX9-cg13058710 (0.53)	CLEC18C,RP11-296I103-cg00738113 (0.75)	MEG3,RP11-123M62-cg05200614 (0.56)	FTX,FTX_5-cg08195522 (0.58)	CHIT1-cg25705508 (−0.49)
BHMT,DMGDH-cg02286091 (0.42)	ALDH3B2-cg00276214 (0.44)	MEG3,RP11-123M62-cg23912522 (0.44)	C17orf51,RP11-822E236,RP11-822E238-cg19055869 (0.38)	LINC00967-cg23089445 (−0.43)
cg08200869 (0.41)	cg01292539 (0.4)	MEG3-cg09285543 (0.4)	MID1IP1-AS1,MID1IP1-cg05996419 (0.34)	SYT6-cg01974027 (−0.36)
cg11411203 (0.3)	cg26392367 (0.3)	MEG3-cg26374305 (0.37)	BRWD3-cg25063710 (0.25)	GPR56-cg03032770 (−0.33)
cg18471993 (0.3)	cg10635188 (0.09)	MEG3_1,MEG3-cg23176399 (0.32)	RP11-184A23-cg09455513 (−0.59)	FAM84A-cg14190151 (−0.32)
cg26195356 (0.26)		MEG3-cg14245102 (0.18)		PRKAG2-cg10370262 (−0.24)
cg24717799 (0.17))		MIR770,MEG3,RP11-123M62-cg01022345 (0.17)		RP11-236J176,TUB-cg17090237 (−0.23)
DTX1-cg27664496 (0.14)		MEG3_1,MEG3-cg25836301 (0.14)		RP11-20I204,SPON2-cg09555706 (−0.17)
LINC00391-cg17775765 (0.13)		MEG3_1,MEG3-cg09926418 (0.06)		RP11-150O123-cg00053916 (−0.15)
RP11-66B248,ALDH1A3-cg13615592 (0.1)		NDN-cg12138102 (−0.15)		SIGLEC17P,CTD-3187F814-cg22658316 (−0.13)
cg17278072 (0.1)				SH3PXD2B-cg19027424 (−0.11)
RP11-714M232-cg13633270 (0.09)				SLC35F3-cg06369407 (−0.1)
C5orf38-cg21629500 (0.09)				SMIM12,GJB4-cg01828548 (−0.09)
RP11-60A81-cg17438030 (0.08)				KCNC3-cg22328426 (−0.09)
cg20139706 (0.06)				GDF2-cg23812775 (−0.07)
ZIC5-cg24259244 (0.05)				REEP4-cg02399048 (−0.06)
EN1-cg16794506 (0.05)				PPFIA4-cg11656175 (−0.04)
cg02958634 (0.05)				ERICH3-cg09365529 (−0.03)
TBX2,TBX2-AS1-cg21389753 (0.04)				PODXL-cg16488098 (−0.01)
EN1-cg21215767 (0.04)				cg14930000 (−0.01)
AP0006624,YPEL4-cg10366093 (0.03)				
ICAM5-cg26316885 (0.02)				
cg26946259 (0.02)				
cg11419931 (0.02)				
TBX4-cg14823851 (0.01)				

Additionally, we inspected the correlations of the identified components from the three OMICs datatypes with available clinical data from the TCGA ACC dataset (Supplementary Figure S1). Statistically significant correlations were identified between the TNM classification system parameters and latent components. Specifically, lymph node stage (N) showed a significant positive correlation with component 2 (0.24), and tumor size (T) was negatively correlated with component 3 (−0.23). Among the obtained correlations, higher correlations ( ± 0.2) were observed between the Weiss score and components 1, 3 and 4, whereas Age showed a positive correlation with component 5 (0.2).

### Topological regulation analysis

We employed two complementary network algorithms to find genes associated with the newly identified multi-omics signature at a topological level. As most of the interactions included in MetaBase™ network are proteins, only the genes from the identified multi-omics signature were used as starting nodes. First, we used an algorithm based on Hidden Nodes method (HN) (Dezso et al., 2009), which prioritized nodes providing high connectivity between the seed nodes using a directed network. The statistical significance of the overconnected nodes was assessed using a hypergeometric test, and the *p*-values were adjusted considering all internal nodes. The scores for each node are the obtained adjusted *p*-values on a -log10 scale. It is a local method able to identify upstream regulators or downstream effectors of the input features. As a complementary approach, a network propagation (NP) algorithm (Vanunu et al., 2010) was employed, which is a global method making use of the whole network topology to find nodes highly connected to the input nodes. The scoring of nodes is done by simulating an iterative process where flow is pumped from the start nodes to their network neighbors. The identified features (Supplementary Table S2) include important regulators of gene expression cascades such as transcription factors, some of them from the same family (KLF7, KLF16), protein kinases, membrane receptors and other protein binding molecules.

**FIGURE 2:**
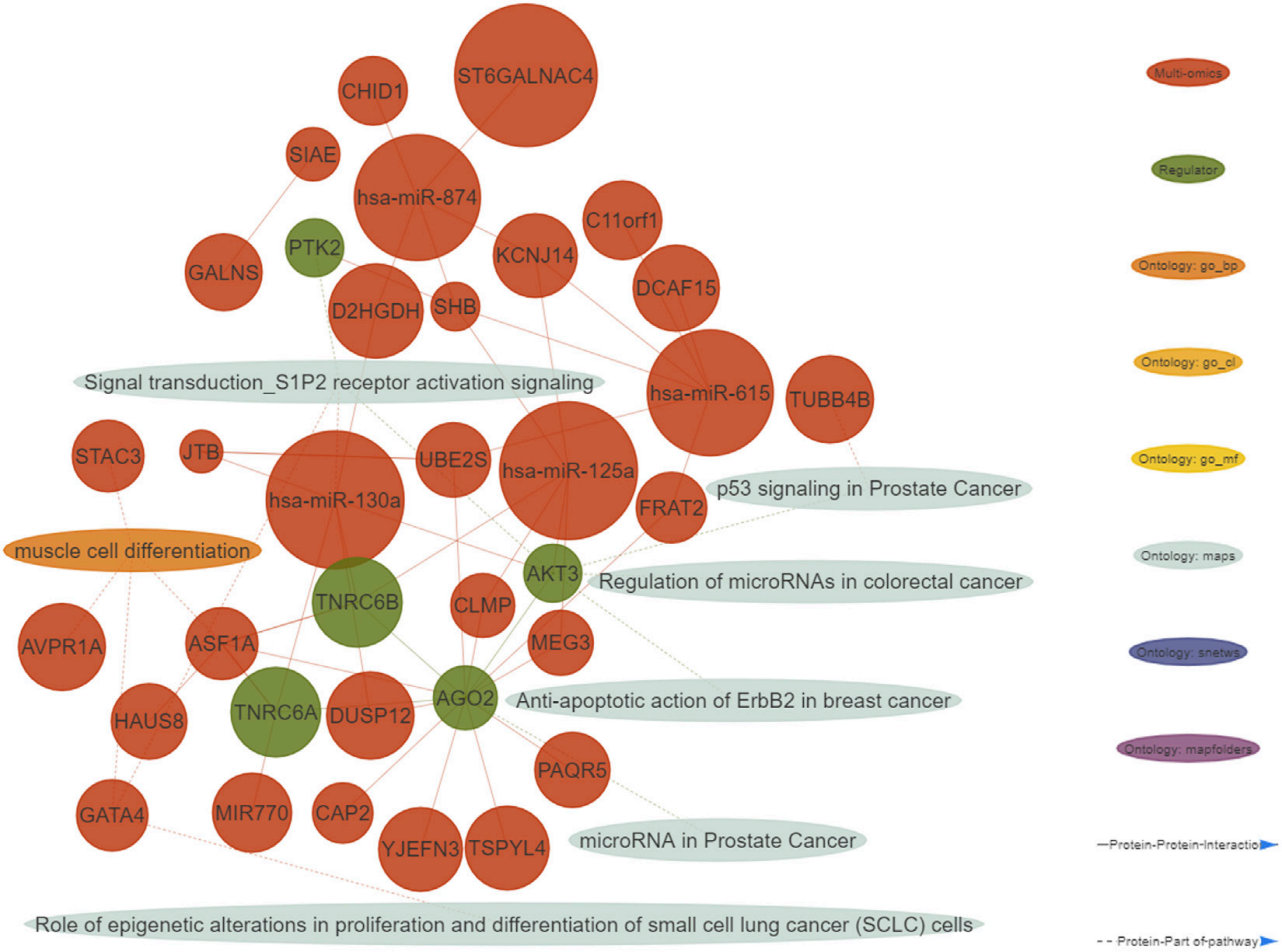
Integrative network consisting of genes and micro RNA’s identified in the multi-omics signature, their topological regulators and ontologies (nodes), and their relationships (edges). The nodes of the network were restricted to all the genes from the multi-omics signature and their regulators showing at least one interaction. Only micro RNA’s with at least five interactions are shown. Important ontologies in the network are enriched for multi-omics biomarkers (*p*-value≤0.01) and include at least 1 regulator. Regarding the edges of the network, two types of connections are considered: 1) Protein-Protein interactions among multi-omics biomarkers and their topological regulators, 2) Protein-Part of pathway, in which multi-omics biomarkers and their topological regulators belong or not to the aforementioned ontologies.

A functional enrichment of the identified multi-omics biomarkers was performed. We defined important enriched ontologies as the ones significantly enriched in the set of multi-omics biomarkers (*p*-value≤0.01) and including at least one of the identified topological regulators. The most enriched important ontologies belong to MetaBase™ pathway maps, a comprehensive ontology of canonical pathways integrating 3-6 signaling pathways which describe biological mechanisms. As shown in the generated integrative network of the obtained results (Figure 2), we mainly identified enriched terms related to oncogenic pathways such as p53 signaling, S1P2 receptor activation signaling and regulation of micro RNA’s in distinct cancer types.

### Model construction

**FIGURE 3:**
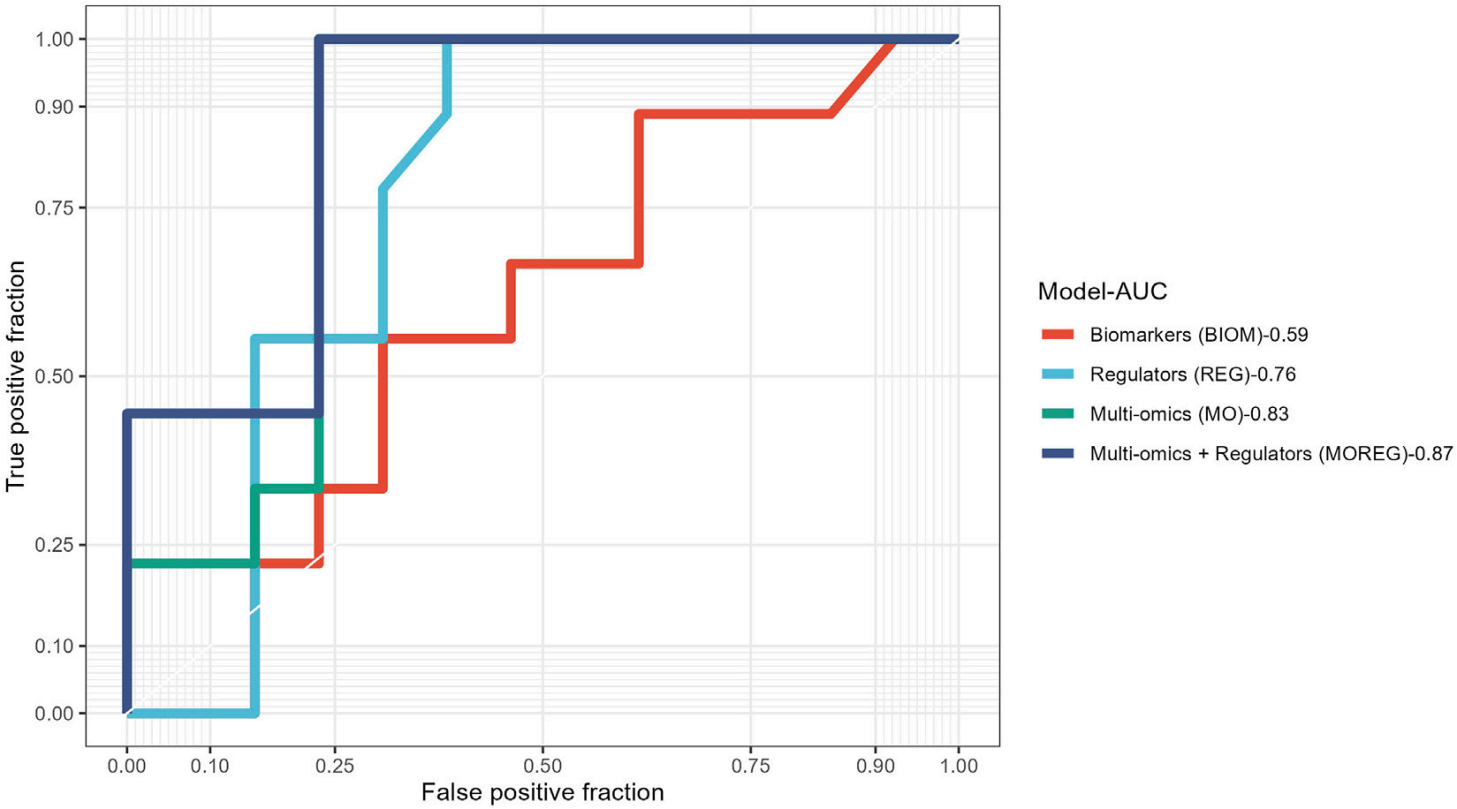
Receiver operating characteristic curves comparing results obtained in the validation of the four different models built using the results obtained from analysis of ACC multi-omics dataset.

To evaluate the discriminative power of the identified multi-omics signature and their topological regulators extracted using systems biology approaches, we trained different random forest classifiers. The built model from the multi-omics signature (MO-Model) included 157 features: 46 genes, 46 micro RNA’s, 65 methylation sites. A second model (REG-Model), corresponding to the identified regulators, was built using 53 genes. A third model (MOREG-Model) combined the features from two previous ones. Finally, a last model (BIOM-Model) was built using known disease biomarkers available in the literature (Xing et al., 2019), accounting for 14 genes: *AURKA*, *TYMS*, *MAD2L1*, *GINS1*, *RACGAP1*, *RRM2*, *EZH2*, *PRC1*, *ZWINT*, *CDK1*, *CCNB1*, *SMC4*, *NCAPG*, and *TPX2*. Details from the training process on 55 samples, and the evaluation using the remaining 22 samples can be found in Table 6. MO and MOREG models are the best performers during the training process (ROC >0.9), followed by the BIOM model, being REG model the one with the worse performance due to its poor specificity (Figure 3). In the cross-validation, MOREG model clearly outperformed the rest of the models with a ROC value of 0.87. The MO model still showed a high discriminative power (ROC = 0.83), while the REG model surpassed the performance of the BIOM model.

**TABLE 6:** Summary statistics for random forest models.

	Training			Cross-validation		
Model name	ROC	Sensitivity	Specificity	ROC	Sensitivity	Specificity
MOREG	0.91	0.85	0.7	0.87	0.56	0.77
MO	0.915	0.87	0.7	0.83	0.56	0.77
REG	0.69	0.8	0.43	0.76	0.22	0.85
BIOM	0.79	0.72	0.73	0.59	0.44	0.69

### Cortellis drug discovery intelligence biomarkers and targets for adrenocortical carcinoma

The biomarkers from the identified multi-omics signature were searched across the CDDI database. Since DNA methylation biomarkers consisted of CpG-gene pairs or trios, we scanned these across our database separately for CpGs and their associated genes, making up a total of 266 input biomarkers for our search.

**FIGURE 4:**
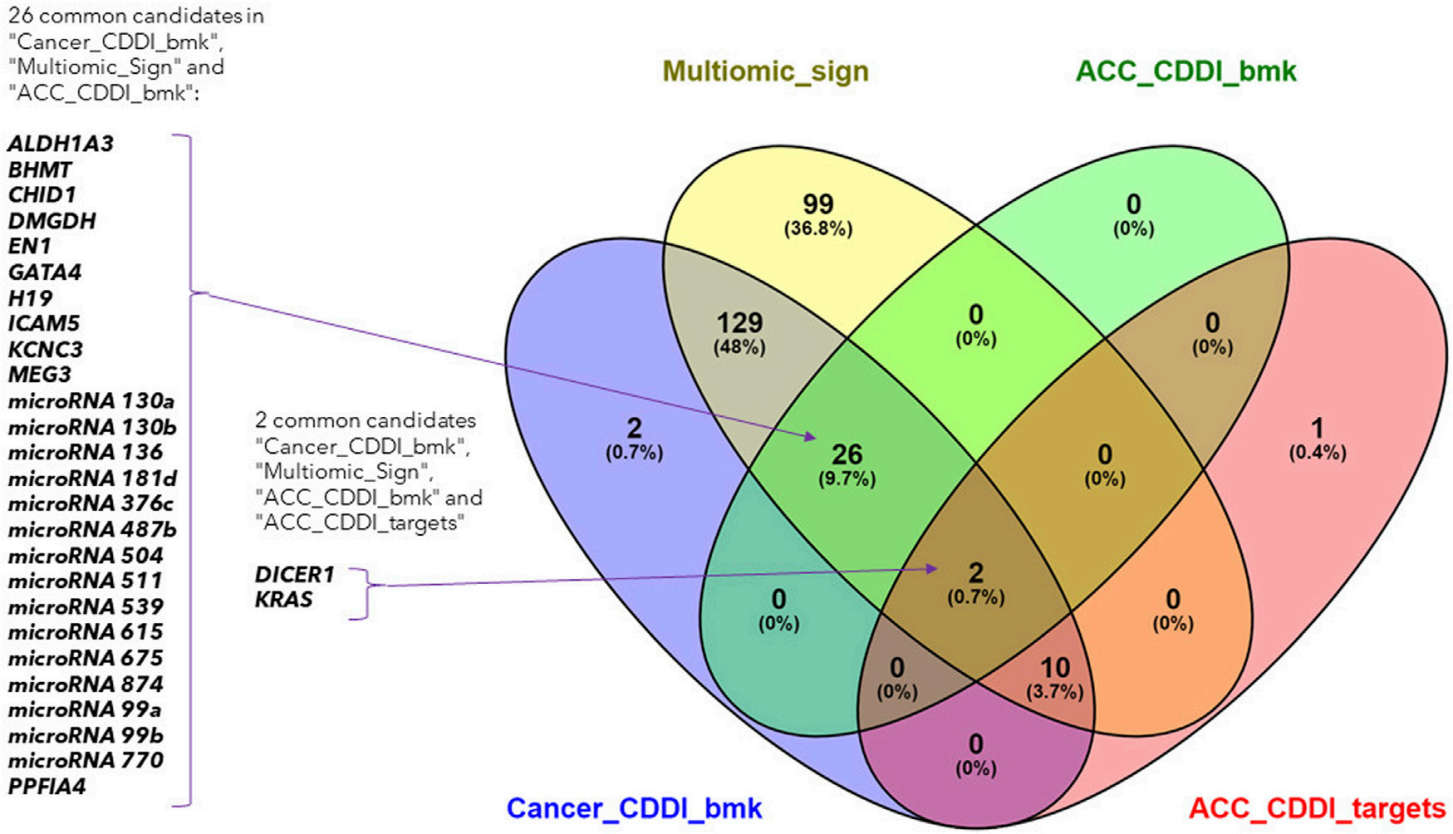
Venn diagram of the lists of ACC biomarkers, ACC targets, cancer biomarkers and the identified multi-omics signature.

The search led to the identification of 28 ACC biomarkers, 167 cancer biomarkers and 12 ACC targets overlapping with the features from the identified multi-omics signature (Figure 4). Out of the 167 cancer biomarkers, 26 are in experimental or early studies in human stages for ACC. Interestingly, we identified 13 ACC targets in CDDI, from which 12 are found in our multi-omics signature, including *DICER1* and *KRAS* as specific ACC biomarkers that were identified using systems biology tools. The presence of *DICER1* mutations in endocrine cancers calls for more research given the lack of effective treatments. In addition, novel therapeutic strategies, including targeted therapies such as tyrosine kinase inhibitors (TKIs), are starting to be studied for ACC.

Interestingly, 99 features from the multi-omics signature did not overlap with CDDI data and could represent potential new disease biomarkers and targets. In addition, the 12 features from our multi-omics signature that overlap with ACC targets are particularly interesting. These findings emphasize the applicability of CDDI to identify known biomarkers and/or targets for which novel drugs are approved or are under study, where there is potential for drug repositioning for these rare and understudied tumors. Details about the multi-omics signature features included in CDDI are available in Supplementary Table S3.

### Survival analysis and construction of a prognostic signature

The prognostic value of the features identified in our analysis was further validated using data from an external dataset with available multi-omics data (GSE49280). Specifically, the selected study contains omics data from gene expression (transcripts and micro RNA’s) and DNA methylation, with associated clinical data including survival information. Some samples were not matched across omics datasets: DNA methylation was available for 81 patients; micro RNA data was available for 78 patients and mRNA data was available for 44 patients. For the former data type, only 3 methylation sites from the identified signature were found to overlap with the methylation data from the external dataset, due to the low throughput of the platform used in GSE49277 study (27.578 CpG sites) compared to the platform from the GDC TCGA ACC dataset (450.000 CpG sites).

We generated individual Kaplan-Meier curves for each gene, micro RNA and DNA methylation sites identified in the multi-omics signature. The obtained curves are available as Supplementary Data (KM curves methylation, KM curves micro RNA’s, KM curves genes) (survival time is reported in months). Regarding multi-omics features corresponding to genes, high expression levels of *HAUS8*, *PLXNA1*, *SHB*, *UBE2S*, *DDX39A, DCAF15*, *a*nd *TSPYL4* showed the most statistically significant associations with a lower survival probability (*p*-value <0.01), whereas *N4BP2L1* was the only gene for which a low expression level was associated with higher mortality. For the analyzed multi-omics biomarkers corresponding to micro RNA’s, high expression levels of *microRNA 376c, microRNA 504* and *microRNA 615* were associated with lower survival probability (*p*-value <0.01), and only *microRNA 1258* conferred a protective effect at lower expression levels. Any of the 3 CpG sites screened showed statistically significant association with OS. Interestingly, most of these features are found in the generated integrative network (Figure 2). The same analysis was run, this time adjusting the model with the Weiss score and Age (gender was excluded due the overrepresentation of female samples in the dataset). The adjusted *p*-values of the obtained hazard ratios (HR) were still significant (*p*-value <0.01) for *HAUS8*, *PLXNA1, DDX39A* and *microRNA 1258.* In contrast, only a new feature, corresponding to a methylation site, achieved a statistically significant HR after adjustment: cg25836301 (*MEG3*). The results are presented in Table 7.

**TABLE 7:** Univariate and multivariate cox regression results for survival analysis. The features below are included in the multi-omics signature and were also available in the validation dataset (GSE49280).

Feature	Unadjusted		Adjusted	
	HR (95% CI)	p	HR (95% CI)	p
HAUS8	0.09 (0.03–0.32)	0	0.18 (0.05–0.71)	0.01
hsa-mir-1258	14.93 (3.36–66.37)	0	7.27 (1.56–33.89)	0.01
SHB	0.18 (0.06–0.56)	0	0.32 (0.1–0.98)	0.05
UBE2S	0.21 (0.08–0.57)	0	0.36 (0.12–1.13)	0.08
DDX39A	0.17 (0.06–0.48)	0	0.38 (0.12–1.22)	0.1
PLXNA1	0.23 (0.08–0.64)	0.01	0.19 (0.06–0.58)	0
hsa-mir-376c	0.22 (0.07–0.65)	0.01	0.31 (0.1–0.99)	0.05
hsa-mir-504	0.28 (0.1–0.78)	0.01	0.34 (0.11–1.06)	0.06
TSPYL4	0.24 (0.09–0.68)	0.01	0.38 (0.13–1.1)	0.07
hsa-mir-615	0.16 (0.04–0.7)	0.01	0.31 (0.07–1.39)	0.13
N4BP2L1	3.41 (1.28–9.08)	0.01	1.72 (0.55–5.41)	0.35
DCAF15	0.3 (0.11–0.79)	0.01	0.73 (0.25–2.17)	0.58
cg25836301	2.53 (1.13–5.64)	0.02	5.41 (2.2–13.3)	0
hsa-mir-874	3.04 (1.21–7.61)	0.02	2.75 (1–7.53)	0.05
SNORD113-3	0.3 (0.11–0.79)	0.02	0.37 (0.13–1.03)	0.06
hsa-mir-381	0.3 (0.11–0.8)	0.02	0.46 (0.16–1.31)	0.15
B4GALT3	0.29 (0.1–0.81)	0.02	0.47 (0.16–1.37)	0.17
DUSP12	0.31 (0.12–0.82)	0.02	0.57 (0.21–1.54)	0.27
MEG9	0.35 (0.13–0.93)	0.03	0.39 (0.14–1.04)	0.06
GATA4	0.34 (0.13–0.89)	0.03	0.49 (0.18–1.36)	0.17
hsa-mir-5690	3.94 (1.14–13.61)	0.03	1.52 (0.4–5.72)	0.54
KANSL1-AS1	2.85 (1.02–7.94)	0.04	1.57 (0.55–4.53)	0.4
hsa-mir-487b	0.36 (0.13–1.01)	0.05	0.42 (0.15–1.2)	0.11
CLMP	0.39 (0.15–1)	0.05	0.74 (0.26–2.07)	0.56
TUBB4B	0.37 (0.14–0.99)	0.05	0.88 (0.27–2.9)	0.84
SNORD114-3	0.44 (0.17–1.12)	0.08	0.5 (0.19–1.33)	0.17
ZUP1	0.43 (0.17–1.11)	0.08	0.65 (0.24–1.72)	0.38
C11orf1	2.15 (0.84–5.48)	0.11	2.26 (0.81–6.31)	0.12
FRAT2	0.46 (0.18–1.19)	0.11	0.53 (0.2–1.39)	0.19
MIR770	0.48 (0.19–1.23)	0.13	1 (0.35–2.85)	0.99
hsa-mir-466	2.15 (0.77–5.97)	0.14	1.62 (0.57–4.65)	0.37
MEG3	0.52 (0.2–1.33)	0.17	0.57 (0.21–1.54)	0.27
hsa-mir-181d	1.84 (0.74–4.59)	0.19	1.34 (0.51–3.51)	0.55
VWA5B2	1.7 (0.67–4.33)	0.26	2.82 (1.08–7.36)	0.03
cg16488098	0.64 (0.27–1.54)	0.32	0.74 (0.3–1.78)	0.5
hsa-mir-511	2.08 (0.48–9.05)	0.33	0.76 (0.16–3.59)	0.73
SIAE	1.55 (0.63–3.84)	0.34	0.93 (0.35–2.52)	0.89
YJEFN3	0.66 (0.27–1.65)	0.38	0.66 (0.25–1.78)	0.42
ST6GALNAC4	0.66 (0.27–1.66)	0.38	0.7 (0.28–1.77)	0.45
GALNS	0.67 (0.27–1.67)	0.39	0.62 (0.24–1.57)	0.31
cg25063710	1.4 (0.62–3.16))	0.42	1.08 (0.47–2.46)	0.86
hsa-mir-216a	1.43 (0.58–3.51)	0.44	1.23 (0.49–3.05)	0.66
JTB	1.43 (0.58–3.58)	0.44	0.9 (0.34–2.35)	0.83
PAQR5	0.7 (0.28–1.76)	0.46	0.71 (0.26–1.93)	0.51
ASF1A	1.41 (0.57–3.53)	0.46	0.92 (0.36–2.35)	0.86
CLASRP	0.74 (0.3–1.83)	0.51	0.62 (0.23–1.7)	0.35
hsa-mir-217	1.35 (0.54–3.37)	0.52	1.12 (0.44–2.88)	0.81
hsa-mir-4521	0.74 (0.29–1.89)	0.53	1.38 (0.52–3.69)	0.52
KCNJ14	1.34 (0.54–3.34)	0.53	1.08 (0.43–2.7)	0.88
STAC3	1.23 (0.5–3.05)	0.65	1.34 (0.53–3.39)	0.53
CAP2	1.23 (0.5–3.02)	0.66	0.9 (0.35–2.34)	0.84
RNPEP	0.82 (0.33–2.02)	0.67	0.37 (0.14–0.99)	0.05
hsa-mir-3912	1.17 (0.47–2.91)	0.74	1.41 (0.54–3.69)	0.48
UGGT2	0.87 (0.35–2.14)	0.76	0.98 (0.39–2.47)	0.96
hsa-mir-1179	1.13 (0.46–2.79)	0.79	1.06 (0.42–2.69)	0.9
hsa-mir-4326	0.9 (0.36–2.23)	0.81	1.48 (0.57–3.83)	0.42
CHID1	0.91 (0.37–2.24)	0.83	0.78 (0.31–1.95)	0.59
AVPR1A	1.1 (0.45–2.72)	0.83	0.81 (0.31–2.16)	0.68
hsa-mir-190b	1.1 (0.44–2.75)	0.83	0.91 (0.35–2.39)	0.85
ACSS2	1.08 (0.43–2.69)	0.87	1.13 (0.44–2.92)	0.8
TEDC1	1.07 (0.43–2.64)	0.88	1.47 (0.57–3.76)	0.43
D2HGDH	1.06 (0.43–2.62)	0.9	0.81 (0.3–2.23)	0.69
PRELID3A	1 (0.4–2.45)	0.99	0.66 (0.25–1.7)	0.39

**FIGURE 5:**
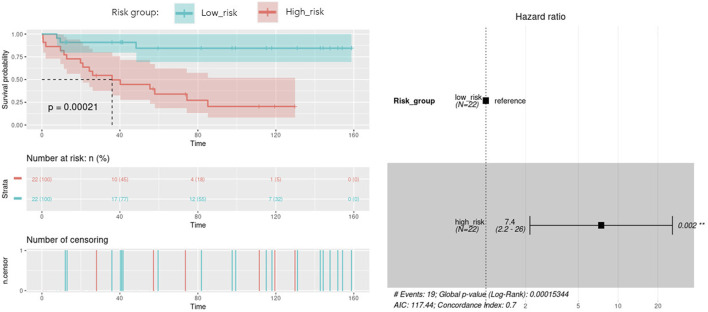
Kaplan-Meier curve with optimal features from the identified multi-omics signature (OptMultiSig**)** using OS from high and low risk ACC patients in the external dataset. A Forest plot with Hazard Ratio information is included.

With the aim of building a prognostic signature of ACC disease including the most predictive features obtained from multi-omics integration, and thus with potential application in clinical practice, we run a model to identify the combination of the previous features significantly associated with overall survival. By calculating and comparing the AIC scores of the several possible models, we selected the best-fit model as the one explaining the greatest amount of variation using the fewest possible variables and lower AIC. A total of 9 features involving 5 genes and 4 micro RNAs showed the optimal AIC value and were subsequently used to establish a risk score using the formula: OptMultiSig risk score = [1.958 x *EXP* GATA4]+[-22.285 x *EXP* N4BP2L1]+[1.087 x *EXP* KCNJ14]+[3.157 x *EXP* SNORD1143]+[2.271 x *EXP* UBE2S]+[20.908 x *EXP* microRNA 615]+[-1.173 x *EXP* microRNA 1179]+[-19.938 x *EXP* microRNA 217]+[-20.976 x *EXP* microRNA 5690]. According to the median OptMultiSig risk score of 165.575, we classified patients into low and high-risk groups comprising 22 samples each. As shown in Figure 5, high-risk patients showed significant decreased OS as compared to low-risk ACC patients (*p*-value = 2.1 × 10^−4^; HR = 1.13; 95% CI 0,04–0.46), highlighting the utility of the 9 features as an independent prognostic factor of poor prognosis for ACC.

## Discussion

Rare diseases encompass a wide range of disorders, many of which are characterized by limited treatment options, delayed diagnoses, and poor prognoses. ACC, as a rare and aggressive malignancy, exemplifies the challenges faced by patients and healthcare professionals in diagnosing, managing, and treating rare diseases. Prognostic evaluation is critical to determine disease progression and inform treatment decisions. Biomarkers can play a vital role in predicting the clinical course of ACC, stratifying patients into risk groups, and enabling personalized treatment approaches. The identification of staging biomarkers associated with tumor aggressiveness and metastatic potential assists in tailoring surveillance strategies and implementing early interventions in high-risk patients.

Integrative analysis of multi-omics data allows to capture cellular regulation at different layers, thus bringing the possibility to build more robust classifiers of biological samples and to discover new molecular interactions of the underlying diseases. In this study, we integrated multi-omics data from different origin: RNA-seq gene expression, miRNA-seq expression and DNA methylation belonging to the GDC TCGA Adrenocortical Carcinoma (ACC) datasets (Zheng et al., 2016). The available copy number variants were discarded due to their usual association with methylation and gene expression (Shao et al., 2019; Shi et al., 2020), while the somatic mutations were not completely suitable for the DIABLO quantitative approach. After the integration, the performed network analysis identified a specific ACC signature composed of 210 biomarkers (99 genes, 46 micro RNA’s and 65 methylation sites) with a high power to discriminate early and advanced ACC disease stages. A total of 28 features from the obtained multi-omics signature were reported in our CDDI database as biomarkers associated with ACC. Among them, *ALDH1A3, BHMT, DMGDH, EN1, ICAM5, KCNC3* and *PPFIA4* biomarkers coming from DNA methylation data, *CHID1, GATA4, MEG3* and *microRNA 770* from RNA expression data, and *DICER1, H19* and *KRAS* from network analysis. Specifically, *ALDH1A3, BHMT, CHID1, EN1, GATA4, ICAM5* and *KCNC3* appeared as prognostic biomarkers for ACC in experimental stages, and *DICER1* in early studies in humans. The higher amount of DNA methylation data derived biomarkers associated with ACC disease and the methylation association with other processes (e.g., CNV) might be behind the high amount of variance explained by the five components obtained using DNA methylation data during the sPLS-DA model construction.

In the case of the third component, all the 25 described RNA-seq, miRNA-seq and DNA methylation features (with the only exception of *C11orf1* and the cg12138102 methylation site) belong to the genomic location where the imprinted differentially methylated region MEG3:TSS-DMR is located (Hernandez Mora et al., 2018). The expressed copy of the genes present in this region is always the one inherited from the mother due to the specific methylation of the CpGs on the father’s copy. This region has been already associated with hepatocarcinoma, where in fact the methylation differences were due to copy number alterations caused by the loss of the mother’s copy (Martin-Trujillo et al., 2017). MEG3 is an inhibitor of the cell proliferation, interacting, for example, with p53, and the likely loss of the expressed copy (reflected on the expression of genes and micro RNA’s and methylation changes) would unsurprisingly imply an effect on the variance and stage classification of the ACC tumors.

A topological regulator identified through the network analysis, the H19 imprinted maternally expressed transcript, is also located very close to the IGF2 well-known ACC biomarker, belonging both to a genome region also regulated by imprinting (Hernandez Mora et al., 2018). Belonging to this region, there are also the well-known MIR483 predictive ACC biomarker (Chabre et al., 2013) and the MIR675 detected in component 4. Again, it would be not surprising that any methylation change in this region, and their derived abnormal co-expression, could have some impact on molecular networks regulated by any of them, highlighting the utility of our systems biology-based approach combining multi-omics data to unravel novel mechanisms leading to tumorigenesis. Furthermore, a total of 14 microRNAs from the multi-omics signature are biomarkers for ACC in experimental or early studies in human stages as extracted from CDDI data. Together with DNA methylation, micro RNA aberrant expression offers an additional layer of epigenetic control that could be used as therapeutic target.

An external dataset with available multi-omics data was leveraged as a validation dataset to evaluate the performance of the identified biomarkers as a prognostic signature. Univariate Cox regression analysis was independently performed using the identified biomarkers. High expression of *HAUS8*, *PLXNA1*, *SHB*, *UBE2S*, *DDX39A, DCAF15, TSPYL4*, *microRNA 376c, microRNA 504* and *microRNA 615* was significantly associated with lower overall survival, while patients with low expression of *N4BP2L1* and *microRNA 1258* were associated with decreased survival. Then, a multivariate Cox regression was performed using the Weiss score and age as covariates, confirming the robustness of most of the identified biomarkers for predicting the overall survival in the validation cohort.

Among the multi-omics biomarkers significantly associated with OS, only micro RNA’s were found to be ACC biomarkers in CDDI. Indeed, *microRNA 376c* (Veronez et al., 2022), *microRNA 504* (Koperski et al., 2017) and *microRNA 615* (Assié et al., 2014) have been reported previously as potential biomarker candidates for ACC disease profiling. One of the micro RNA’s significantly associated with ACC survival, *microRNA 376c* was reported in early studies for disease diagnosis(Chabre et al., 2013), and also showed experimental evidence as a potential candidate for ACC disease profiling. However, none of the genes influencing OS have been previously associated with ACC and might represent potential novel disease biomarkers. Concordantly, *SHB* expression was associated with shorter survival time, co-expression of immune cell and vascular related genes in human Acute Myeloid Leukemia (AML) (Jamalpour et al., 2017). *UBE2S* was found to be aberrantly expressed in almost all human cancers in a previous pan-cancer study, and elevated *UBE2S* expression was unfavorably associated with prognosis and pathological stage (Bao et al., 2022). In addition, *N4BP2L1* has been shown to affect the insulin signaling pathway (Watanabe et al., 2021), consistent with the role of IGF2 expression in the pathophysiology of ACC (Pereira et al., 2019).

Then, a multivariate Cox model and step analysis was used for model optimization to define the optimal prognostic signature and found that this signature was a potential independent prognostic factor for ACC patients. The 9-features signature contained 5 genes and 4 micro RNAs with roles in cancer progression. For instance, *GATA4,* identified as an ACC biomarker, is thought to influence proliferation by regulating transcription in several cancer types (Zheng and Blobel, 2010); *KCNJ14* is a biomarker for cancer progression and development (Alasiri, 2023); overexpression of the small nucleolar RNA *SNORD1143* was observed in AML and Acute Promyelocytic Leukemia (APL) (Liuksiala et al., 2014). Regarding micro RNAs, upregulation of *microRNA 615,* also identified as an ACC biomarker, was found to regulate several cancer pathways, and importantly was found to inhibit IGF2 in several cancer types (Godínez-Rubí and Ortuño-Sahagún, 2020). *MicroRNA 1179* was found to inhibit proliferation and invasion in pancreatic cancer cells through the inhibition of *E2F5* (Lin et al., 2018). *MicroRNA 217* showed a tumor suppressor role in pancreatic cancer by downregulating *ATD2* (Dutta et al., 2022); and *microRNA 5690* was also included in a pathological grading signature for lung adenocarcinoma (Yang et al., 2020).

Our proposed workflow is key for rare diseases such as ACC due of the limited number of studies. Using the transversal power that the use of multiple OMICS can bring, we could combine different little evidence from scarce data, resulting in prognostic value and potential translation into clinical research and diagnostics. Although subtle correlations were found between the identified multi-omics components and important ACC clinical parameters, the obtained coefficients are affected by the limited sample size of the study, and a larger cohort can undoubtedly strengthen such associations. In addition, we showed that network analysis can expand the discovery of important molecular players in diseases.

## Conclusion

These results demonstrate the usefulness of combining Clarivate´s systems biology tools with molecular signatures derived from multi-omics experiments to identify biologically meaningful biomarkers. Given the low prevalence of ACC, large and comprehensive studies are missing to fully understand the molecular alterations and the relevant signaling pathways altered in these patients. The use of multi-omics and systems biology methods can identify new targets or biomarkers that could be clinically relevant in the form of molecular diagnostic tools such as quantitative polymerase chain reaction (qPCR). Owing to the high sensitivity and specificity of qPCR, together with its multiplexing capacity, the identified prognostic multi-omics signature could be used in clinical practice to tailor clinical decisions to individual ACC patient prognostic profiles, based on accurate characterization of the expression profile of included genes and microRNAs. The presented workflow contributes to improving disease stratification and treatment decision support not only for ACC, but also for other rare diseases with a limited amount of data available.

## Data Availability

The original contributions presented in the study are included in the article/Supplementary Material, further inquiries can be directed to the corresponding author.
