# Supplementary material for: Machine learning combining multi-omics data and network algorithms identifies adrenocortical carcinoma prognostic biomarkers
Source: Front Mol Biosci. 2023 Nov 6;10:1258902. doi: 10.3389/fmolb.2023.1258902 (PMC10658191; doi:10.3389/fmolb.2023.1258902)
Supplement: Supplementary file 1 [file DataSheet2.PDF]

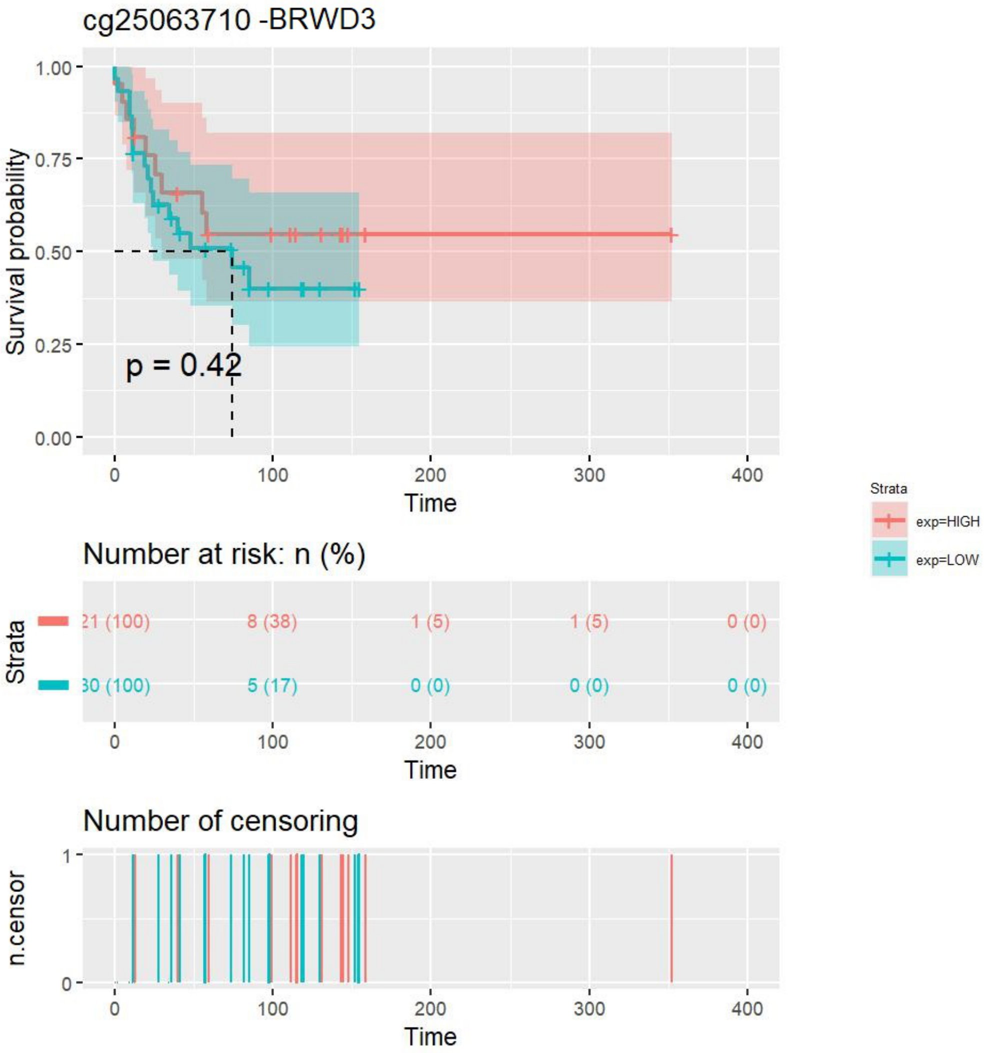
cg25063710 -BRWD3
Survival probability
1.00
0.75
0.50
0.25
0.00
p = 0.42
0
100
200
300
400
Time
Strata
exp=HIGH
exp=LOW
Number at risk: n (%)
Strata
21 (100)
8 (38)
1 (5)
1 (5)
0 (0)
30 (100)
5 (17)
0 (0)
0 (0)
0 (0)
Time
Number of censoring
n.censor
1
0
Time

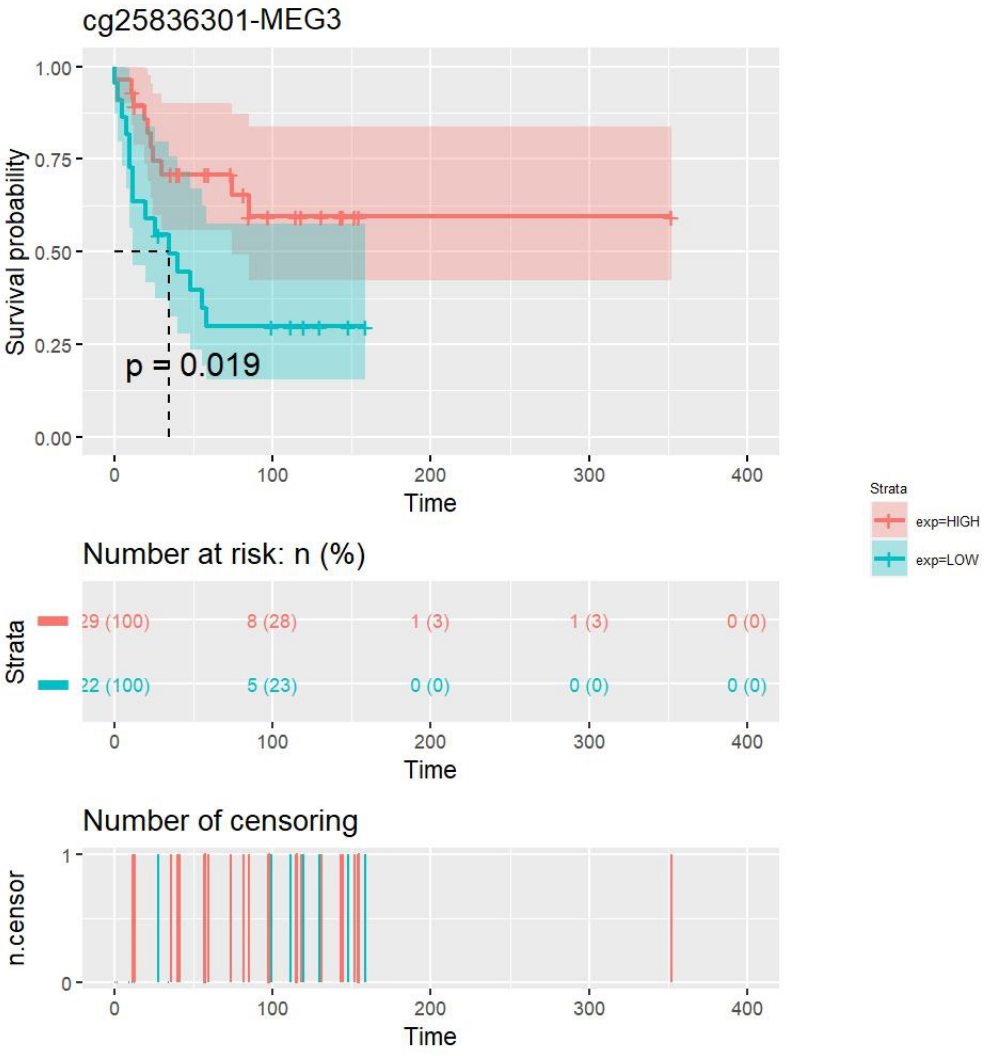

cg25836301-MEG3
Survival probability
1.00
0.75
0.50
0.25
0.00
p = 0.019
0
100
200
300
400
Time
Strata
exp=HIGH
exp=LOW
Number at risk: n (%)
Strata
29 (100)
8 (28)
1 (3)
1 (3)
0 (0)
22 (100)
5 (23)
0 (0)
0 (0)
0 (0)
0
100
200
300
400
Time
Number of censoring
n.censor
1
0
0
100
200
300
400
Time

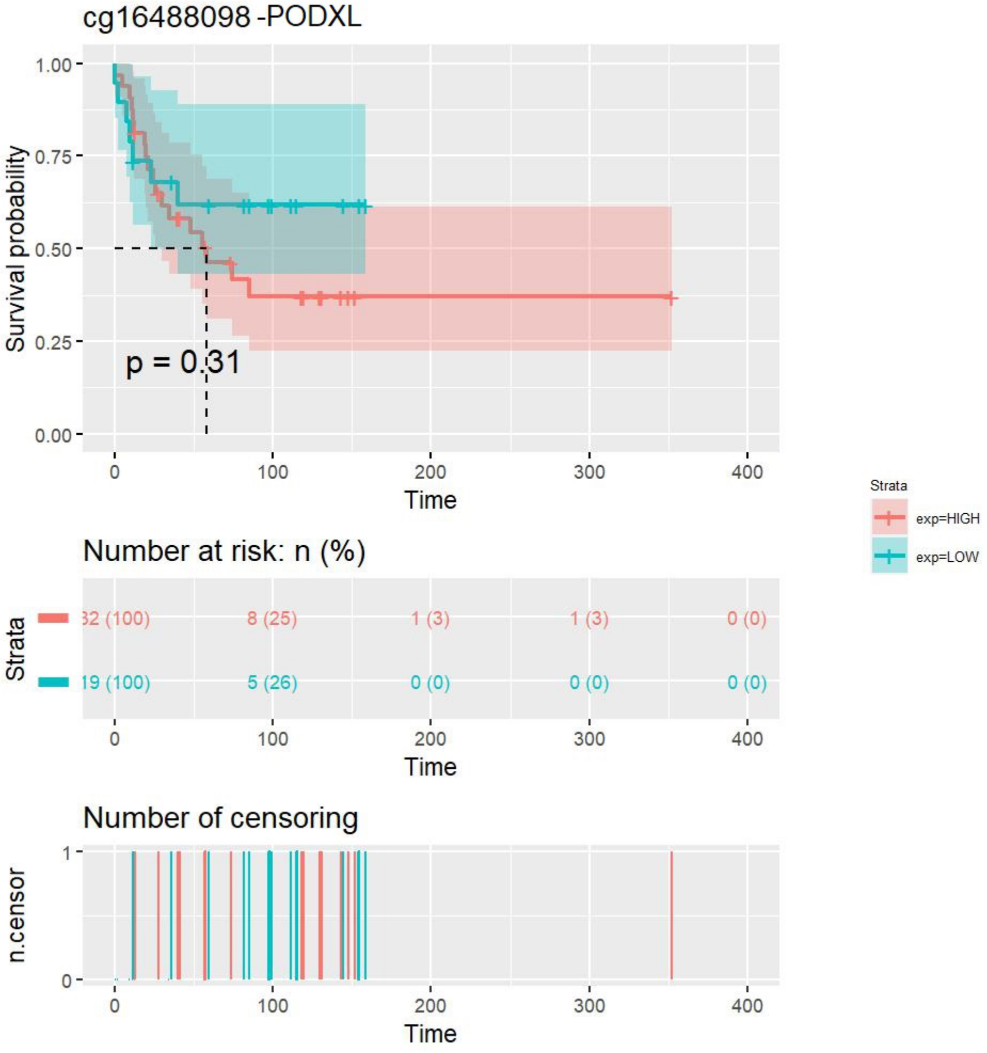

cg16488098 -PODXL
Survival probability
1.00
0.75
0.50
0.25
0.00
p = 0.31
0
100
200
300
400
Time
Strata
exp=HIGH
exp=LOW
Number at risk: n (%)
Strata
32 (100)
8 (25)
1 (3)
1 (3)
0 (0)
19 (100)
5 (26)
0 (0)
0 (0)
0 (0)
Time
Number of censoring
n.censor
1
0
Time
