# Supplementary figures and images for: Machine learning combining multi-omics data and network algorithms identifies adrenocortical carcinoma prognostic biomarkers
Source: Front Mol Biosci. 2023 Nov 6;10:1258902. doi: 10.3389/fmolb.2023.1258902 (PMC10658191; doi:10.3389/fmolb.2023.1258902)

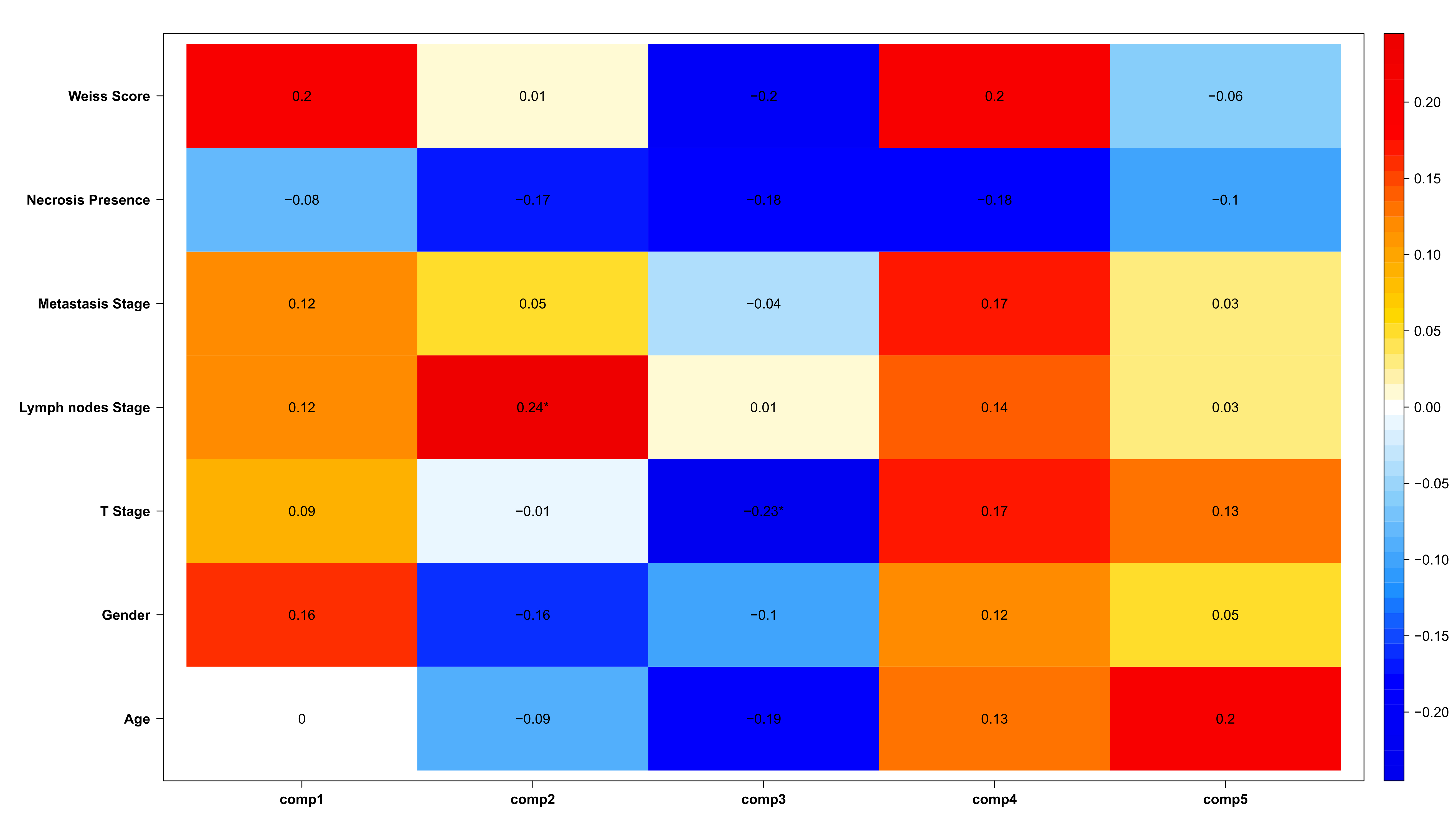

Supplement: Supplementary file 4 [file Image1.JPEG]
