# Supplementary material for: Machine learning combining multi-omics data and network algorithms identifies adrenocortical carcinoma prognostic biomarkers
Source: Front Mol Biosci. 2023 Nov 6;10:1258902. doi: 10.3389/fmolb.2023.1258902 (PMC10658191; doi:10.3389/fmolb.2023.1258902)
Supplement: Supplementary file 5 [file DataSheet3.PDF]

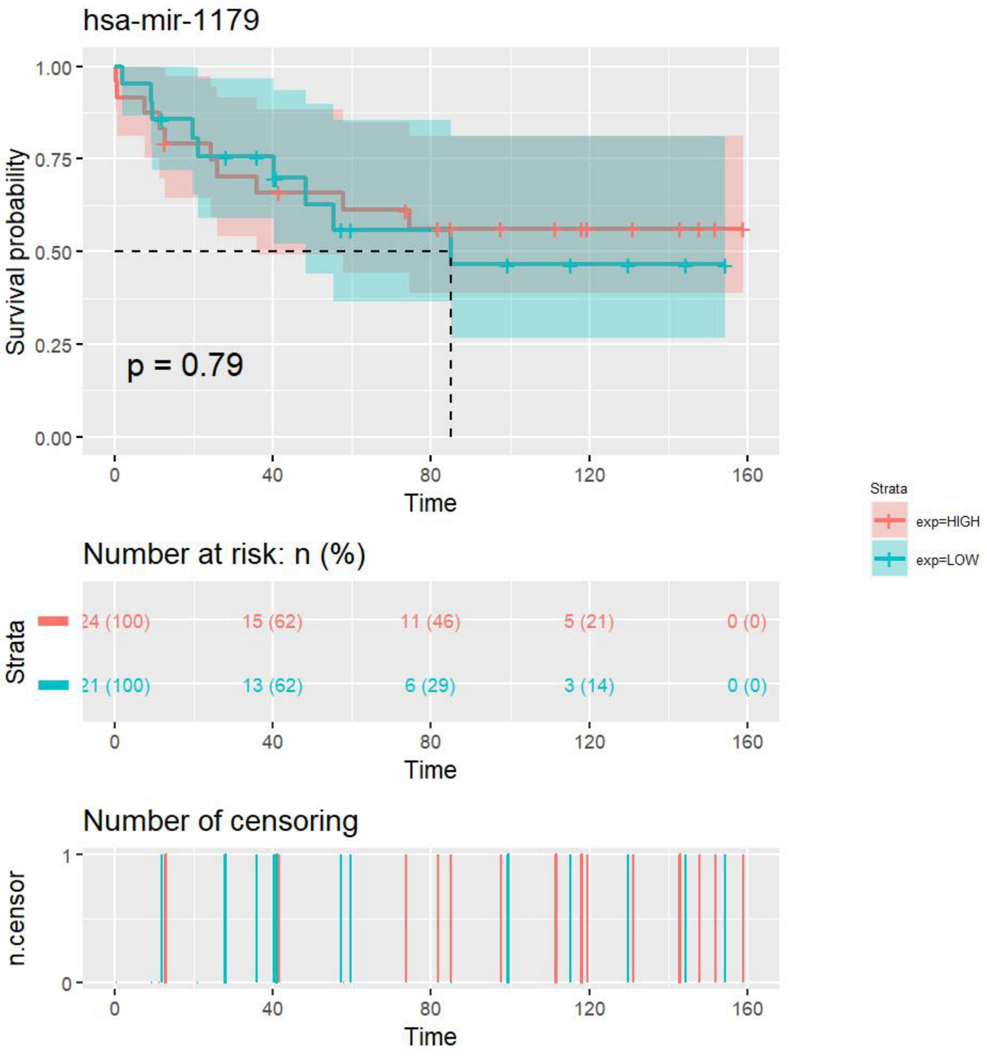

hsa-mir-1179
Survival probability
1.00
0.75
0.50
0.25
0.00
p = 0.79
0
40
80
120
160
Time
Strata
exp=HIGH
exp=LOW
Number at risk: n (%)
Strata
24 (100)
15 (62)
11 (46)
5 (21)
0 (0)
21 (100)
13 (62)
6 (29)
3 (14)
0 (0)
0
40
80
120
160
Time
Number of censoring
n.censor
1
0
0
40
80
120
160
Time

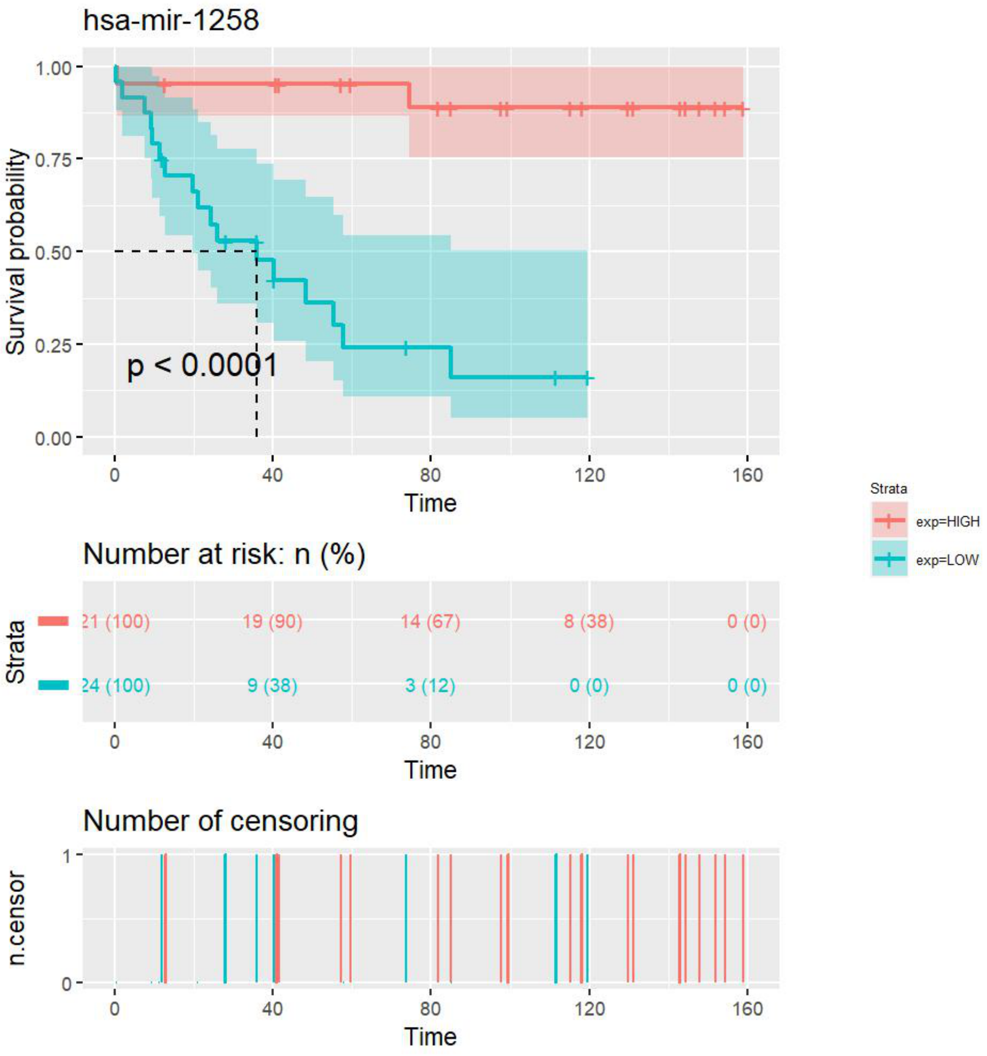
hsa-mir-1258
Survival probability
1.00
0.75
0.50
0.25
0.00
p < 0.0001
0
40
80
120
160
Time
Strata
exp=HIGH
exp=LOW
Number at risk: n (%)
Strata
21 (100)
19 (90)
14 (67)
8 (38)
0 (0)
24 (100)
9 (38)
3 (12)
0 (0)
0 (0)
Number of censoring
n.censor
1
0

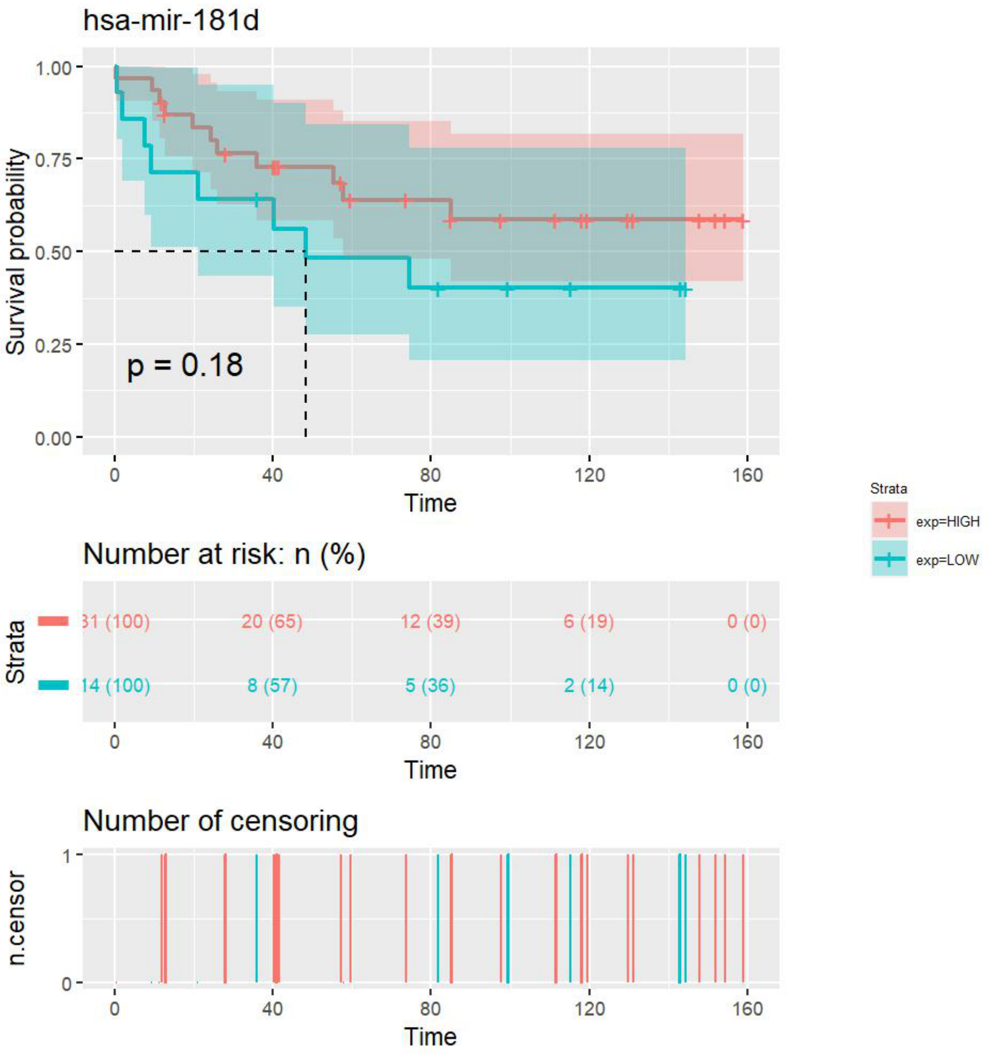

hsa-mir-181d
Survival probability
1.00
0.75
0.50
0.25
0.00
p = 0.18
0
40
80
120
160
Time
Strata
exp=HIGH
exp=LOW
Number at risk: n (%)
Strata
31 (100)
20 (65)
12 (39)
6 (19)
0 (0)
14 (100)
8 (57)
5 (36)
2 (14)
0 (0)
Number of censoring
n.censor
1
0

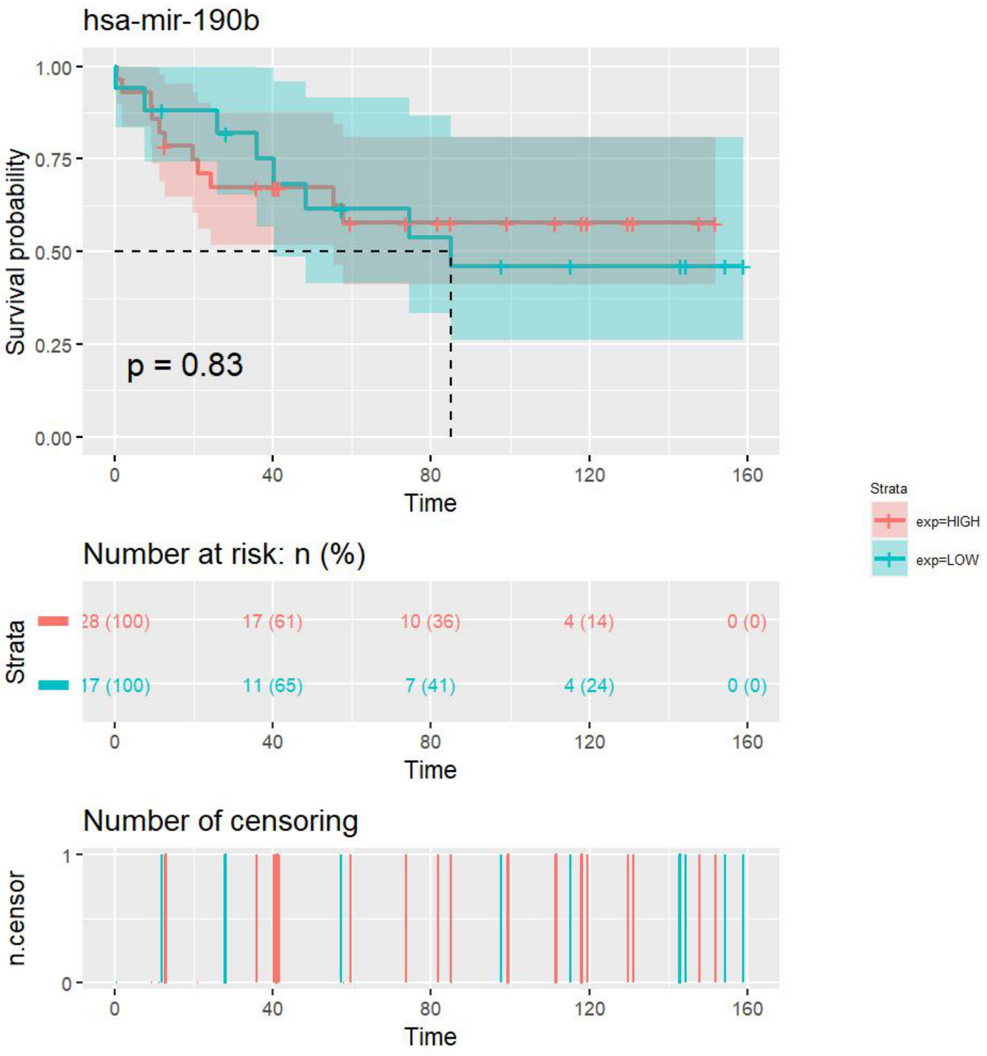
hsa-mir-190b
Survival probability
1.00
0.75
0.50
0.25
0.00
p = 0.83
0
40
80
120
160
Time
Strata
exp=HIGH
exp=LOW
Number at risk: n (%)
Strata
28 (100)
17 (61)
10 (36)
4 (14)
0 (0)
17 (100)
11 (65)
7 (41)
4 (24)
0 (0)
Time
Number of censoring
n.censor
1
0
Time

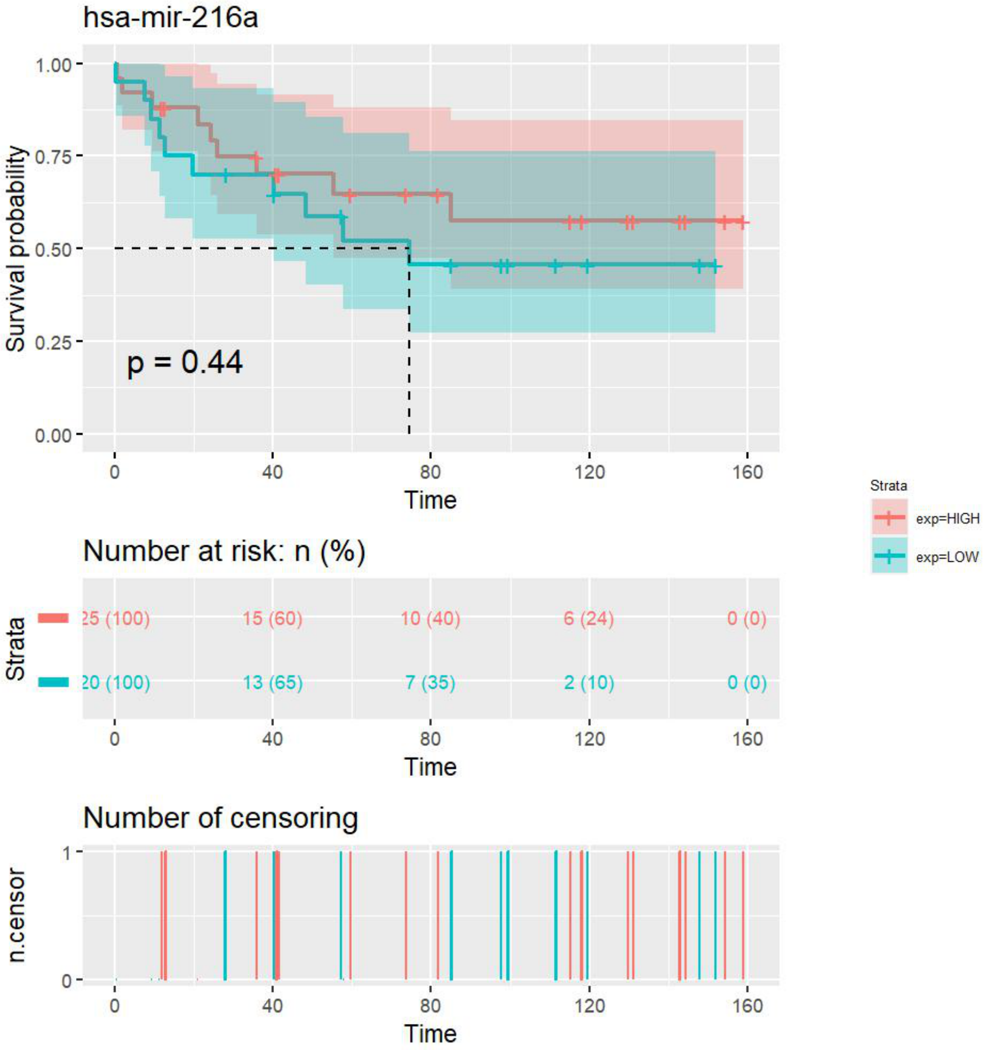

hsa-mir-216a
Survival probability
1.00
0.75
0.50
0.25
0.00
p = 0.44
0
40
80
120
160
Time
Strata
exp=HIGH
exp=LOW
Number at risk: n (%)
Strata
25 (100)
15 (60)
10 (40)
6 (24)
0 (0)
20 (100)
13 (65)
7 (35)
2 (10)
0 (0)
Time
Number of censoring
n.censor
1
0
Time

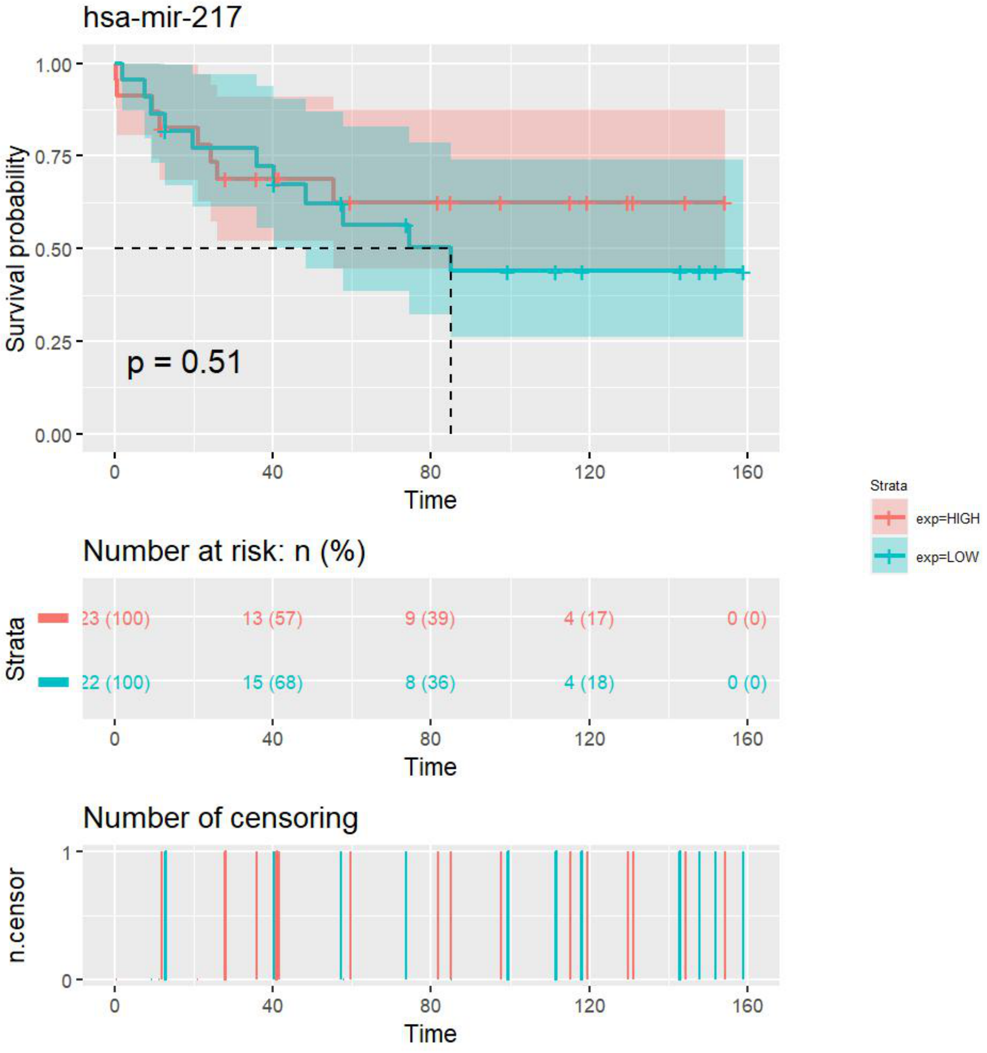

hsa-mir-217
Survival probability
1.00
0.75
0.50
0.25
0.00
p = 0.51
0
40
80
120
160
Time
Strata
exp=HIGH
exp=LOW
Number at risk: n (%)
Strata
23 (100)
13 (57)
9 (39)
4 (17)
0 (0)
22 (100)
15 (68)
8 (36)
4 (18)
0 (0)
Time
Number of censoring
n.censor
1
0
Time

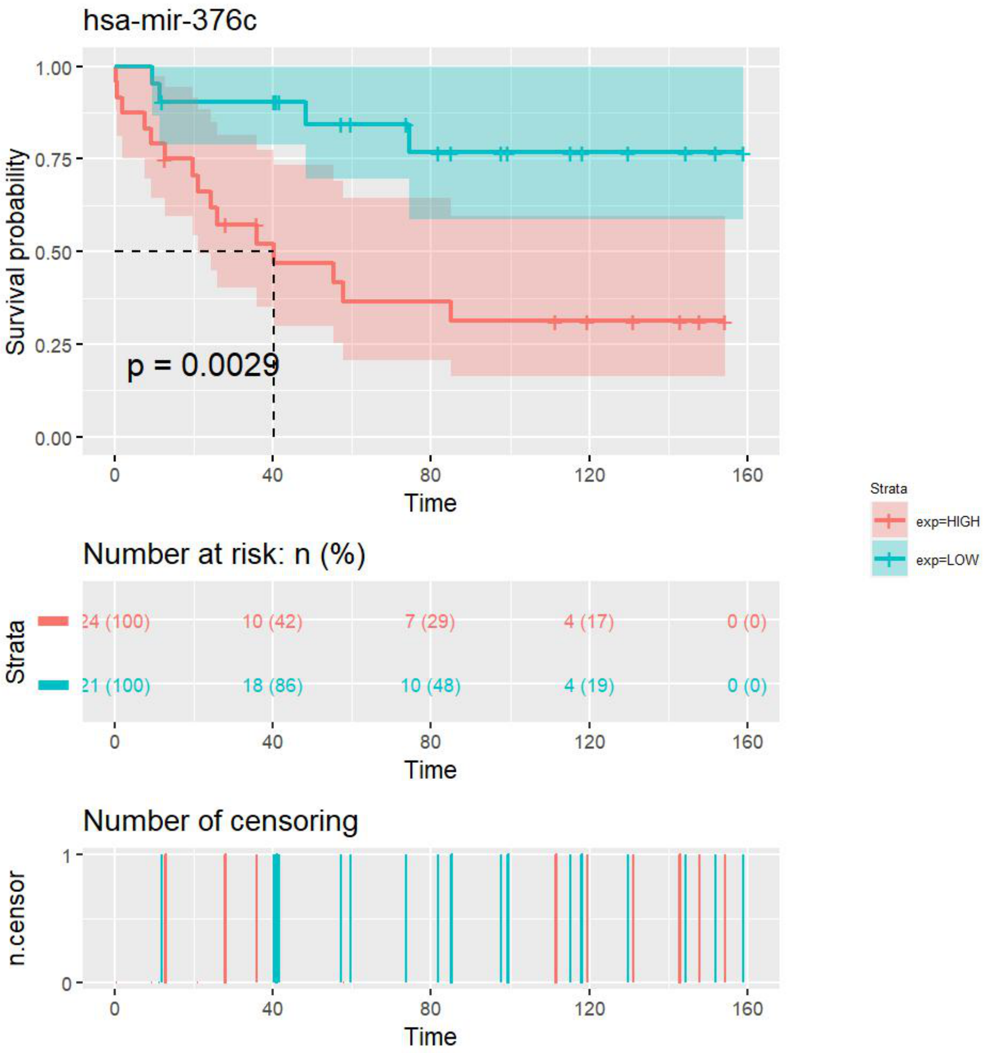
hsa-mir-376c
Survival probability
1.00
0.75
0.50
0.25
0.00
p = 0.0029
0
40
80
120
160
Time
Strata
exp=HIGH
exp=LOW
Number at risk: n (%)
Strata
24 (100)
10 (42)
7 (29)
4 (17)
0 (0)
21 (100)
18 (86)
10 (48)
4 (19)
0 (0)
Time
Number of censoring
n.censor
1
0
Time

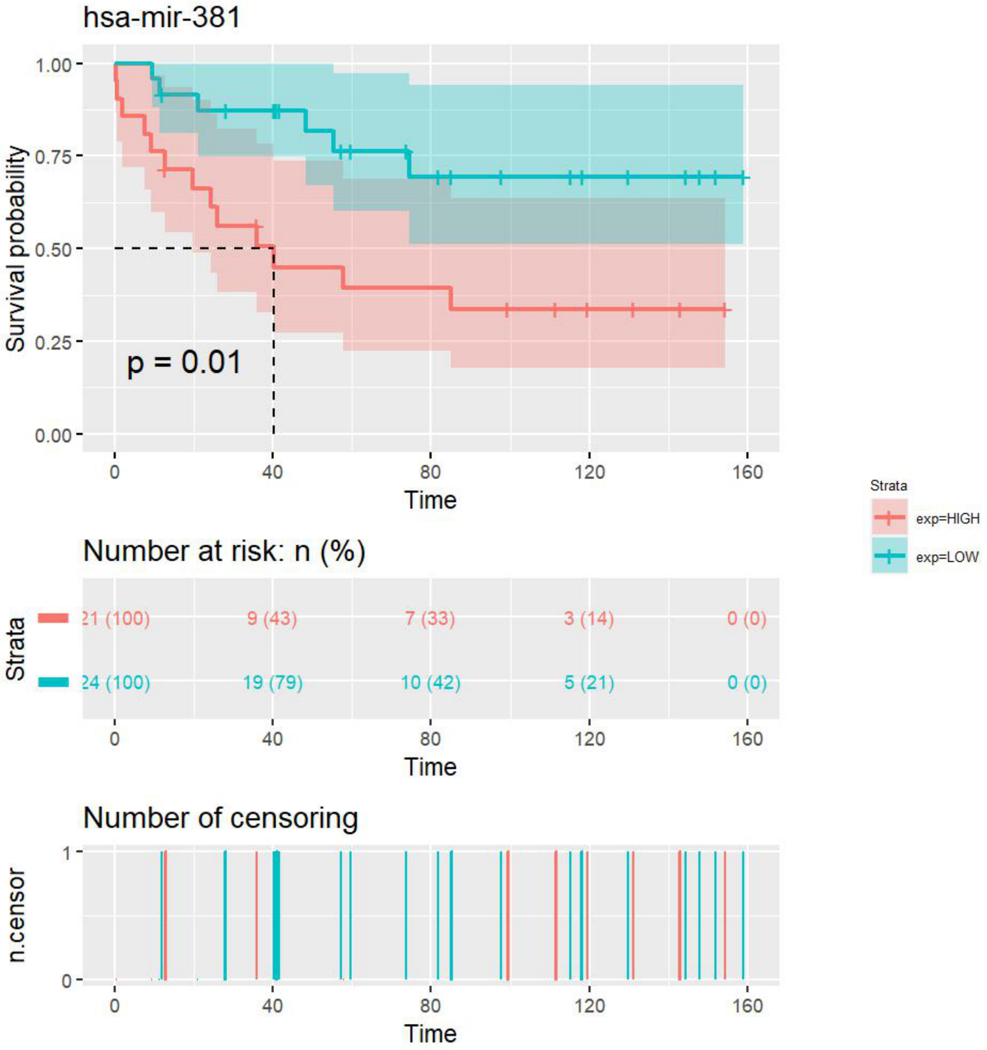

hsa-mir-381
Survival probability
1.00
0.75
0.50
0.25
0.00
p = 0.01
0
40
80
120
160
Time
Strata
exp=HIGH
exp=LOW
Number at risk: n (%)
Strata
21 (100)
9 (43)
7 (33)
3 (14)
0 (0)
24 (100)
19 (79)
10 (42)
5 (21)
0 (0)
Number of censoring
n.censor
1
0

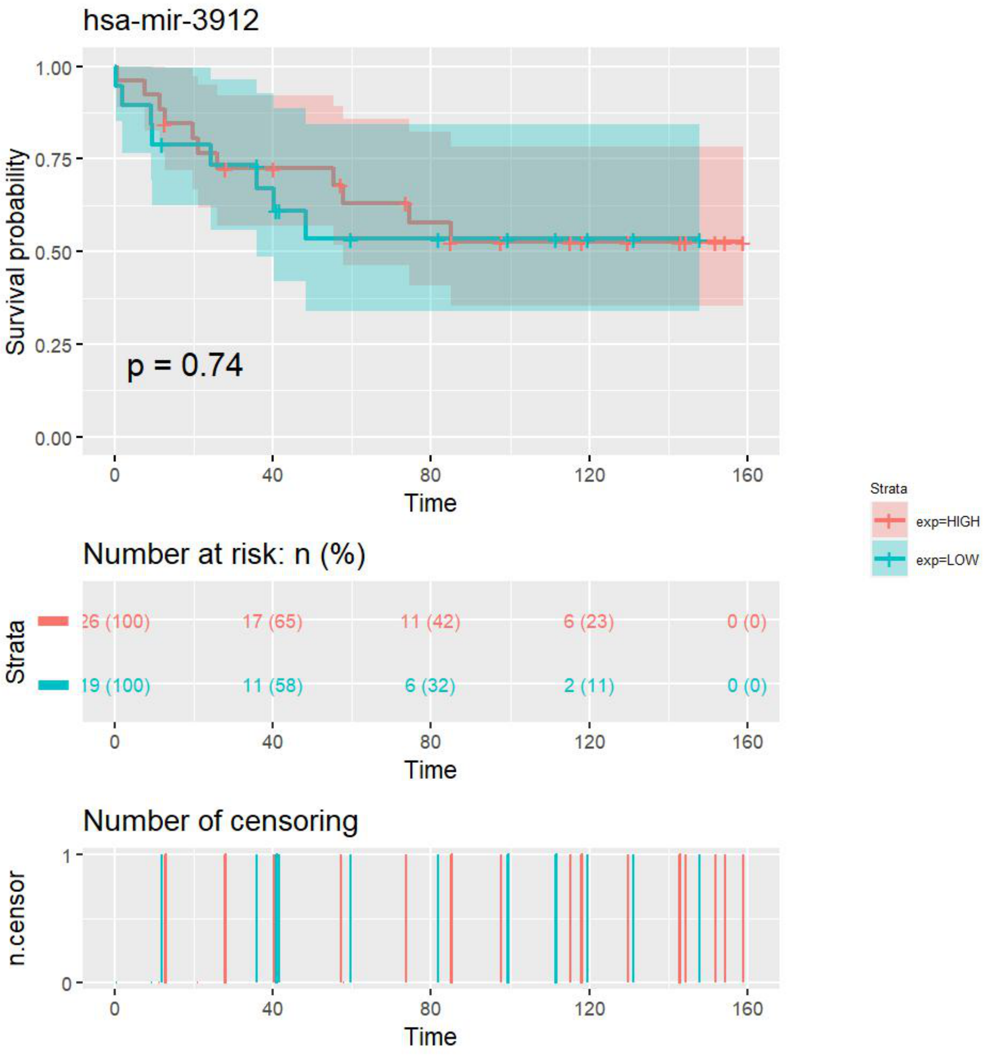
hsa-mir-3912
Survival probability
1.00
0.75
0.50
0.25
0.00
p = 0.74
0
40
80
120
160
Time
Strata
exp=HIGH
exp=LOW
Number at risk: n (%)
Strata
26 (100)
17 (65)
11 (42)
6 (23)
0 (0)
19 (100)
11 (58)
6 (32)
2 (11)
0 (0)
Number of censoring
n.censor
1
0

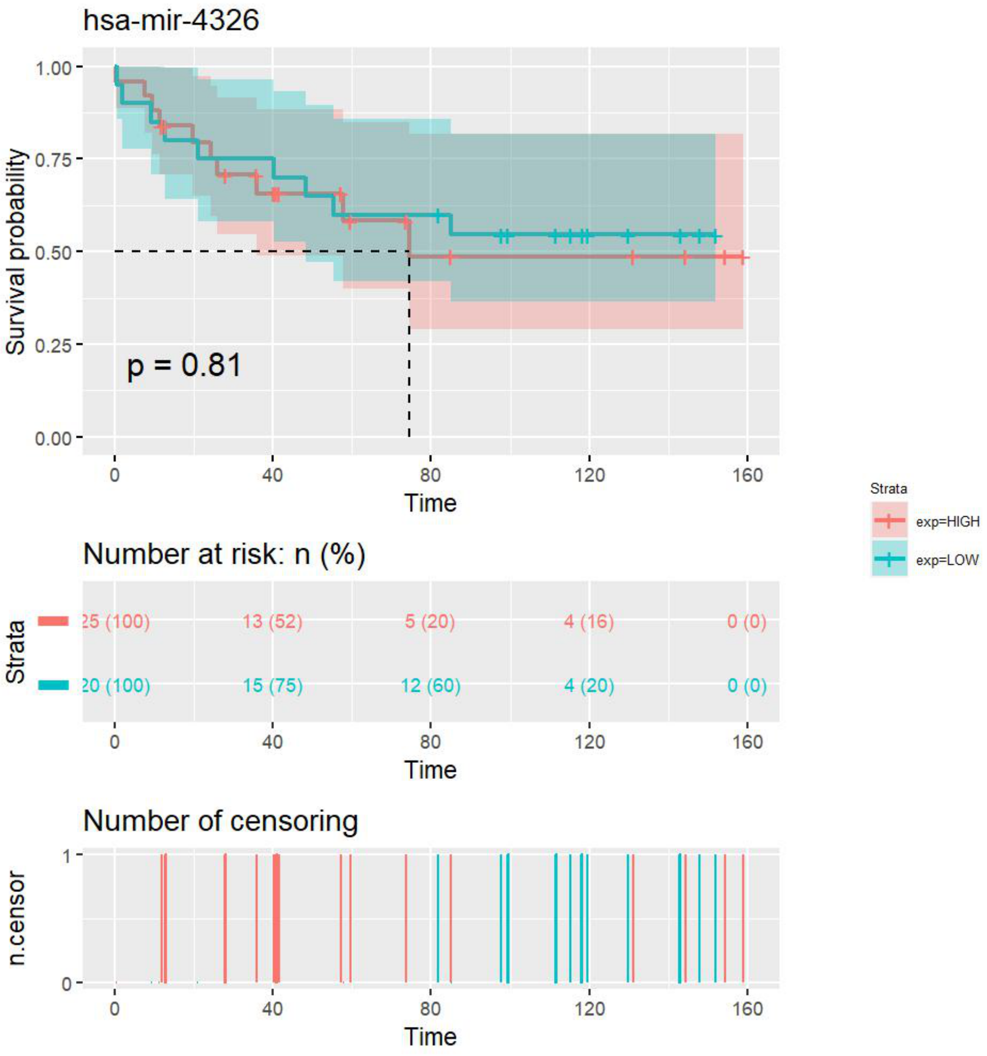
hsa-mir-4326
Survival probability
1.00
0.75
0.50
0.25
0.00
p = 0.81
0
40
80
120
160
Time
Strata
exp=HIGH
exp=LOW
Number at risk: n (%)
Strata
25 (100)
13 (52)
5 (20)
4 (16)
0 (0)
20 (100)
15 (75)
12 (60)
4 (20)
0 (0)
Number of censoring
n.censor

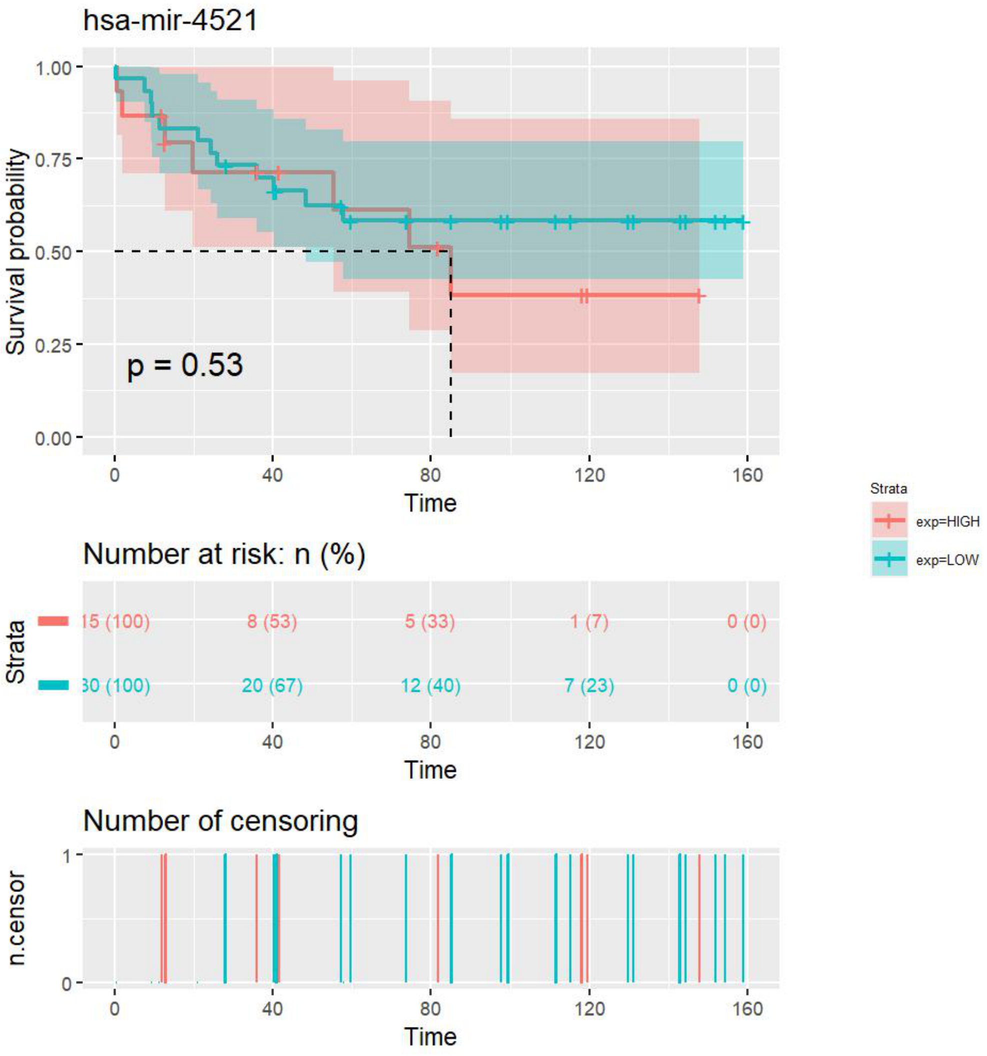

hsa-mir-4521
Survival probability
1.00
0.75
0.50
0.25
0.00
p = 0.53
0
40
80
120
160
Time
Strata
exp=HIGH
exp=LOW
Number at risk: n (%)
Strata
15 (100)
8 (53)
5 (33)
1 (7)
0 (0)
30 (100)
20 (67)
12 (40)
7 (23)
0 (0)
Time
Number of censoring
n.censor
1
0
Time

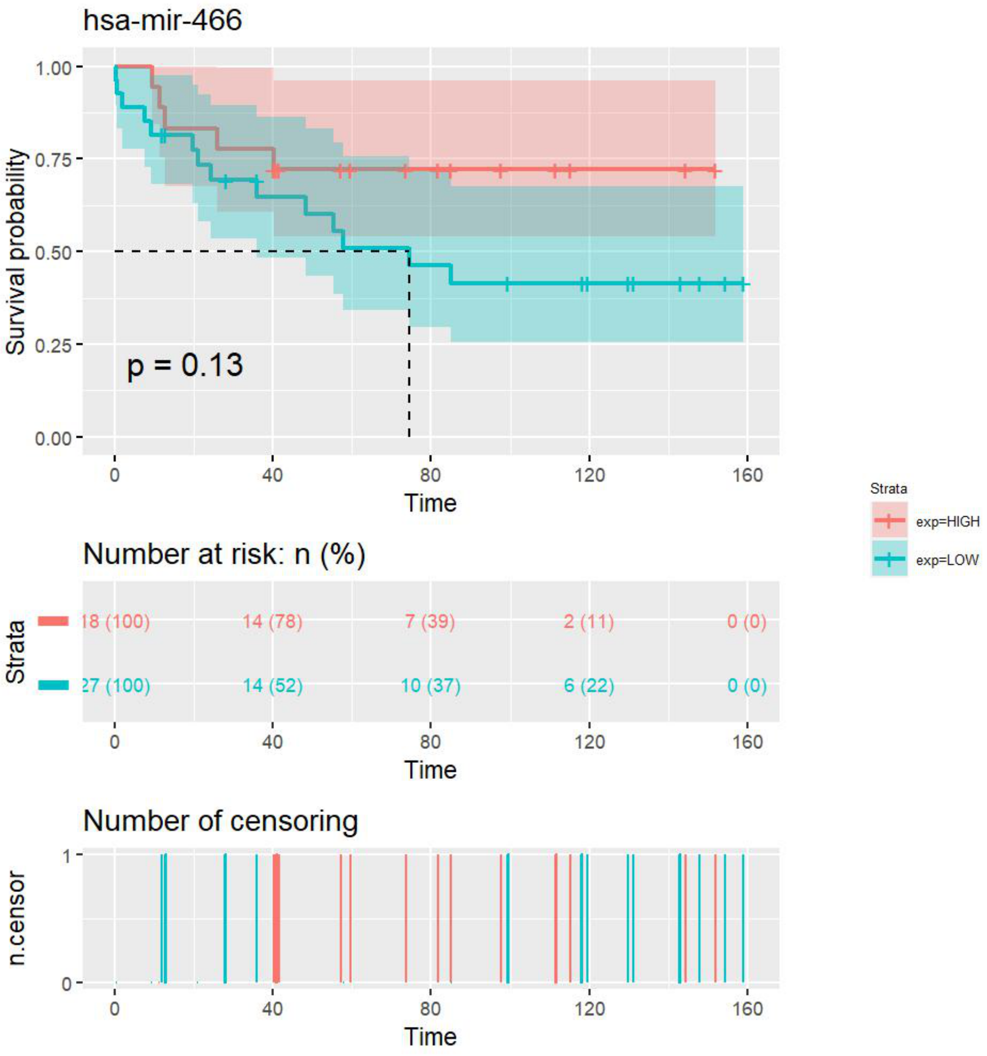
hsa-mir-466
Survival probability
1.00
0.75
0.50
0.25
0.00
p = 0.13
0
40
80
120
160
Time
Strata
exp=HIGH
exp=LOW
Number at risk: n (%)
Strata
18 (100)
14 (78)
7 (39)
2 (11)
0 (0)
27 (100)
14 (52)
10 (37)
6 (22)
0 (0)
Number of censoring
n.censor
1
0

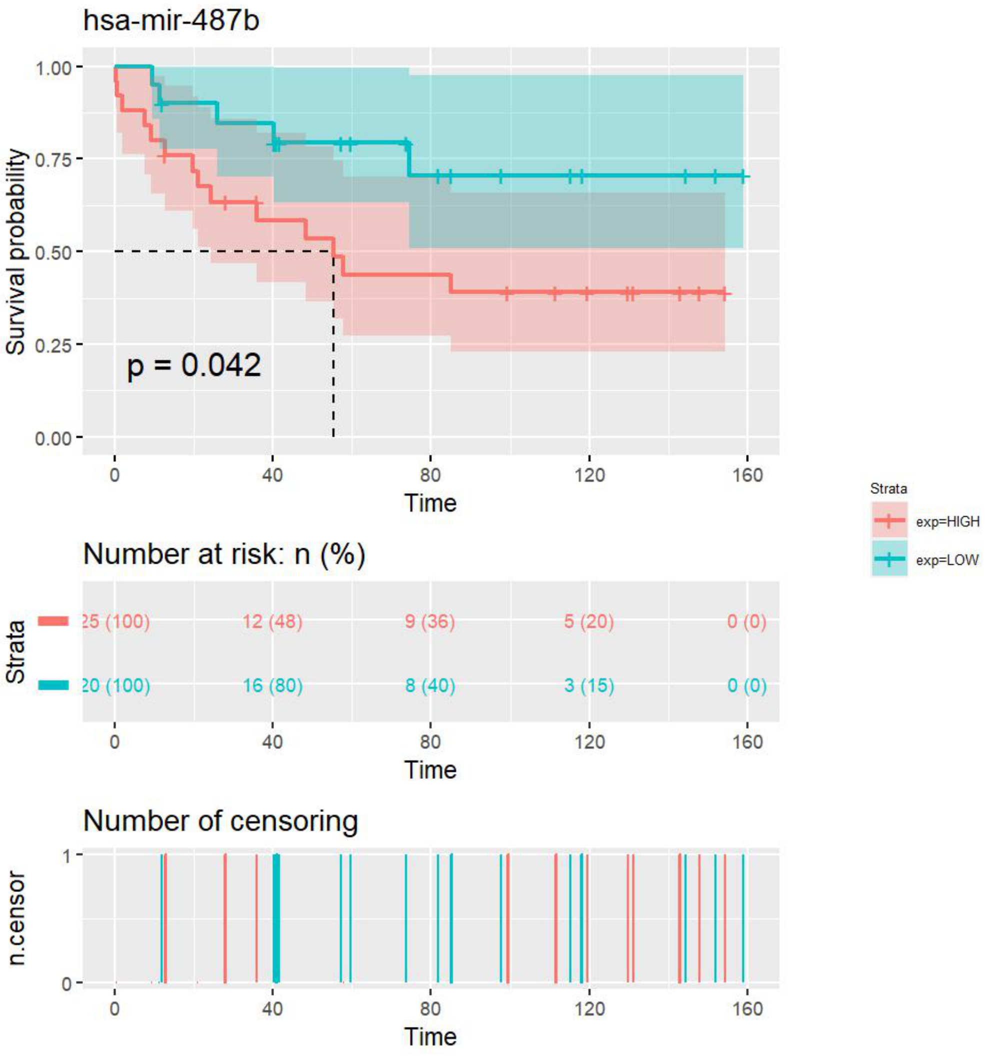
hsa-mir-487b
Survival probability
1.00
0.75
0.50
0.25
0.00
p = 0.042
0
40
80
120
160
Time
Strata
exp=HIGH
exp=LOW
Number at risk: n (%)
Strata
25 (100)
12 (48)
9 (36)
5 (20)
0 (0)
20 (100)
16 (80)
8 (40)
3 (15)
0 (0)
Time
Number of censoring
n.censor
1
0
Time

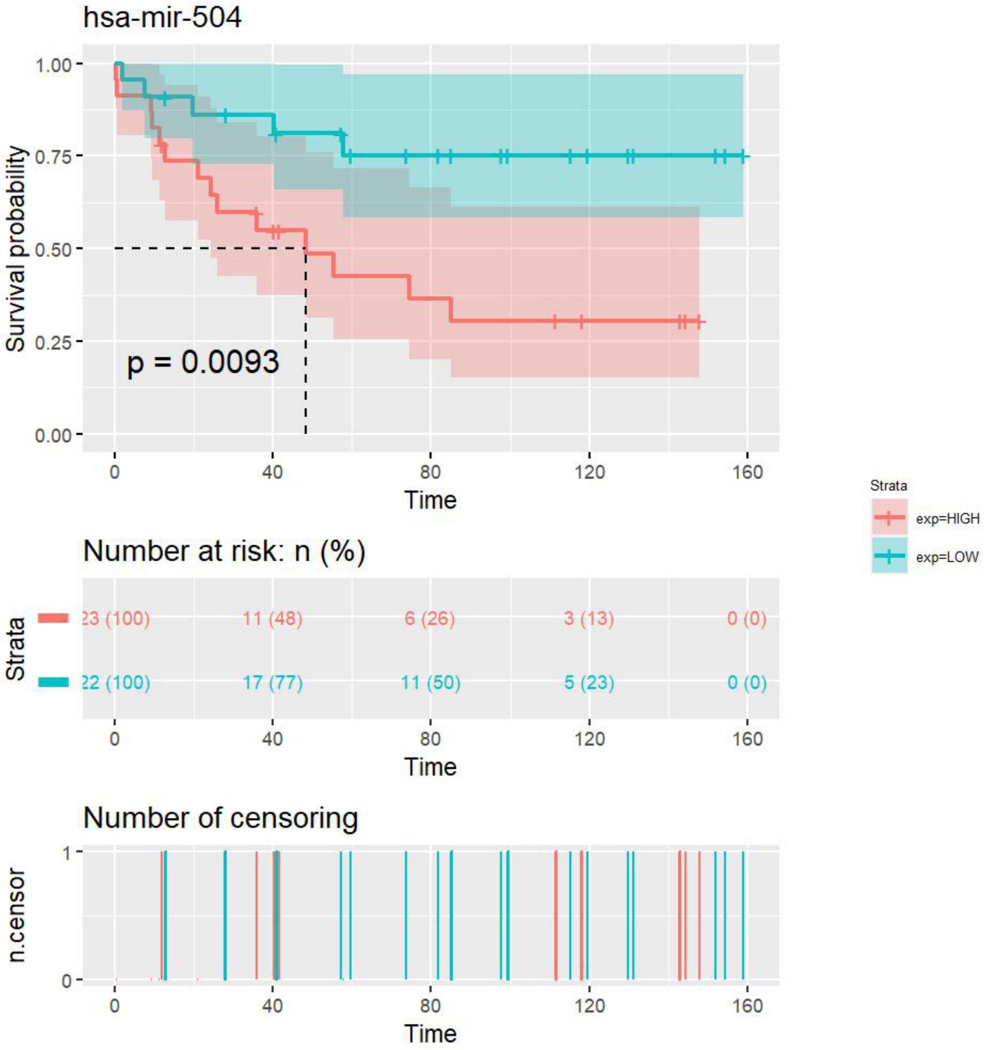

hsa-mir-504
1.00
0.75
0.50
0.25
0.00
Survival probability
p = 0.0093
0
40
80
120
160
Time
Strata
exp=HIGH
exp=LOW
Number at risk: n (%)
Strata
23 (100)
11 (48)
6 (26)
3 (13)
0 (0)
22 (100)
17 (77)
11 (50)
5 (23)
0 (0)
Time
Number of censoring
n.censor
1
0
Time

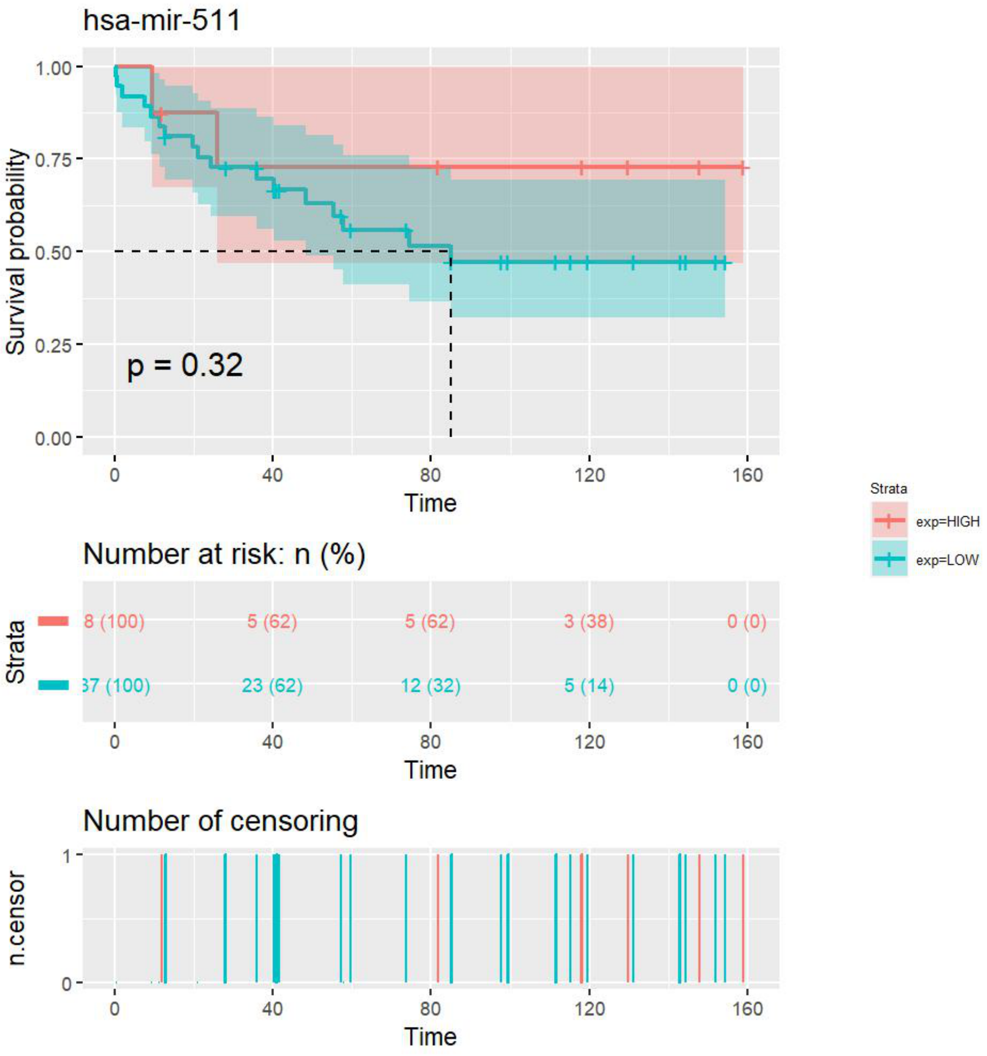

hsa-mir-511
Survival probability
1.00
0.75
0.50
0.25
0.00
p = 0.32
0
40
80
120
160
Time
Strata
exp=HIGH
exp=LOW
Number at risk: n (%)
Strata
8 (100)
5 (62)
5 (62)
3 (38)
0 (0)
37 (100)
23 (62)
12 (32)
5 (14)
0 (0)
Time
Number of censoring
n.censor
1
0
Time

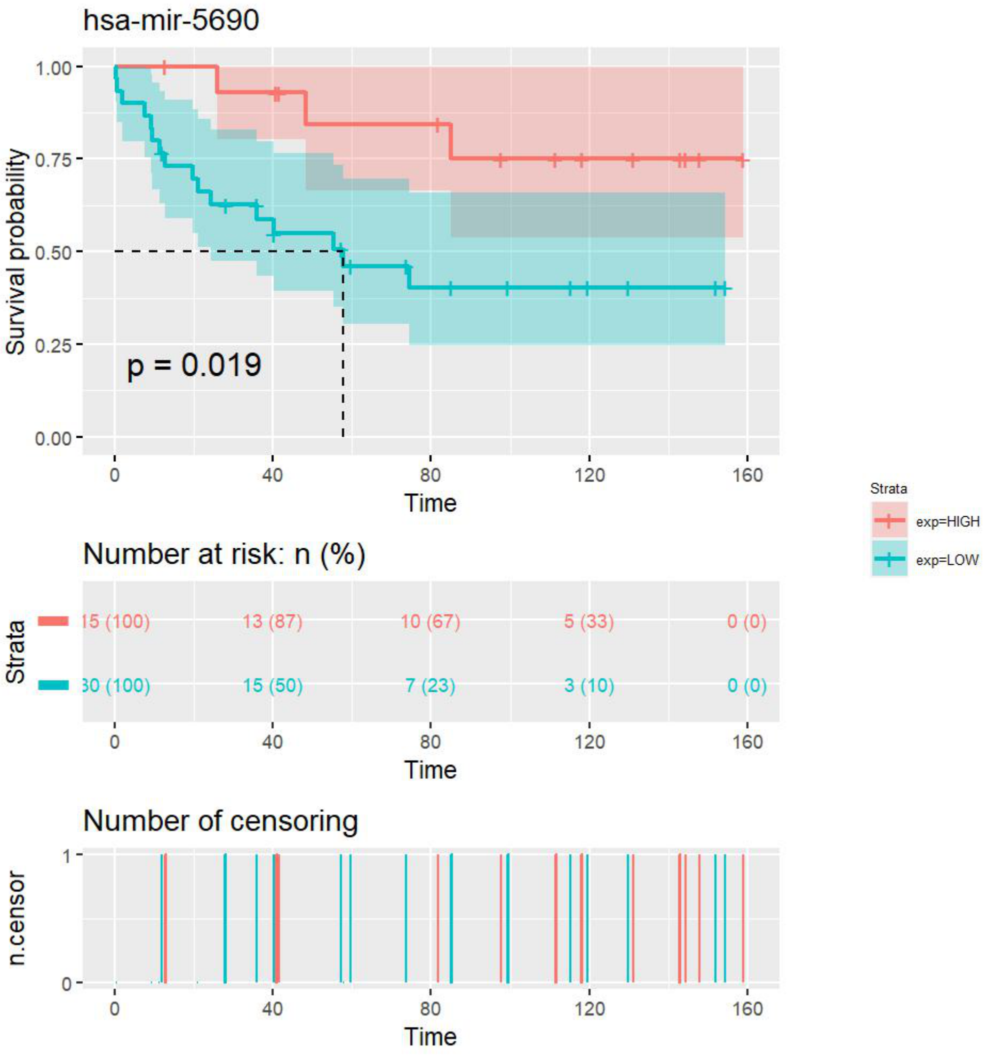

hsa-mir-5690
Survival probability
1.00
0.75
0.50
0.25
0.00
p = 0.019
0
40
80
120
160
Time
Strata
exp=HIGH
exp=LOW
Number at risk: n (%)
Strata
15 (100)
13 (87)
10 (67)
5 (33)
0 (0)
30 (100)
15 (50)
7 (23)
3 (10)
0 (0)
Time
Number of censoring
n.censor
1
0
Time

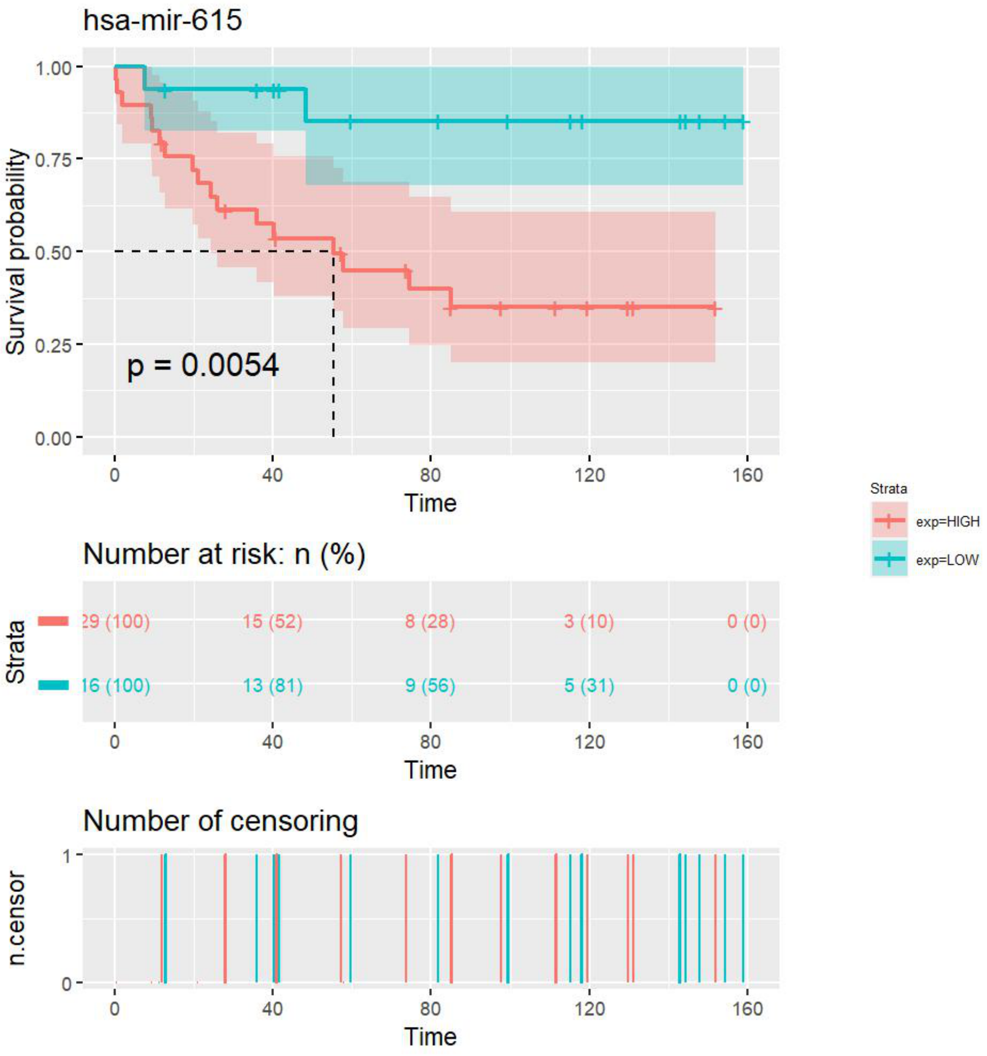

hsa-mir-615
Survival probability
1.00
0.75
0.50
0.25
0.00
p = 0.0054
0
40
80
120
160
Time
Strata
exp=HIGH
exp=LOW
Number at risk: n (%)
Strata
29 (100)
15 (52)
8 (28)
3 (10)
0 (0)
16 (100)
13 (81)
9 (56)
5 (31)
0 (0)
Time
Number of censoring
n.censor
1
0
Time

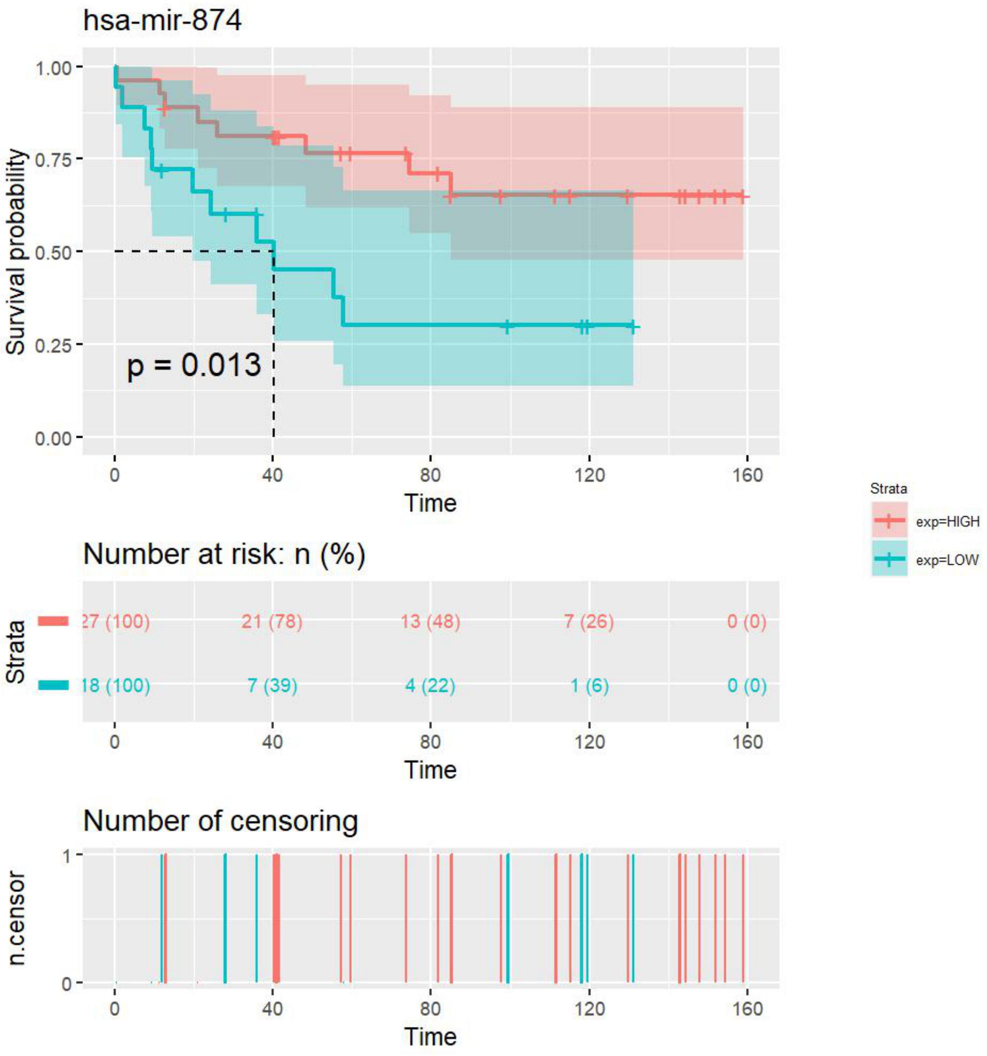

hsa-mir-874
Survival probability
1.00
0.75
0.50
0.25
0.00
p = 0.013
0
40
80
120
160
Time
Strata
exp=HIGH
exp=LOW
Number at risk: n (%)
Strata
27 (100)
21 (78)
13 (48)
7 (26)
0 (0)
18 (100)
7 (39)
4 (22)
1 (6)
0 (0)
Time
Number of censoring
n.censor
1
0
Time
