# Supplementary material for: Machine learning combining multi-omics data and network algorithms identifies adrenocortical carcinoma prognostic biomarkers
Source: Front Mol Biosci. 2023 Nov 6;10:1258902. doi: 10.3389/fmolb.2023.1258902 (PMC10658191; doi:10.3389/fmolb.2023.1258902)
Supplement: Supplementary file 6 [file DataSheet1.PDF]

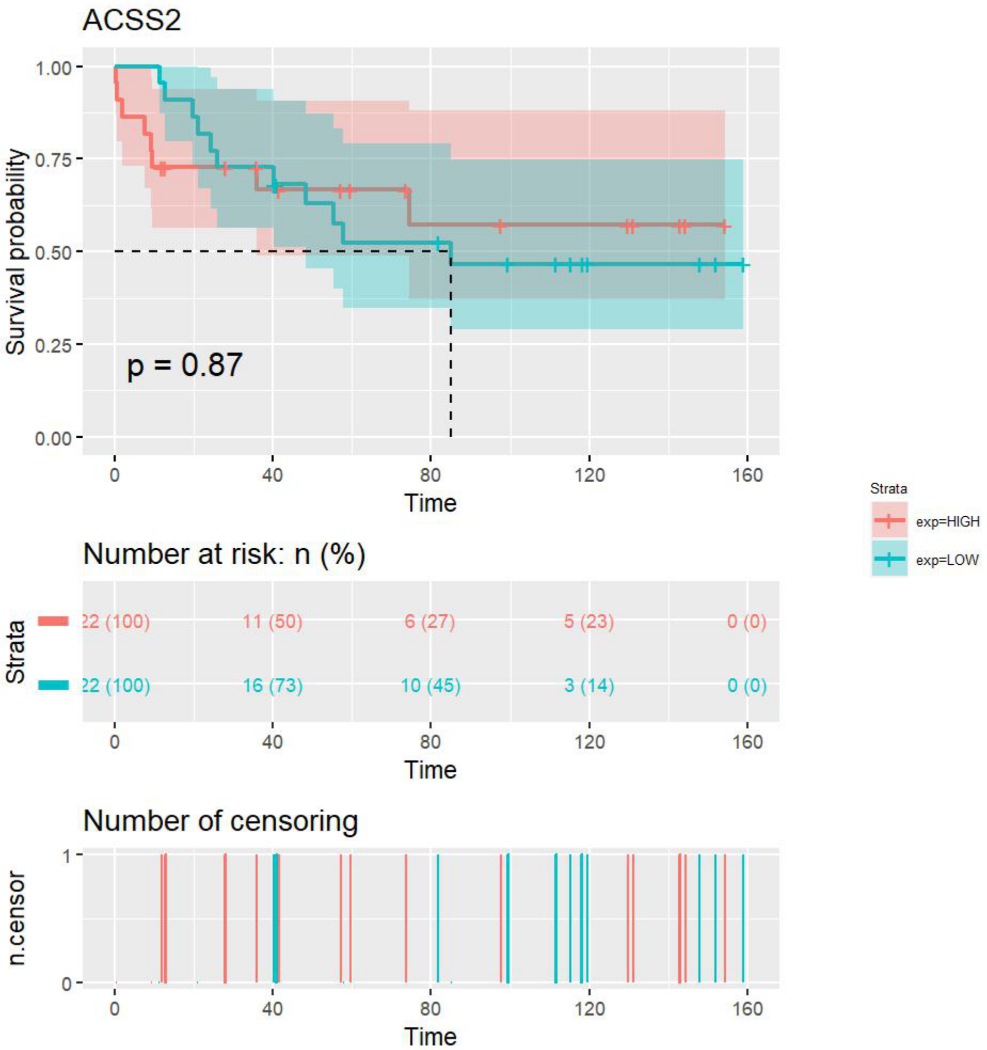
ACSS2
Survival probability
1.00
0.75
0.50
0.25
0.00
p = 0.87
0
40
80
120
160
Time
Strata
exp=HIGH
exp=LOW
Number at risk: n (%)
Strata
22 (100)
11 (50)
6 (27)
5 (23)
0 (0)
22 (100)
16 (73)
10 (45)
3 (14)
0 (0)
Number of censoring
n.censor
1
0

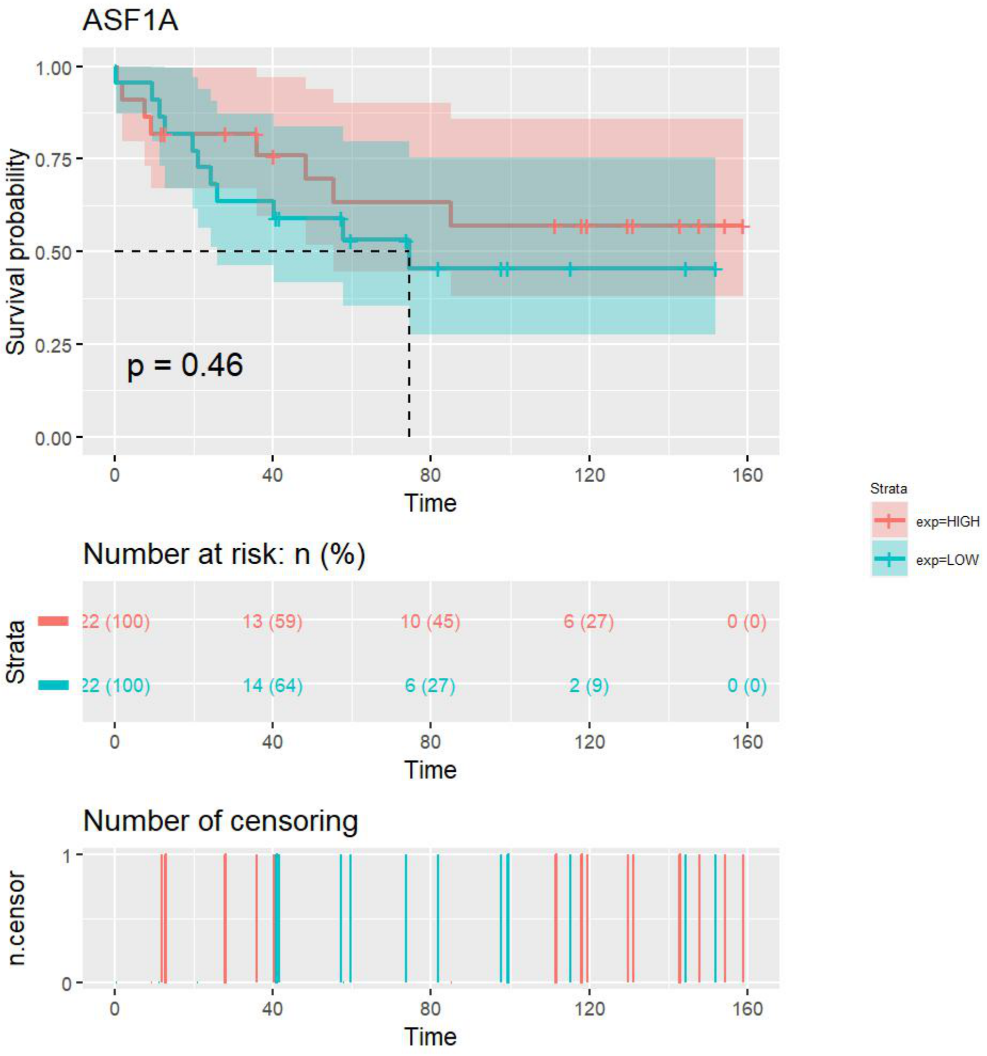
ASF1A
Survival probability
1.00
0.75
0.50
0.25
0.00
p = 0.46
0
40
80
120
160
Time
Strata
exp=HIGH
exp=LOW
Number at risk: n (%)
Strata
22 (100)
13 (59)
10 (45)
6 (27)
0 (0)
22 (100)
14 (64)
6 (27)
2 (9)
0 (0)
Time
Number of censoring
n.censor
1
0
Time

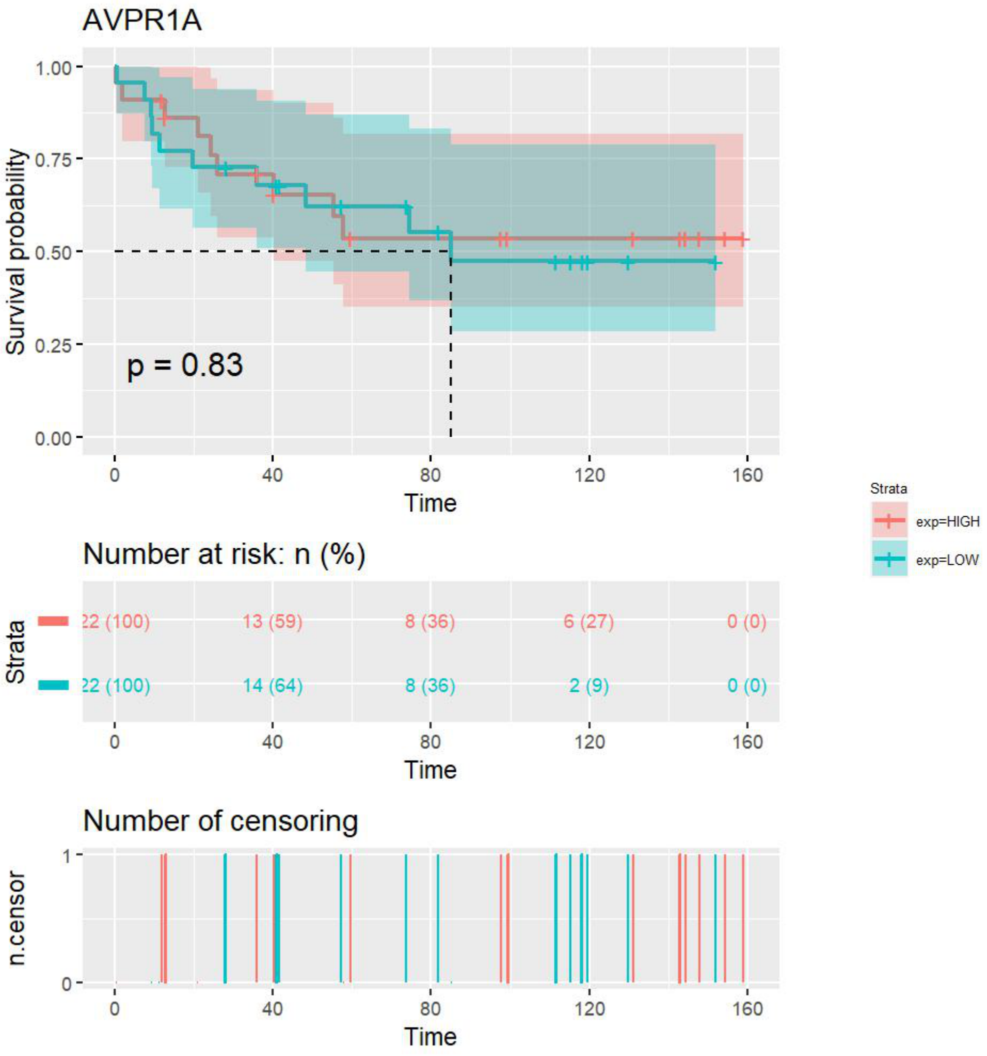
AVPR1A
Survival probability
1.00
0.75
0.50
0.25
0.00
p = 0.83
0
40
80
120
160
Time
Strata
exp=HIGH
exp=LOW
Number at risk: n (%)
Strata
22 (100)
13 (59)
8 (36)
6 (27)
0 (0)
22 (100)
14 (64)
8 (36)
2 (9)
0 (0)
Number of censoring
n.censor
1
0

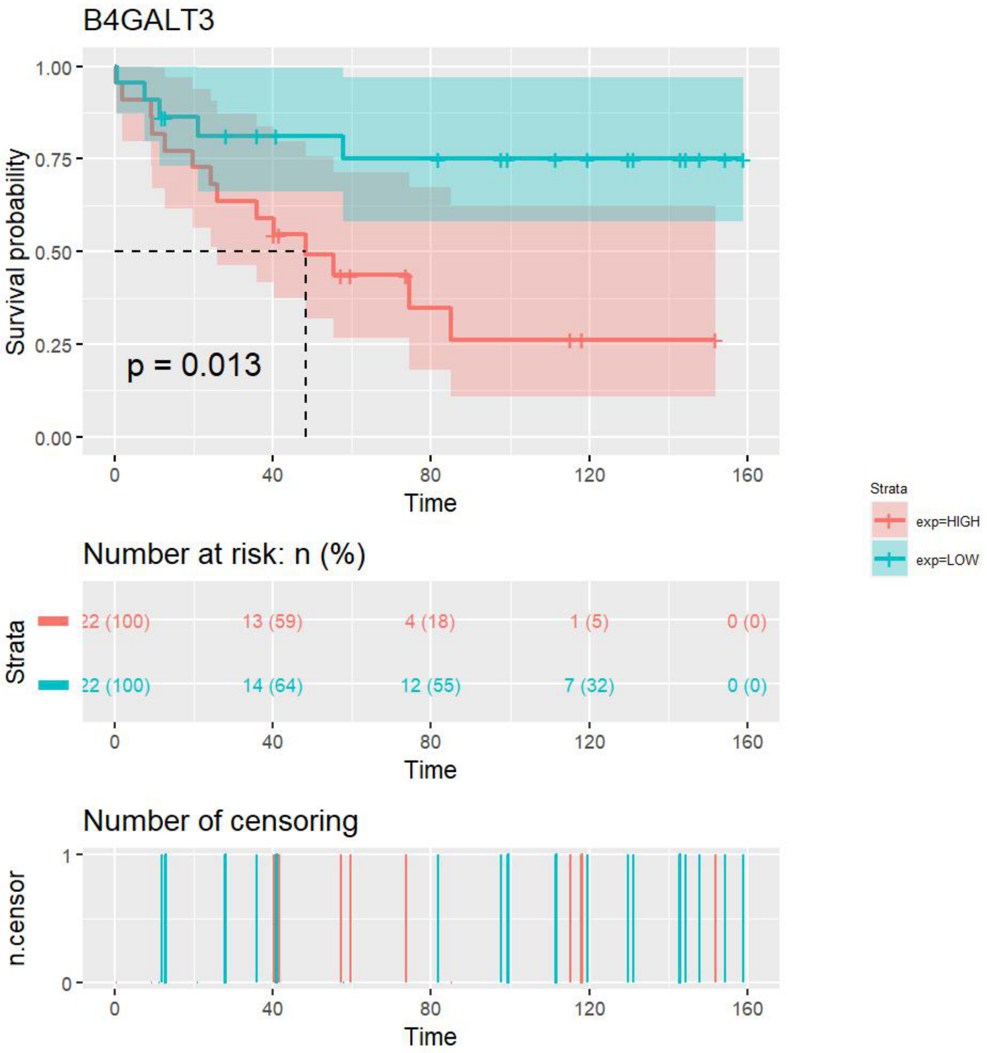

B4GALT3
Survival probability
1.00
0.75
0.50
0.25
0.00
p = 0.013
0
40
80
120
160
Time
Strata
exp=HIGH
exp=LOW
Number at risk: n (%)
Strata
22 (100)
13 (59)
4 (18)
1 (5)
0 (0)
22 (100)
14 (64)
12 (55)
7 (32)
0 (0)
Time
Number of censoring
n.censor
1
0
Time

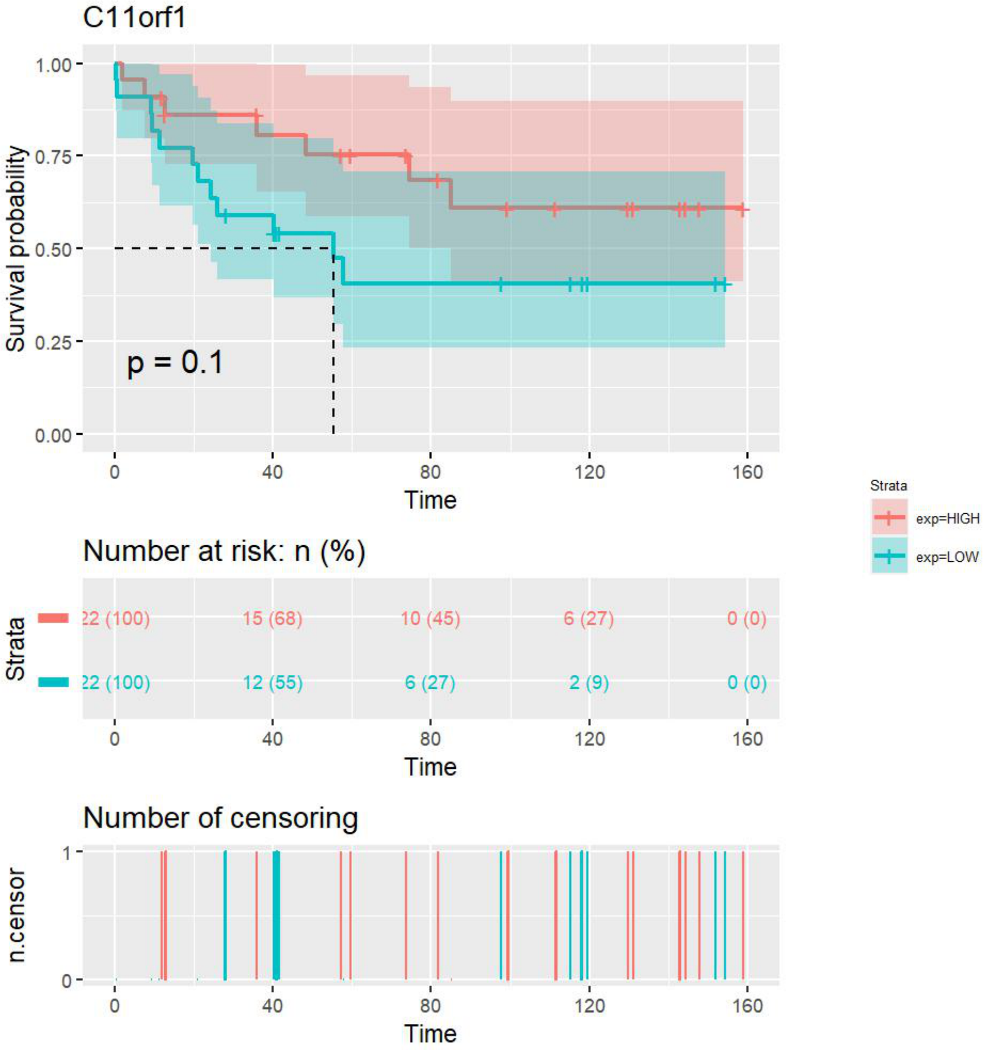

C11orf1
Survival probability
1.00
0.75
0.50
0.25
0.00
p = 0.1
0
40
80
120
160
Time
Strata
exp=HIGH
exp=LOW
Number at risk: n (%)
Strata
22 (100)
15 (68)
10 (45)
6 (27)
0 (0)
22 (100)
12 (55)
6 (27)
2 (9)
0 (0)
Time
Number of censoring
n.censor
1
0
Time

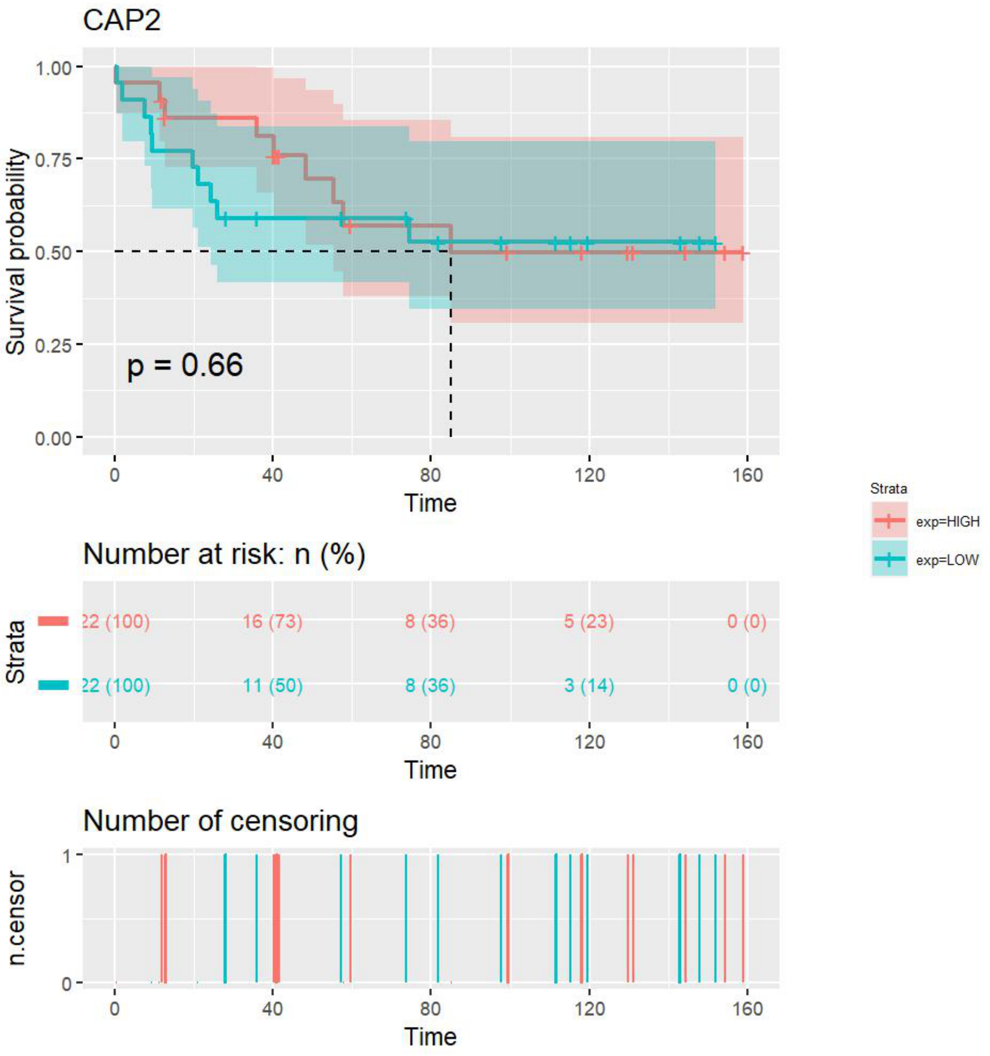

CAP2
Survival probability
1.00
0.75
0.50
0.25
0.00
p = 0.66
0
40
80
120
160
Time
Strata
exp=HIGH
exp=LOW
Number at risk: n (%)
Strata
22 (100) 16 (73) 8 (36) 5 (23) 0 (0)
22 (100) 11 (50) 8 (36) 3 (14) 0 (0)
Time
Number of censoring
n.censor
1
0
Time

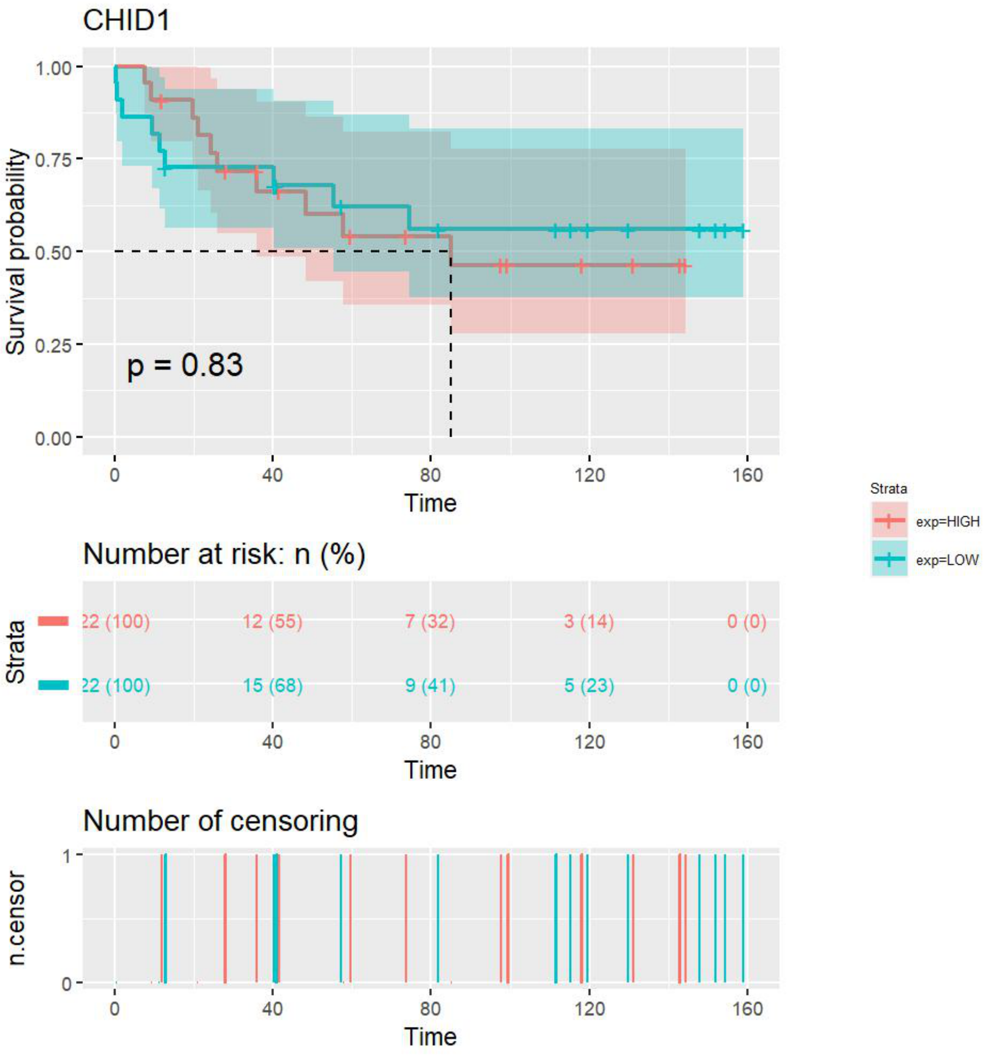

CHID1
Survival probability
1.00
0.75
0.50
0.25
0.00
p = 0.83
0
40
80
120
160
Time
Strata
exp=HIGH
exp=LOW
Number at risk: n (%)
Strata
22 (100)
12 (55)
7 (32)
3 (14)
0 (0)
22 (100)
15 (68)
9 (41)
5 (23)
0 (0)
Time
Number of censoring
n.censor
1
0
Time

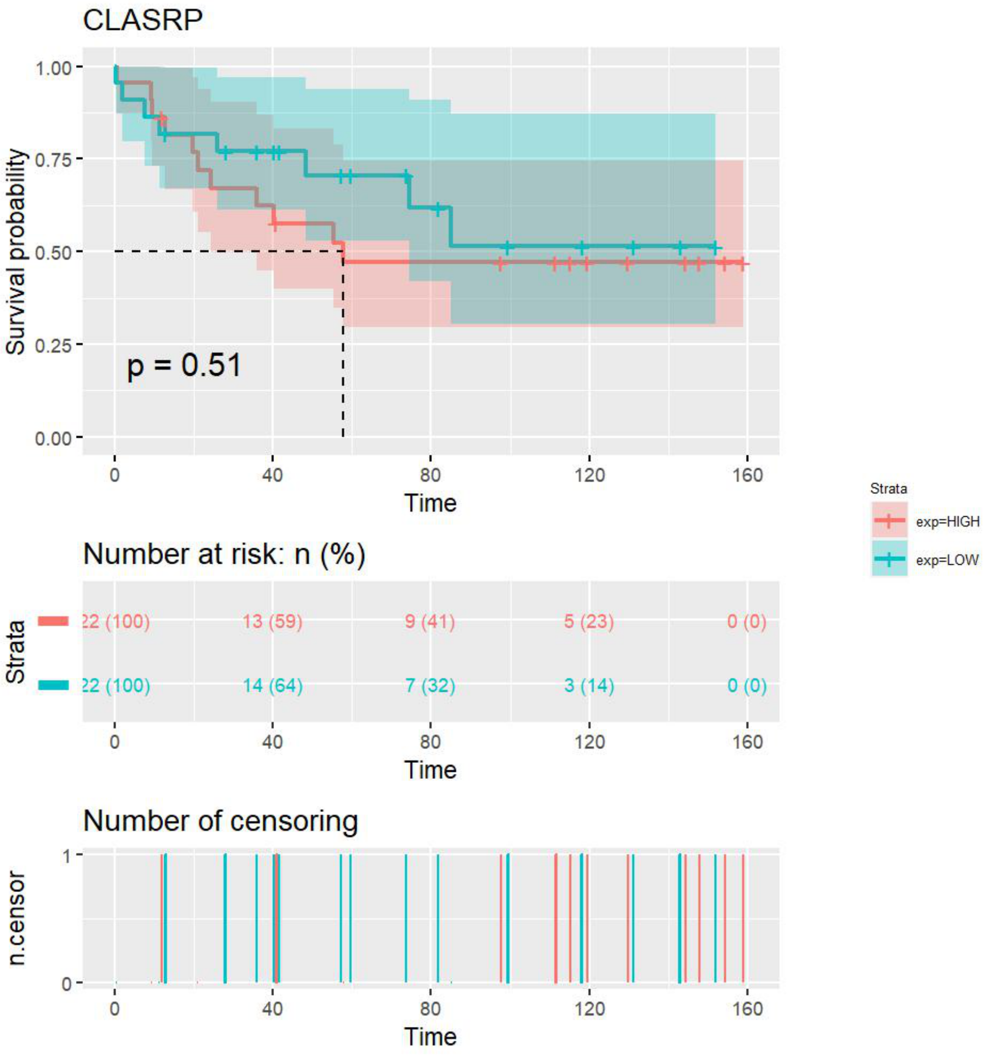

CLASRP
Survival probability
1.00
0.75
0.50
0.25
0.00
p = 0.51
0
40
80
120
160
Time
Strata
exp=HIGH
exp=LOW
Number at risk: n (%)
Strata
22 (100)
13 (59)
9 (41)
5 (23)
0 (0)
22 (100)
14 (64)
7 (32)
3 (14)
0 (0)
Time
Number of censoring
n.censor
1
0
Time

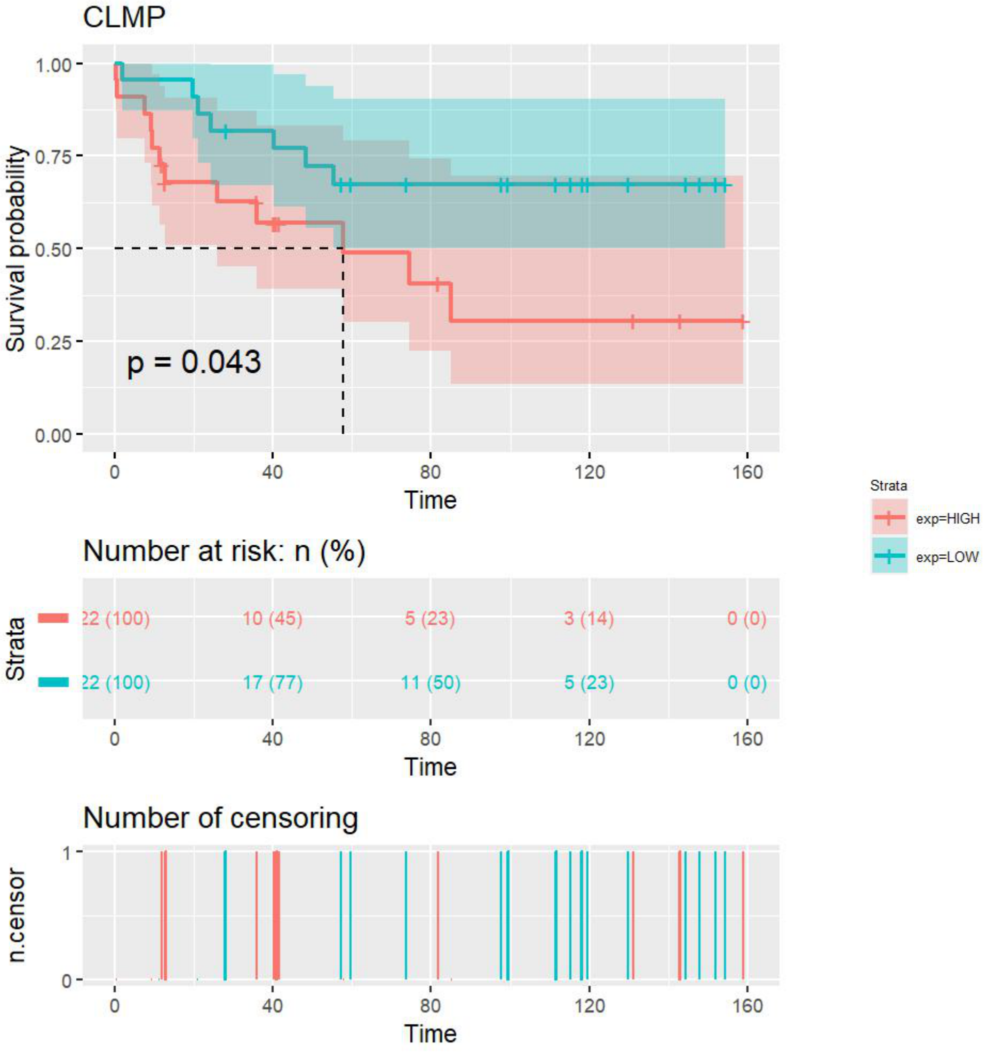

CLMP
Survival probability
1.00
0.75
0.50
0.25
0.00
p = 0.043
0
40
80
120
160
Time
Strata
exp=HIGH
exp=LOW
Number at risk: n (%)
Strata
22 (100)
10 (45)
5 (23)
3 (14)
0 (0)
22 (100)
17 (77)
11 (50)
5 (23)
0 (0)
Time
Number of censoring
n.censor
1
0
Time

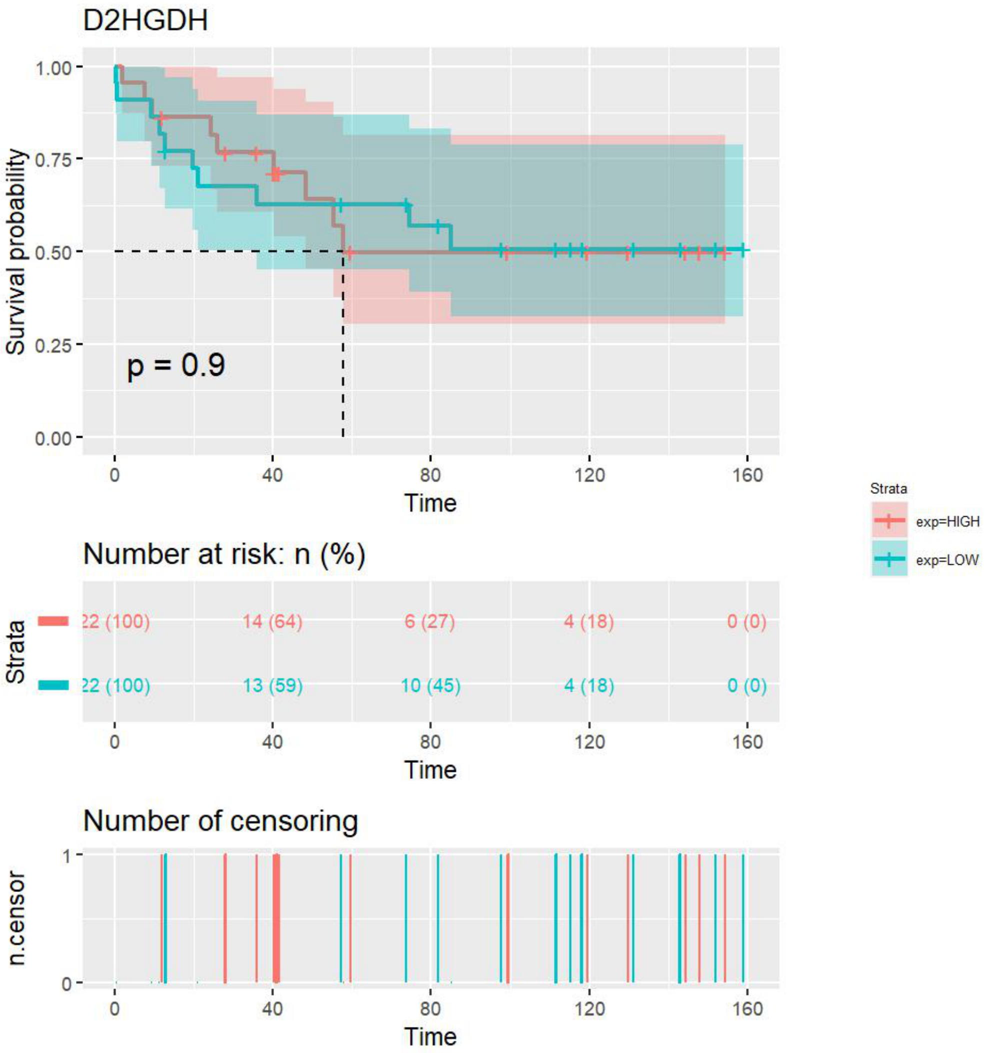

D2HGDH
Survival probability
1.00
0.75
0.50
0.25
0.00
p = 0.9
0
40
80
120
160
Time
Strata
exp=HIGH
exp=LOW
Number at risk: n (%)
Strata
22 (100)
14 (64)
6 (27)
4 (18)
0 (0)
22 (100)
13 (59)
10 (45)
4 (18)
0 (0)
Time
Number of censoring
n.censor
1
0
Time

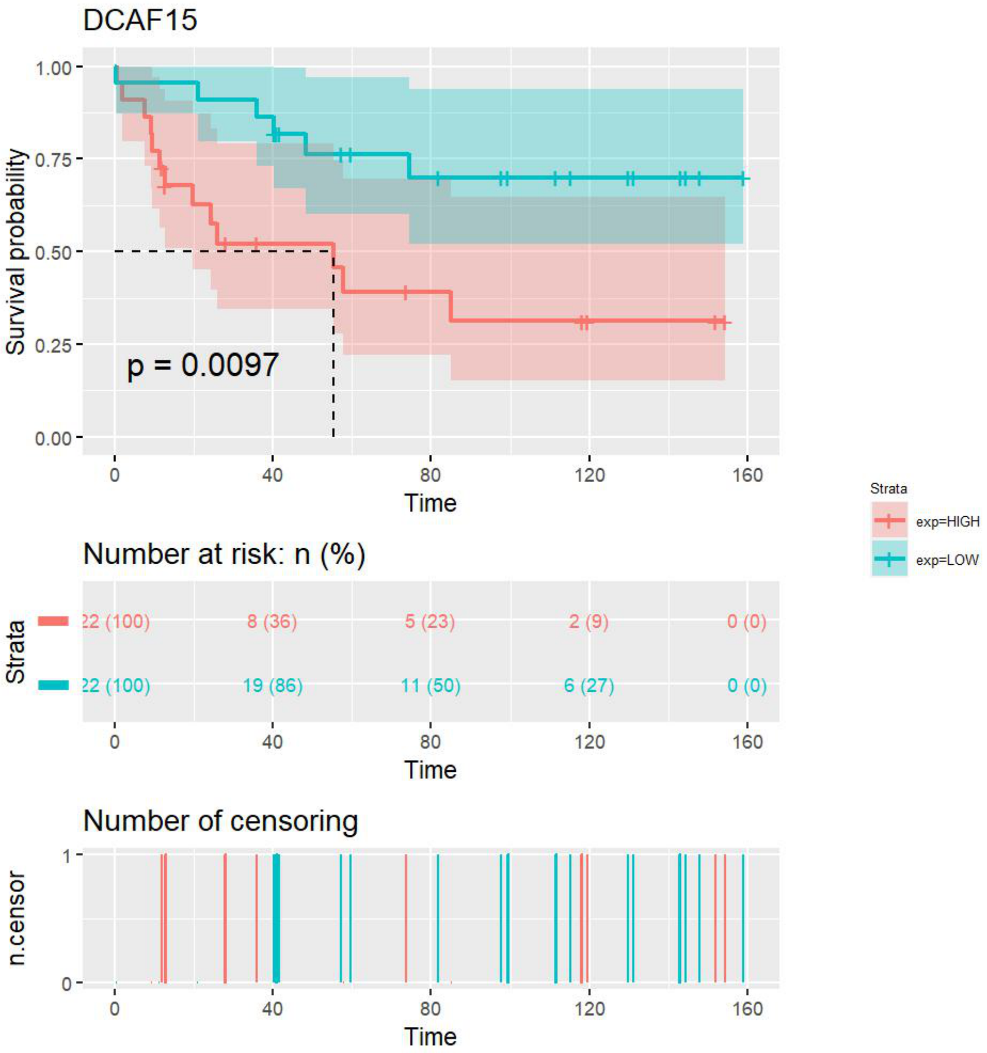

DCAF15
Survival probability
1.00
0.75
0.50
0.25
0.00
p = 0.0097
0
40
80
120
160
Time
Strata
exp=HIGH
exp=LOW
Number at risk: n (%)
Strata
22 (100)
8 (36)
5 (23)
2 (9)
0 (0)
22 (100)
19 (86)
11 (50)
6 (27)
0 (0)
Time
Number of censoring
n.censor
1
0
Time

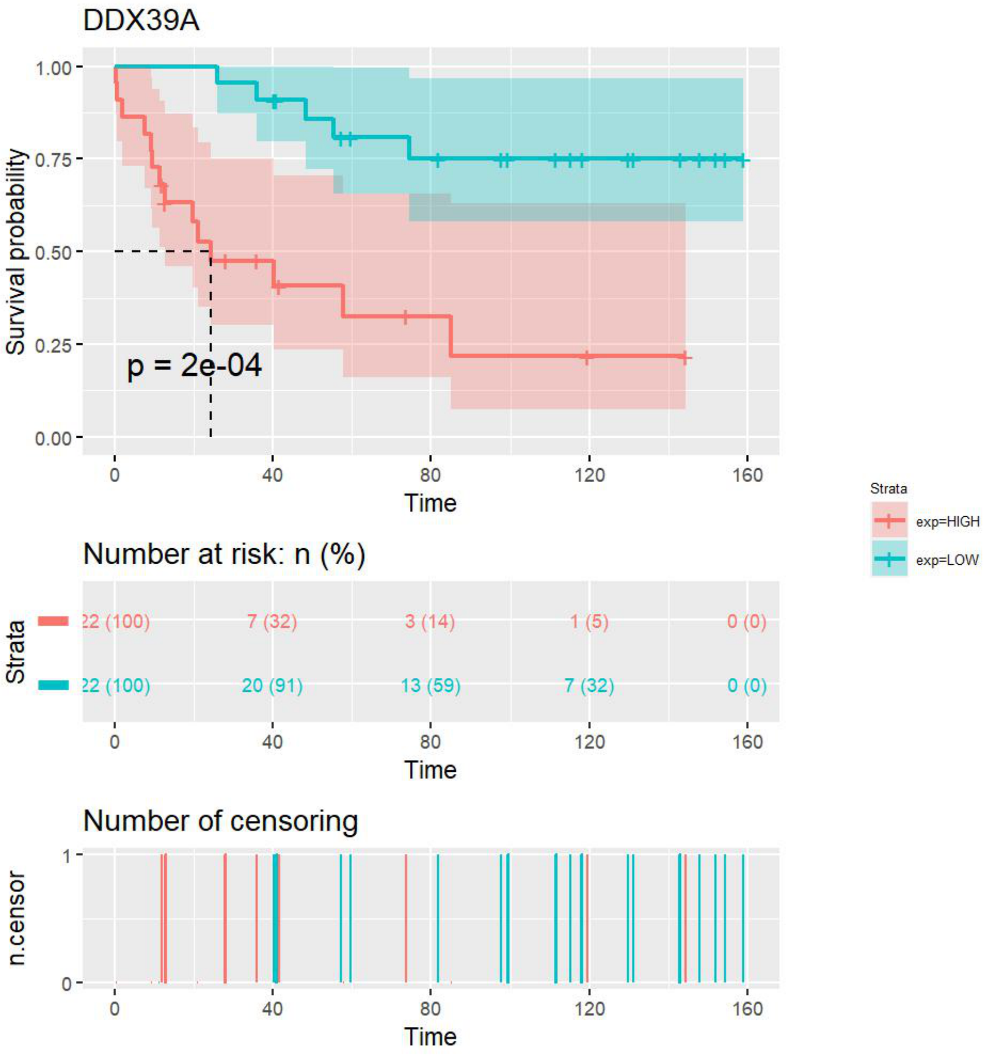

DDX39A
Survival probability
1.00
0.75
0.50
0.25
0.00
p = 2e-04
0
40
80
120
160
Time
Strata
exp=HIGH
exp=LOW
Number at risk: n (%)
Strata
22 (100)
7 (32)
3 (14)
1 (5)
0 (0)
22 (100)
20 (91)
13 (59)
7 (32)
0 (0)
Time
Number of censoring
n.censor
1
0
Time

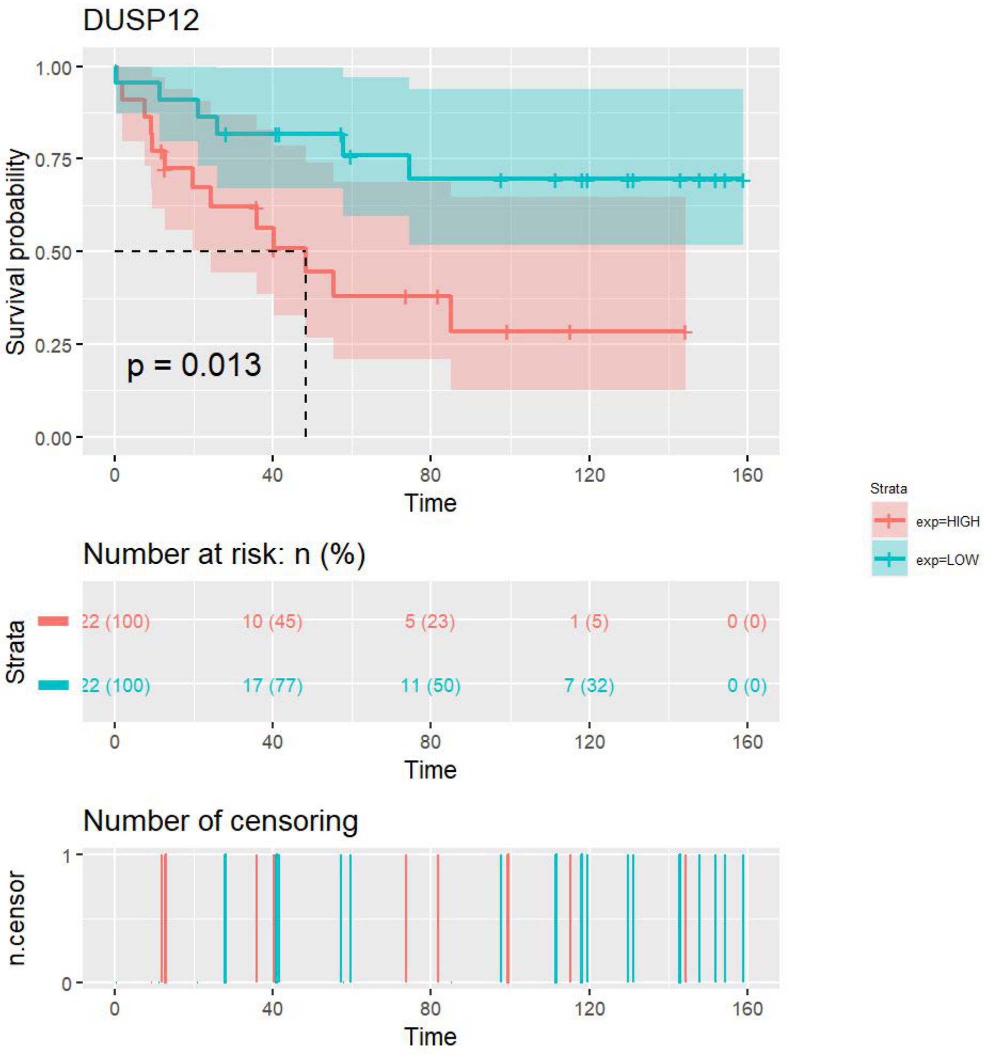

DUSP12
Survival probability
1.00
0.75
0.50
0.25
0.00
p = 0.013
0
40
80
120
160
Time
Strata
exp=HIGH
exp=LOW
Number at risk: n (%)
Strata
22 (100)
10 (45)
5 (23)
1 (5)
0 (0)
22 (100)
17 (77)
11 (50)
7 (32)
0 (0)
Number of censoring
n.censor
1
0

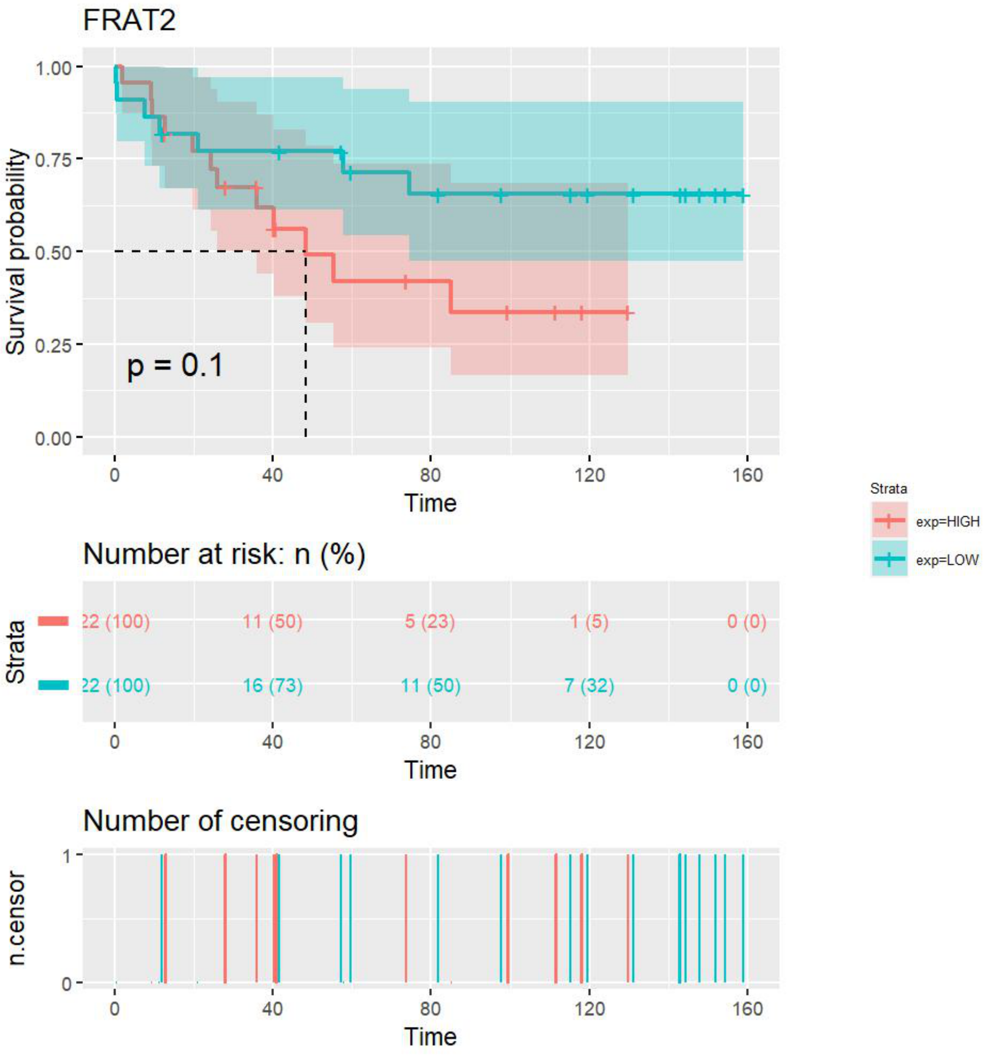
FRAT2
Survival probability
1.00
0.75
0.50
0.25
0.00
p = 0.1
0
40
80
120
160
Time
Strata
exp=HIGH
exp=LOW
Number at risk: n (%)
Strata
22 (100)
11 (50)
5 (23)
1 (5)
0 (0)
22 (100)
16 (73)
11 (50)
7 (32)
0 (0)
Time
Number of censoring
n.censor
1
0
Time

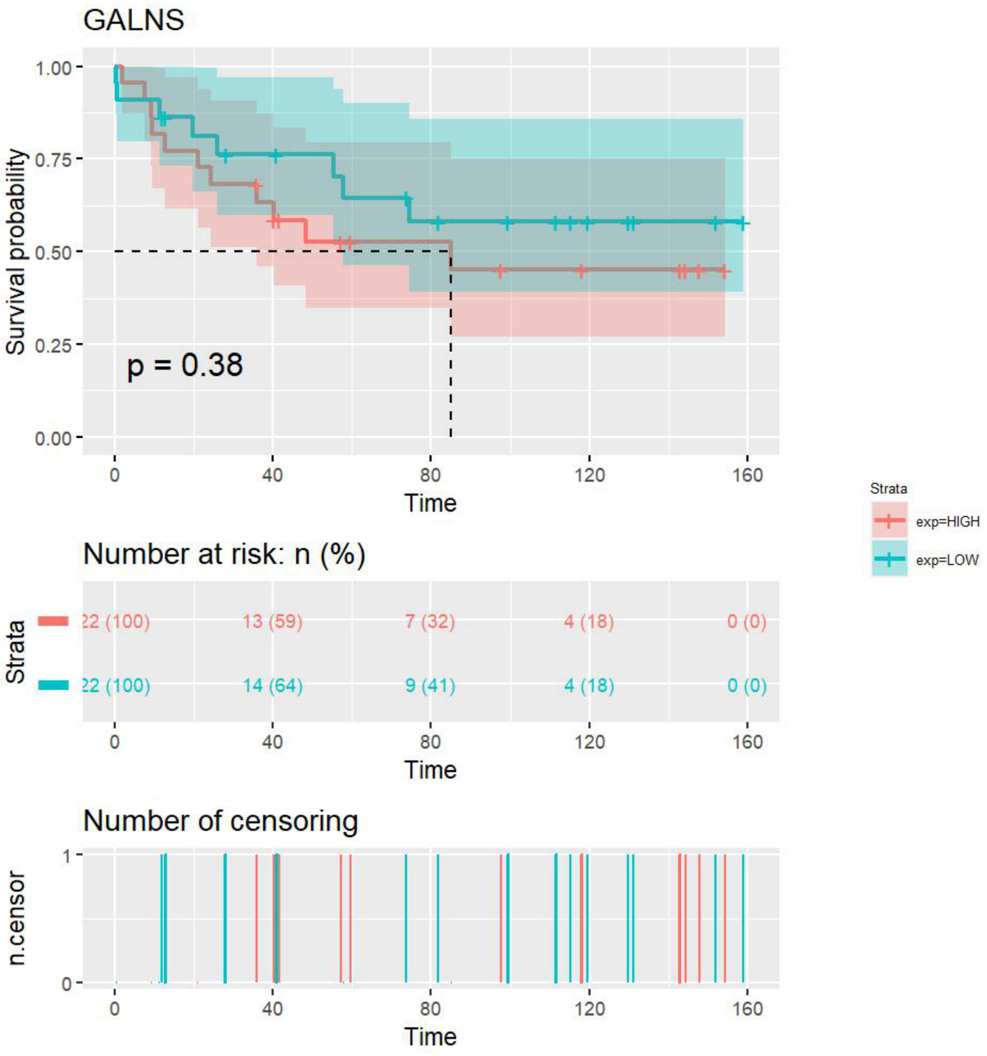
GALNS
Survival probability
1.00
0.75
0.50
0.25
0.00
p = 0.38
0
40
80
120
160
Time
Strata
exp=HIGH
exp=LOW
Number at risk: n (%)
22 (100)
13 (59)
7 (32)
4 (18)
0 (0)
22 (100)
14 (64)
9 (41)
4 (18)
0 (0)
Number of censoring
n.censor
1
0

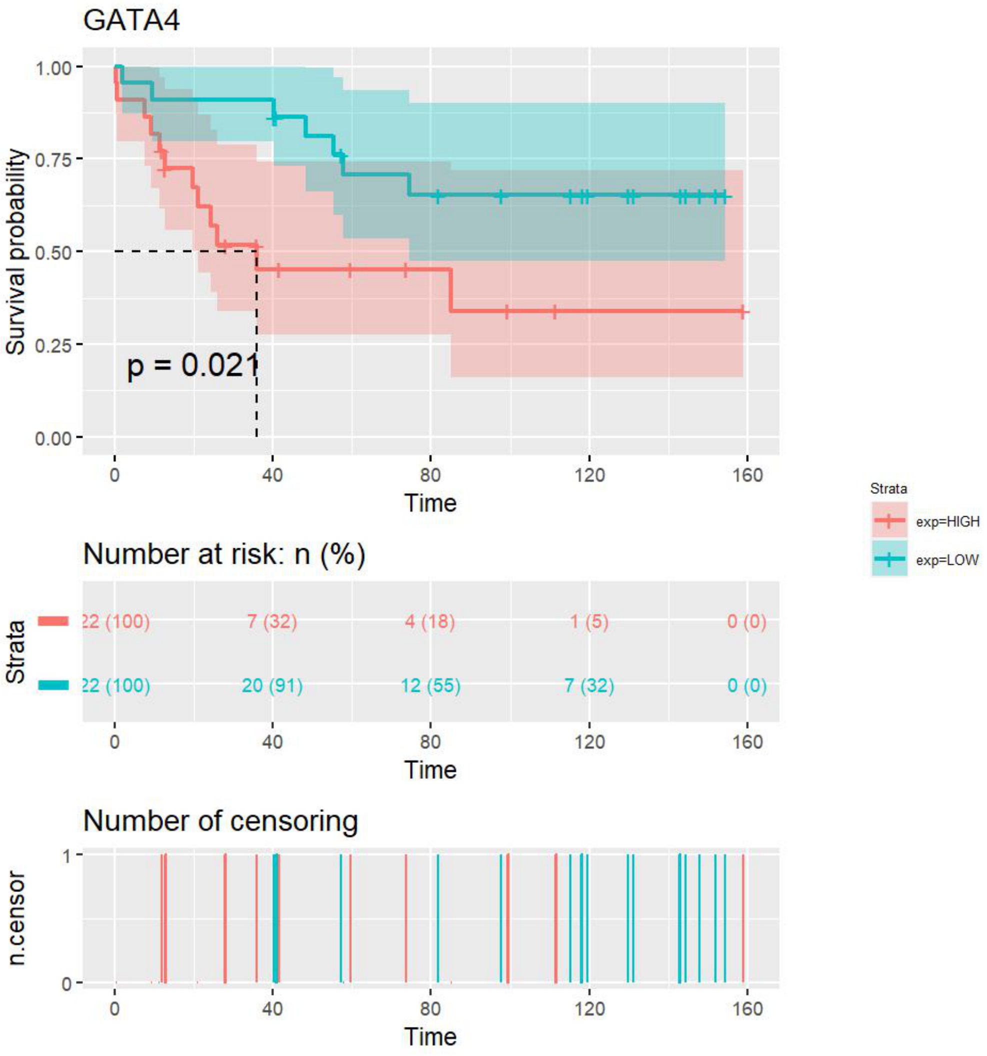
GATA4
Survival probability
1.00
0.75
0.50
0.25
0.00
p = 0.021
0
40
80
120
160
Time
Strata
exp=HIGH
exp=LOW
Number at risk: n (%)
Strata
22 (100)
7 (32)
4 (18)
1 (5)
0 (0)
22 (100)
20 (91)
12 (55)
7 (32)
0 (0)
0
40
80
120
160
Time
Number of censoring
n.censor
1
0
0
40
80
120
160
Time

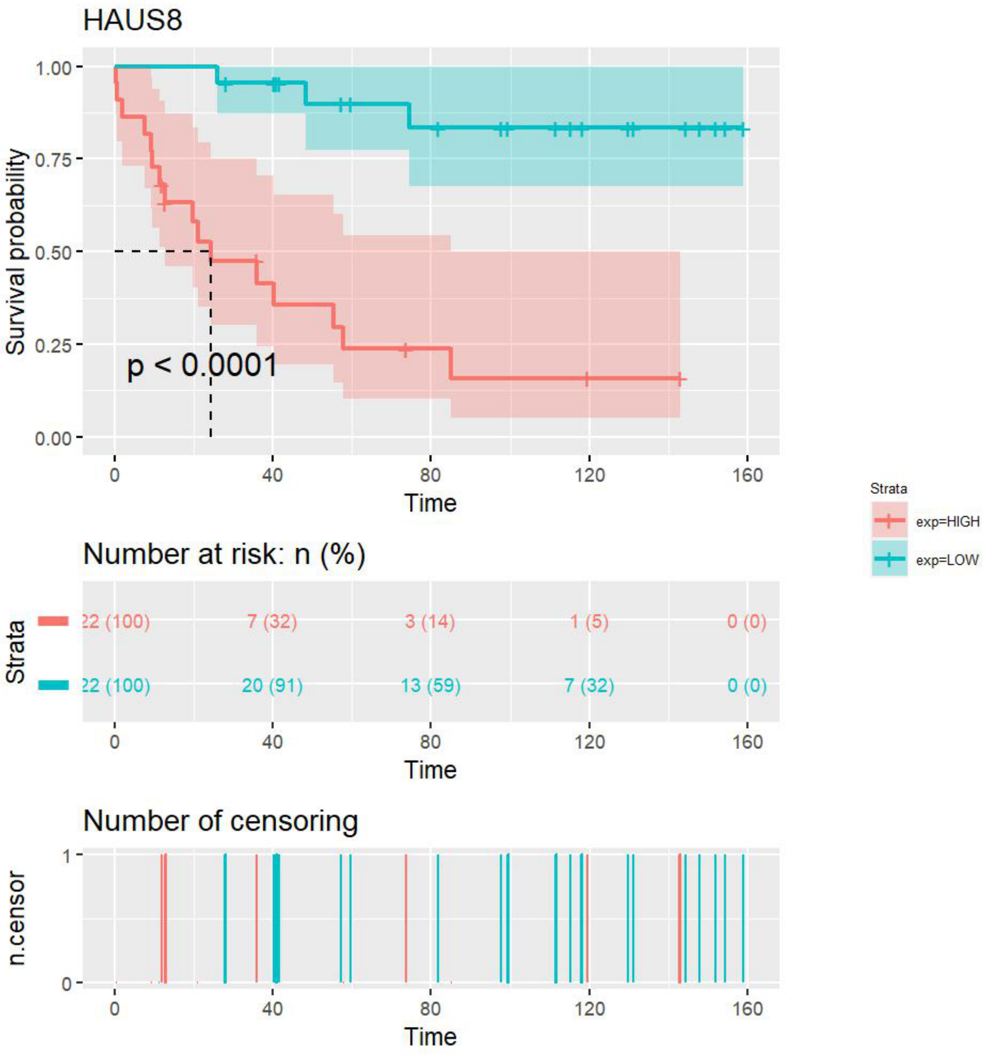
HAUS8
Survival probability
1.00
0.75
0.50
0.25
0.00
p < 0.0001
0
40
80
120
160
Time
Strata
exp=HIGH
exp=LOW
Number at risk: n (%)
Strata
22 (100)
7 (32)
3 (14)
1 (5)
0 (0)
22 (100)
20 (91)
13 (59)
7 (32)
0 (0)
Time
Number of censoring
n.censor
1
0
Time

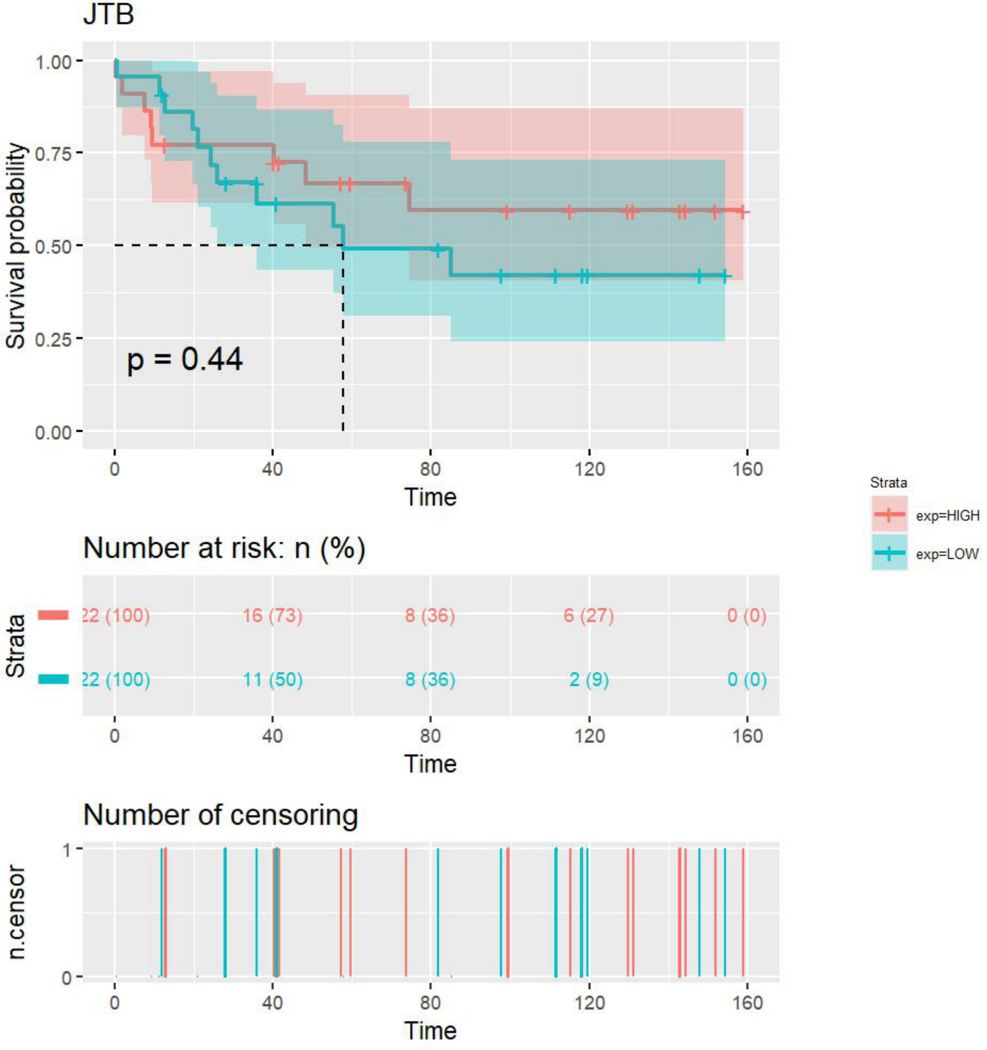

JTB
Survival probability
1.00
0.75
0.50
0.25
0.00
p = 0.44
0
40
80
120
160
Time
Strata
exp=HIGH
exp=LOW
Number at risk: n (%)
Strata
22 (100)
16 (73)
8 (36)
6 (27)
0 (0)
22 (100)
11 (50)
8 (36)
2 (9)
0 (0)
Time
Number of censoring
n.censor
1
0
Time

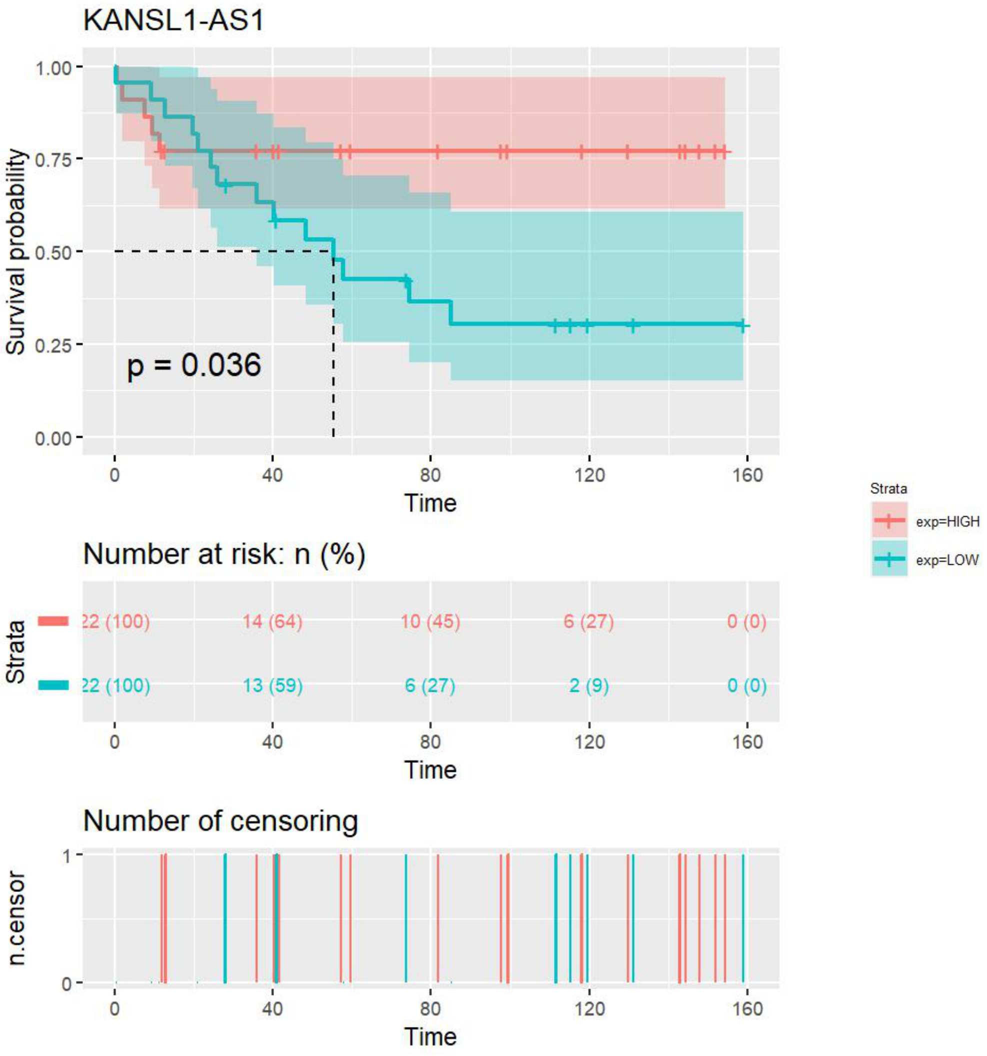

KANSL1-AS1
Survival probability
1.00
0.75
0.50
0.25
0.00
p = 0.036
0
40
80
120
160
Time
Strata
exp=HIGH
exp=LOW
Number at risk: n (%)
Strata
22 (100)
14 (64)
10 (45)
6 (27)
0 (0)
22 (100)
13 (59)
6 (27)
2 (9)
0 (0)
Time
Number of censoring
n.censor
1
0
Time

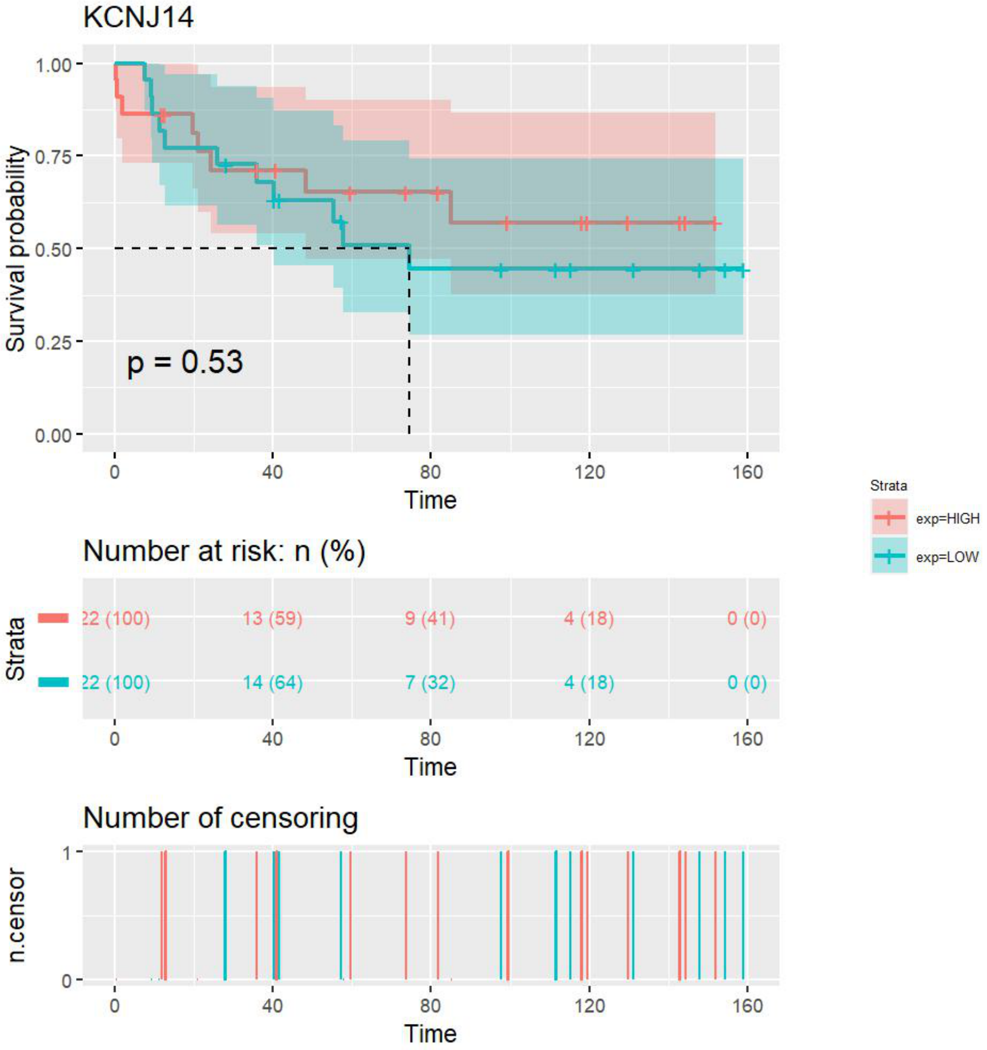
KCNJ14
Survival probability
1.00
0.75
0.50
0.25
0.00
p = 0.53
0
40
80
120
160
Time
Strata
exp=HIGH
exp=LOW
Number at risk: n (%)
22 (100)
13 (59)
9 (41)
4 (18)
0 (0)
22 (100)
14 (64)
7 (32)
4 (18)
0 (0)
Number of censoring
n.censor
1
0

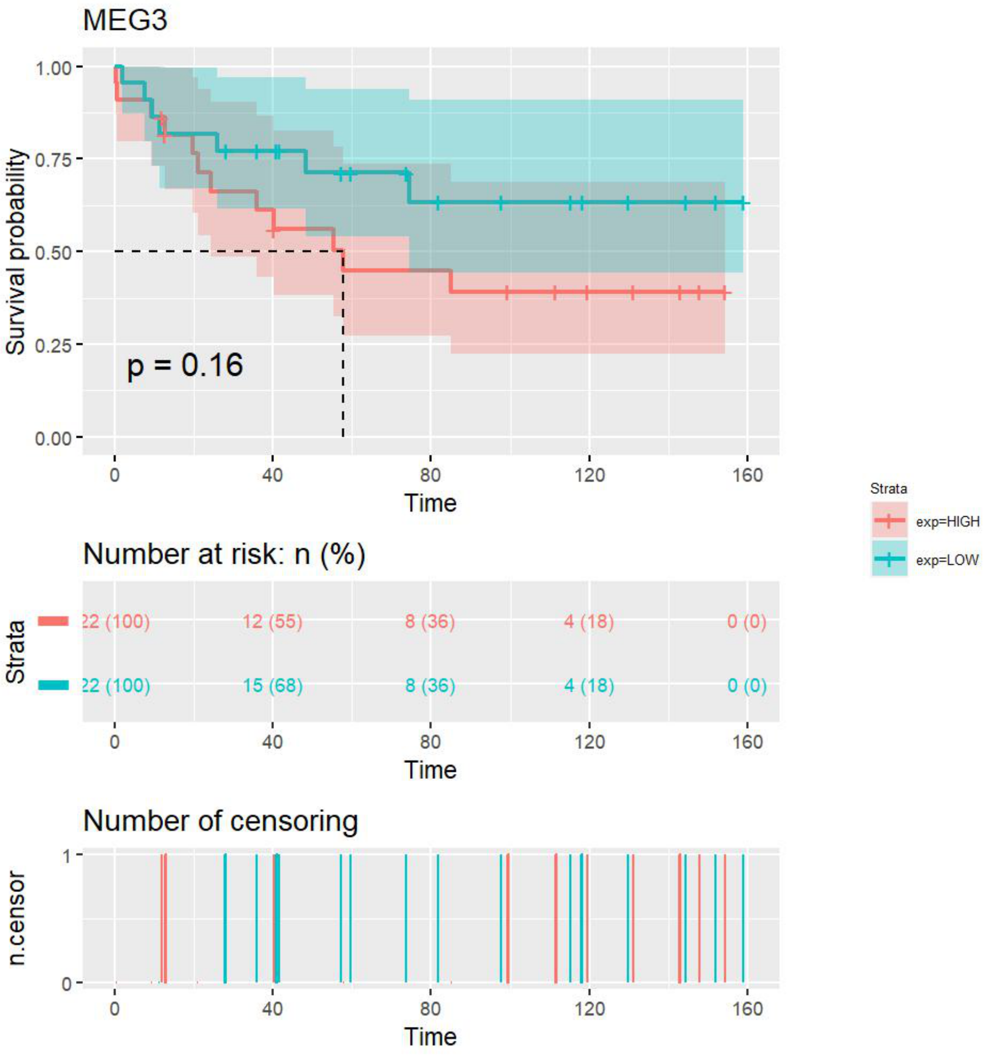

MEG3
Survival probability
1.00
0.75
0.50
0.25
0.00
p = 0.16
0
40
80
120
160
Time
Strata
exp=HIGH
exp=LOW
Number at risk: n (%)
Strata
22 (100)
12 (55)
8 (36)
4 (18)
0 (0)
22 (100)
15 (68)
8 (36)
4 (18)
0 (0)
Time
Number of censoring
n.censor
1
0
Time

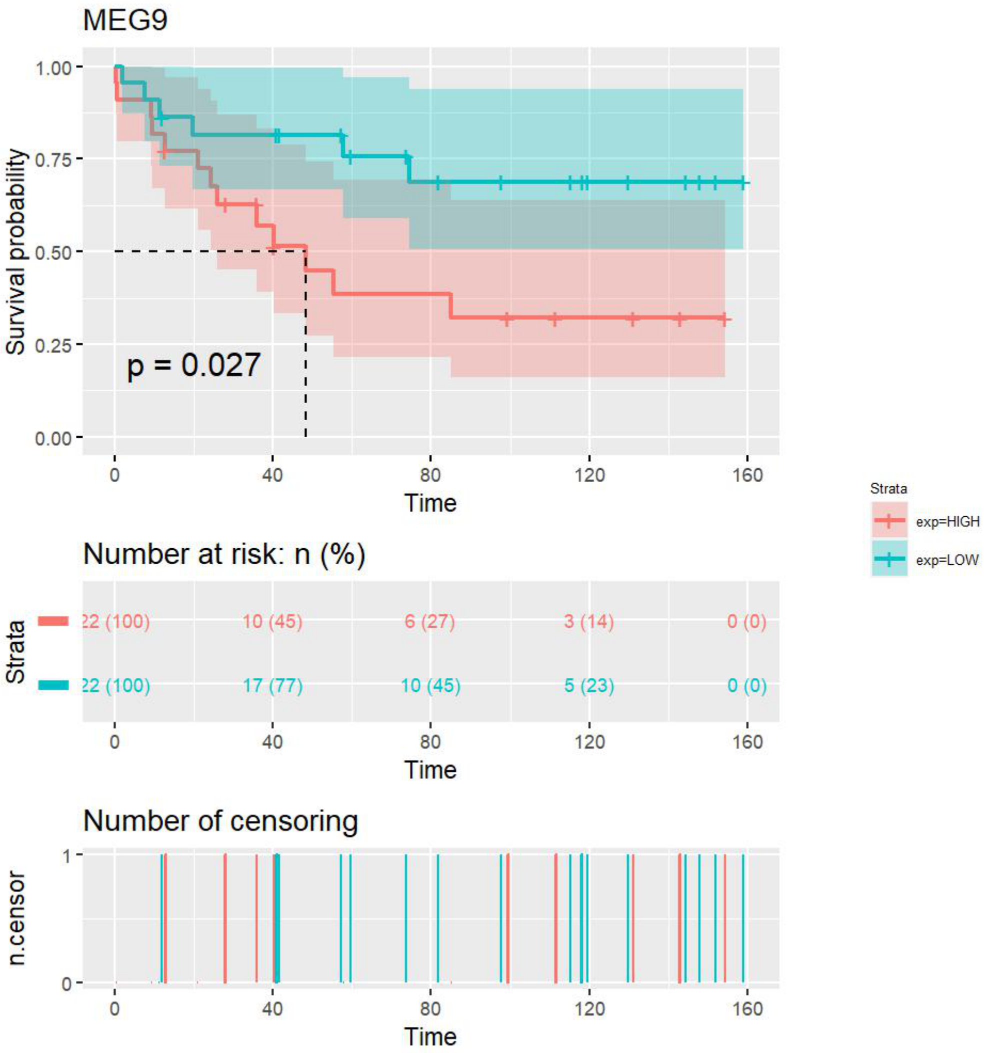

MEG9
Survival probability
1.00
0.75
0.50
0.25
0.00
p = 0.027
0
40
80
120
160
Time
Strata
exp=HIGH
exp=LOW
Number at risk: n (%)
Strata
22 (100)
10 (45)
6 (27)
3 (14)
0 (0)
22 (100)
17 (77)
10 (45)
5 (23)
0 (0)
Number of censoring
n.censor
1
0

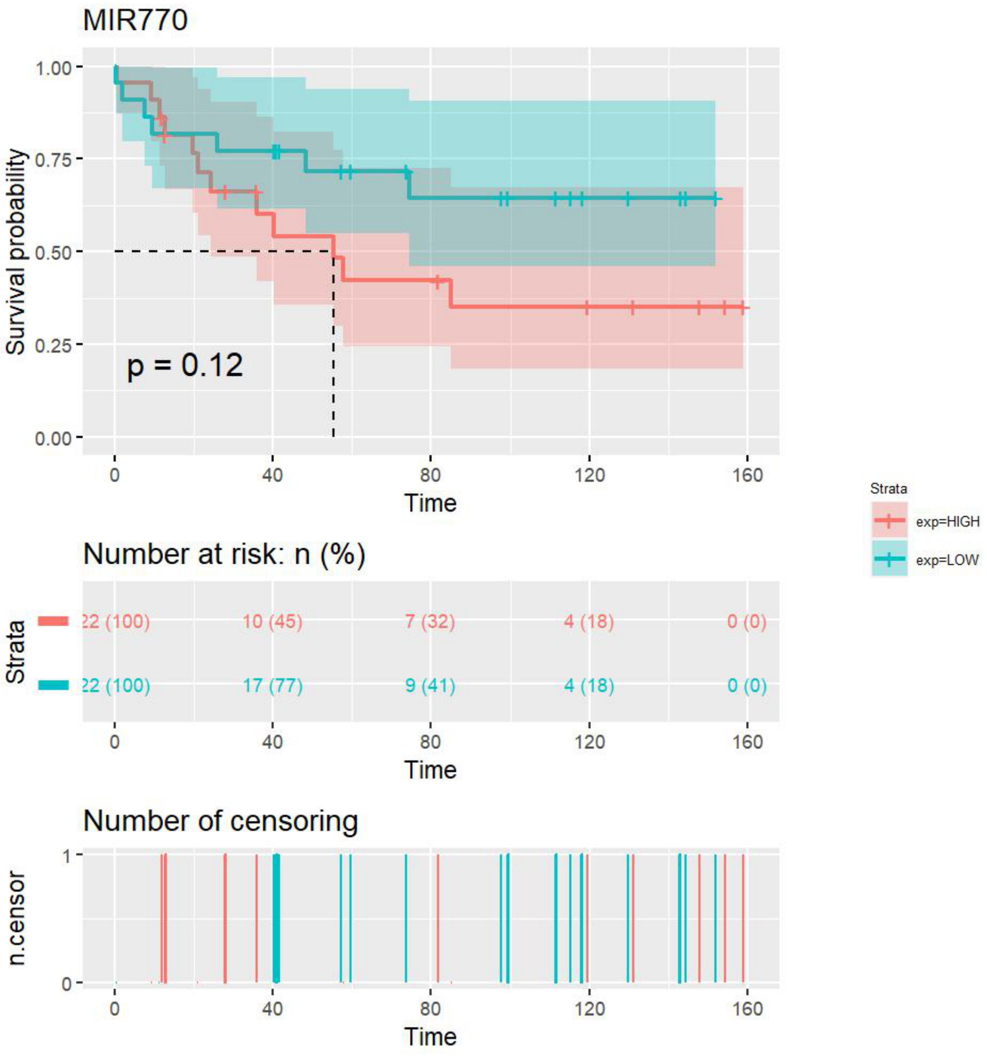
MIR770
Survival probability
1.00
0.75
0.50
0.25
0.00
p = 0.12
0
40
80
120
160
Time
Strata
exp=HIGH
exp=LOW
Number at risk: n (%)
Strata
22 (100)
10 (45)
7 (32)
4 (18)
0 (0)
22 (100)
17 (77)
9 (41)
4 (18)
0 (0)
Number of censoring
n.censor
1
0

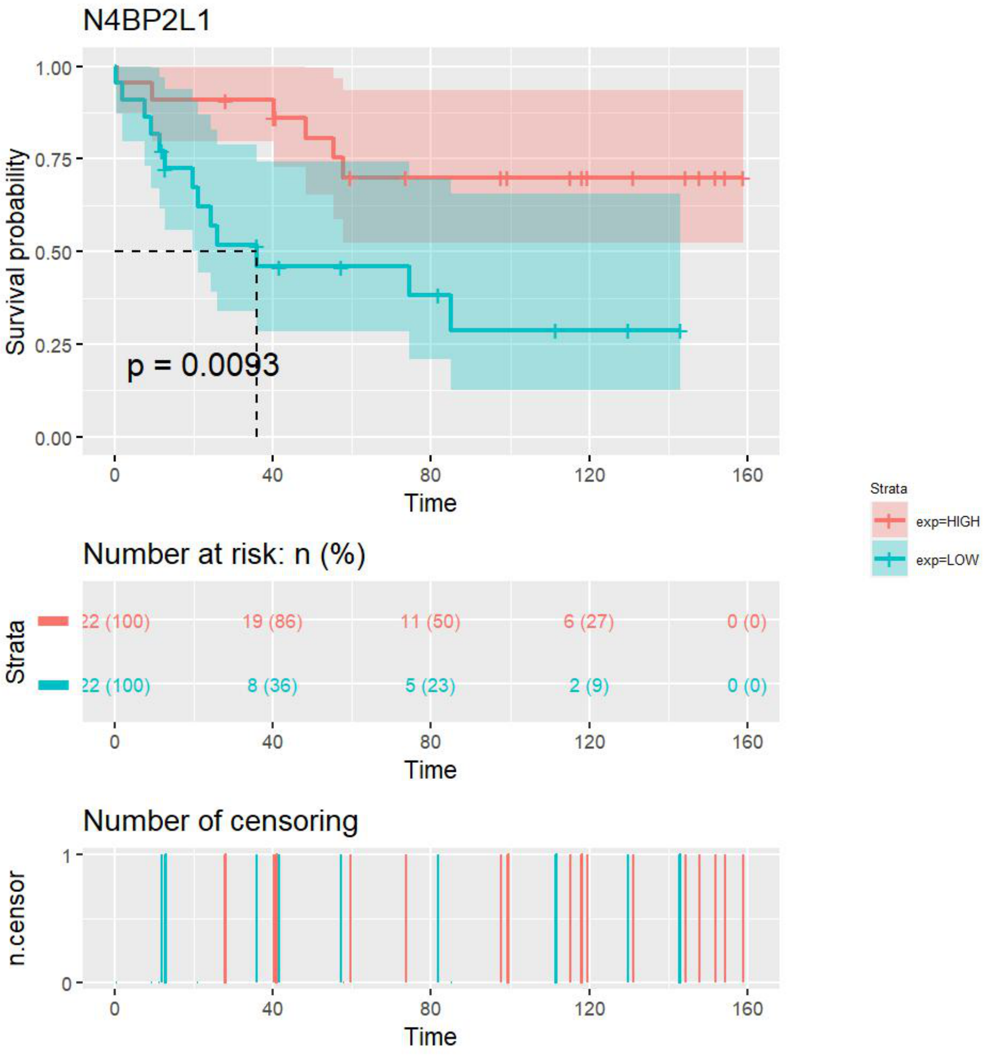
N4BP2L1
Survival probability
1.00
0.75
0.50
0.25
0.00
p = 0.0093
0
40
80
120
160
Time
Strata
exp=HIGH
exp=LOW
Number at risk: n (%)
Strata
22 (100)
19 (86)
11 (50)
6 (27)
0 (0)
22 (100)
8 (36)
5 (23)
2 (9)
0 (0)
0
40
80
120
160
Time
Number of censoring
n.censor
1
0
0
40
80
120
160
Time

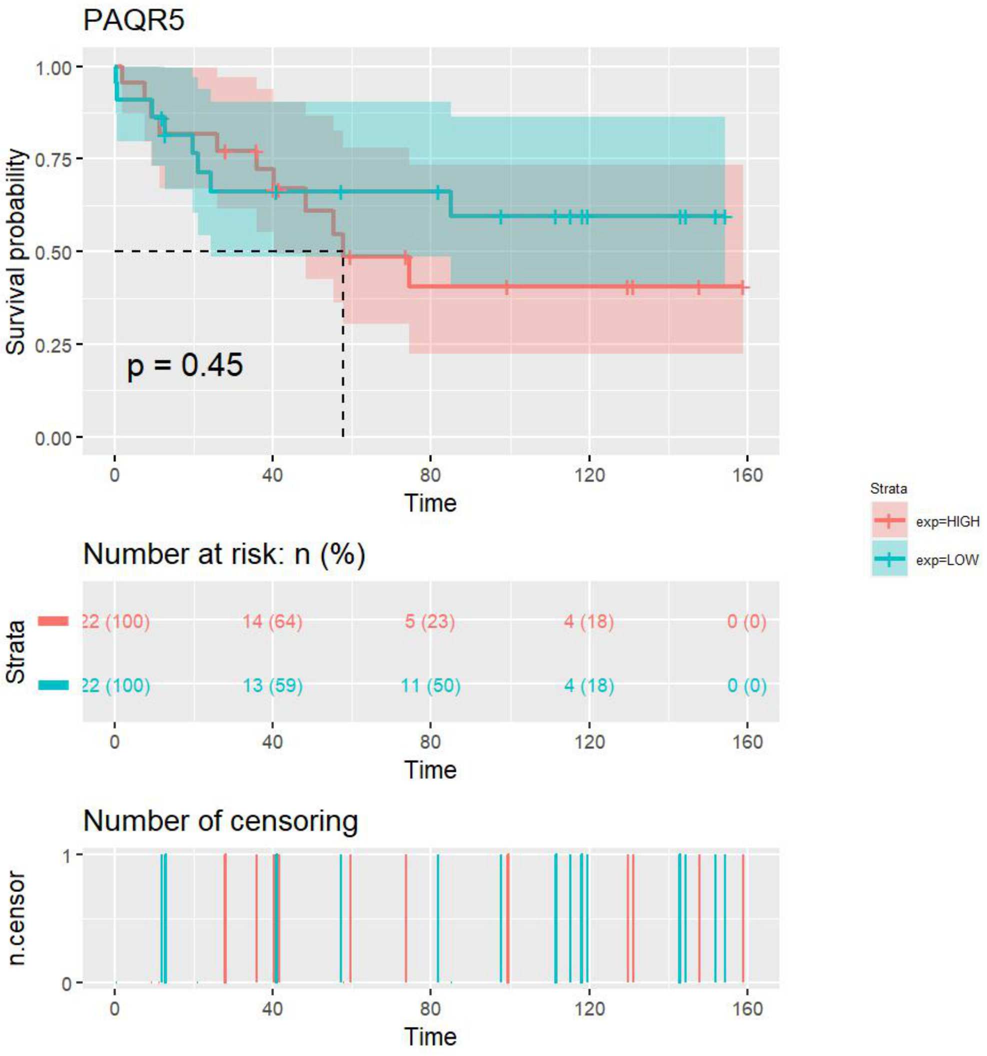
PAQR5
Survival probability
1.00
0.75
0.50
0.25
0.00
p = 0.45
0
40
80
120
160
Time
Strata
exp=HIGH
exp=LOW
Number at risk: n (%)
Strata
22 (100)
14 (64)
5 (23)
4 (18)
0 (0)
22 (100)
13 (59)
11 (50)
4 (18)
0 (0)
Time
Number of censoring
n.censor
1
0
Time

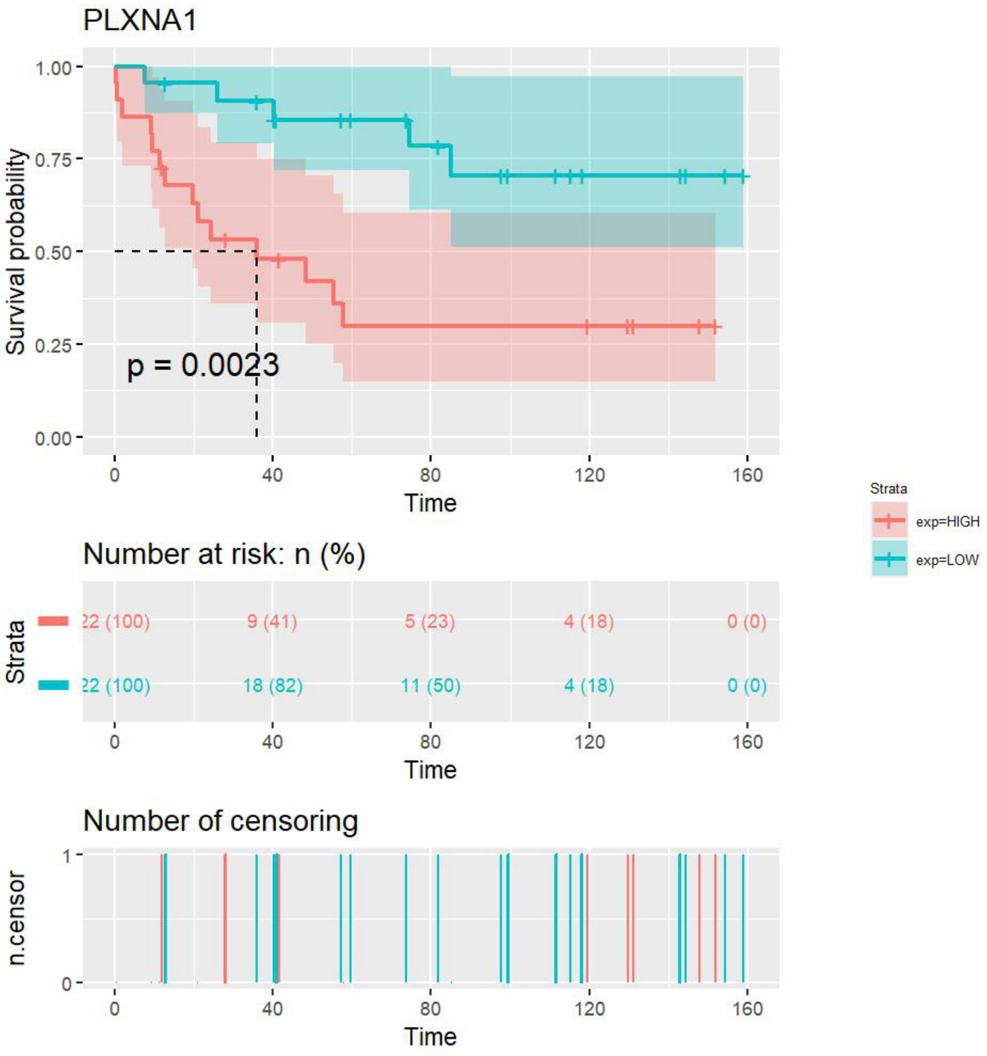
PLXNA1
Survival probability
1.00
0.75
0.50
0.25
0.00
p = 0.0023
0
40
80
120
160
Time
Strata
exp=HIGH
exp=LOW
Number at risk: n (%)
Strata
22 (100)
9 (41)
5 (23)
4 (18)
0 (0)
22 (100)
18 (82)
11 (50)
4 (18)
0 (0)
Time
Number of censoring
n.censor
1
0
Time

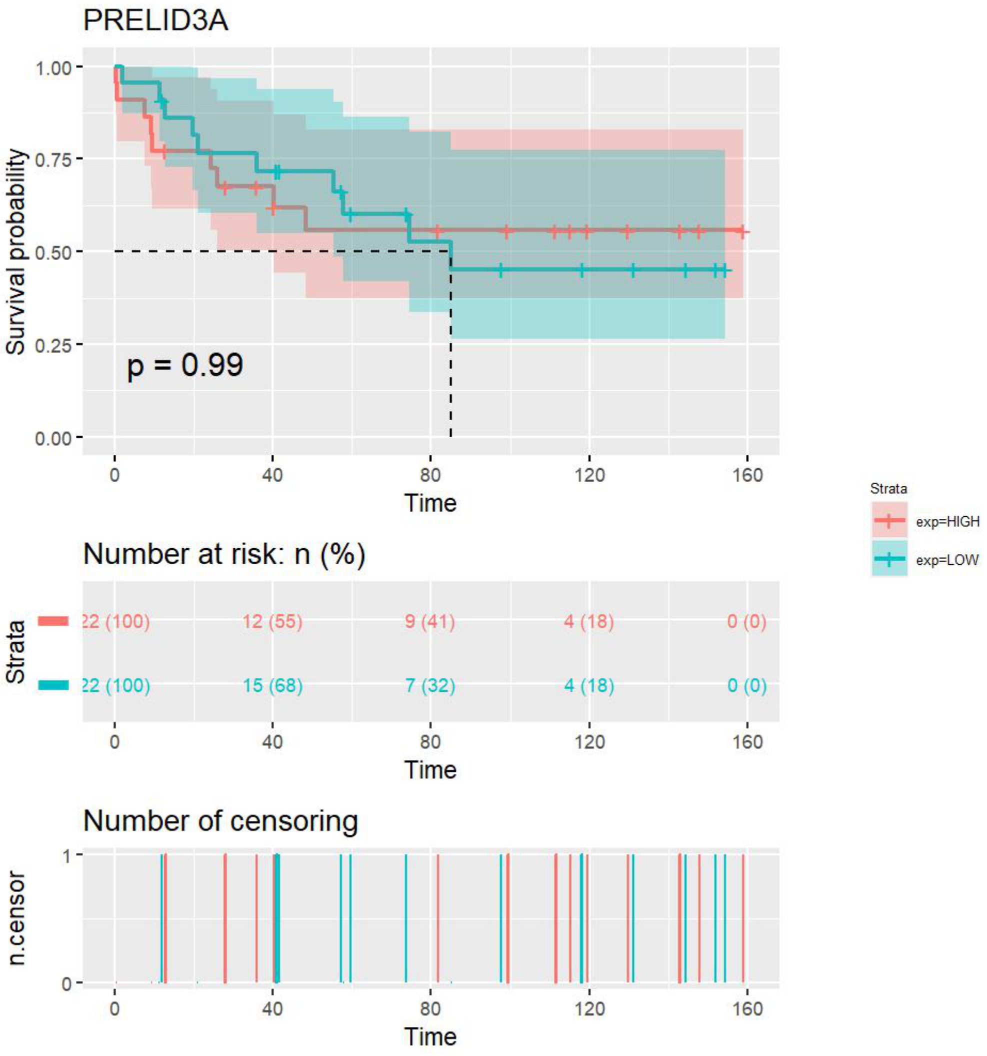

PRELID3A
Survival probability
1.00
0.75
0.50
0.25
0.00
p = 0.99
0
40
80
120
160
Time
Strata
exp=HIGH
exp=LOW
Number at risk: n (%)
22 (100)
12 (55)
9 (41)
4 (18)
0 (0)
22 (100)
15 (68)
7 (32)
4 (18)
0 (0)
Number of censoring
n.censor
1
0

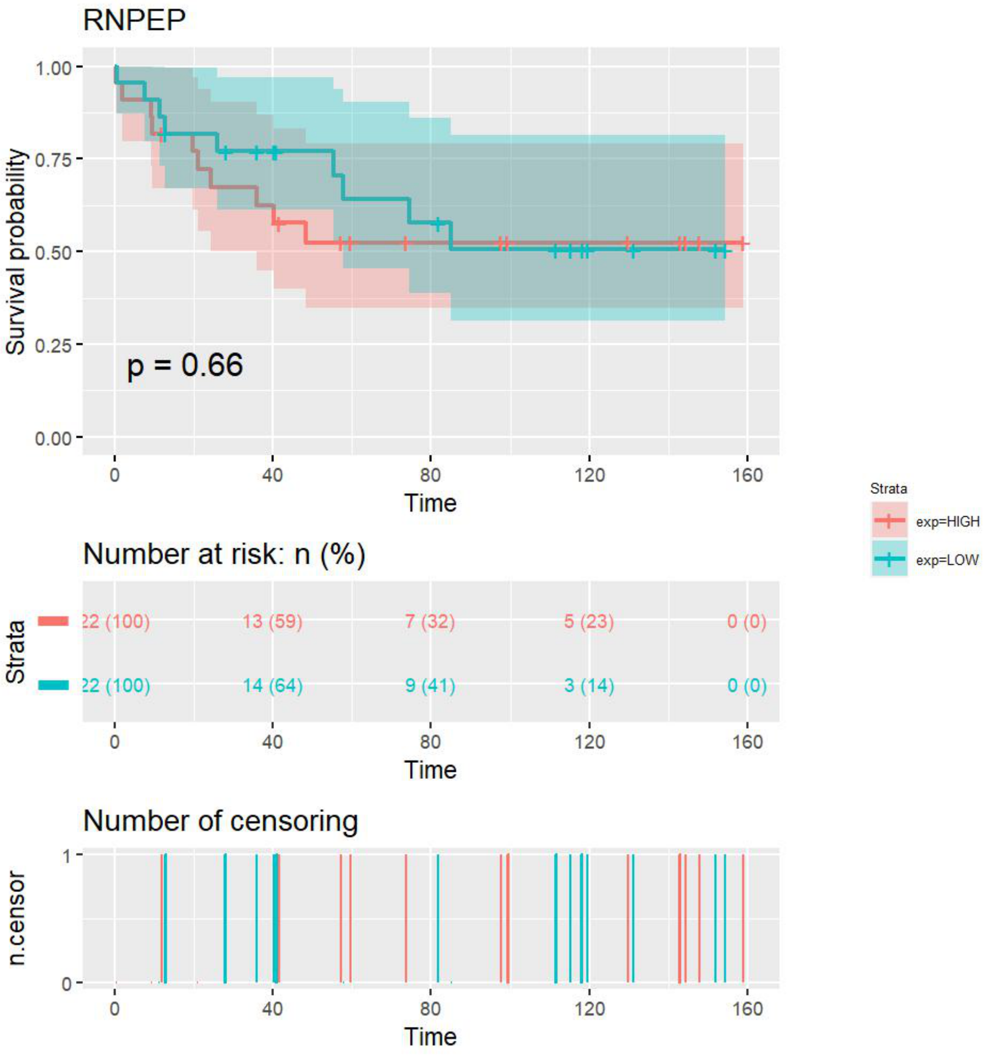
RNPEP
Survival probability
1.00
0.75
0.50
0.25
0.00
p = 0.66
0
40
80
120
160
Time
Strata
exp=HIGH
exp=LOW
Number at risk: n (%)
Strata
22 (100)
13 (59)
7 (32)
5 (23)
0 (0)
22 (100)
14 (64)
9 (41)
3 (14)
0 (0)
Number of censoring
n.censor
1
0

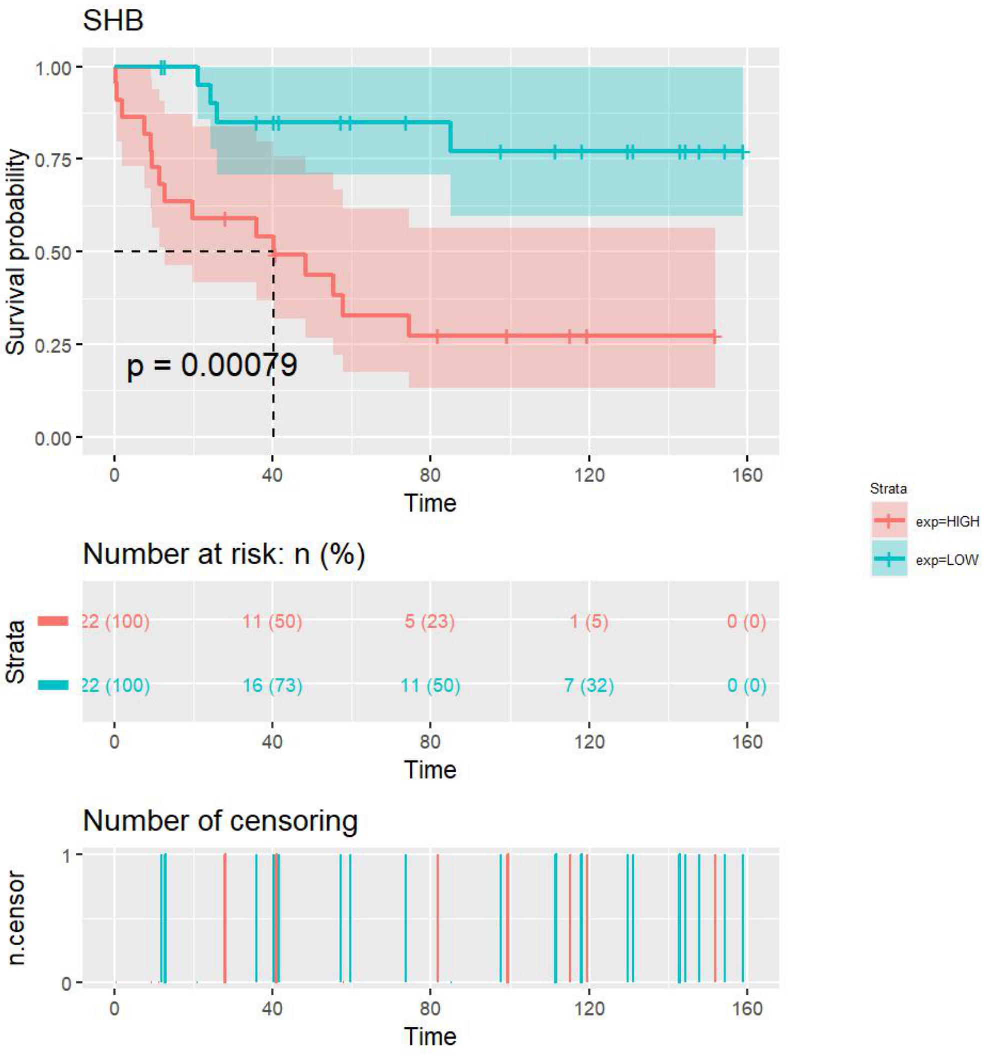

SHB
Survival probability
1.00
0.75
0.50
0.25
0.00
p = 0.00079
0
40
80
120
160
Time
Strata
exp=HIGH
exp=LOW
Number at risk: n (%)
Strata
22 (100)
11 (50)
5 (23)
1 (5)
0 (0)
22 (100)
16 (73)
11 (50)
7 (32)
0 (0)
Time
Number of censoring
n.censor
1
0
Time

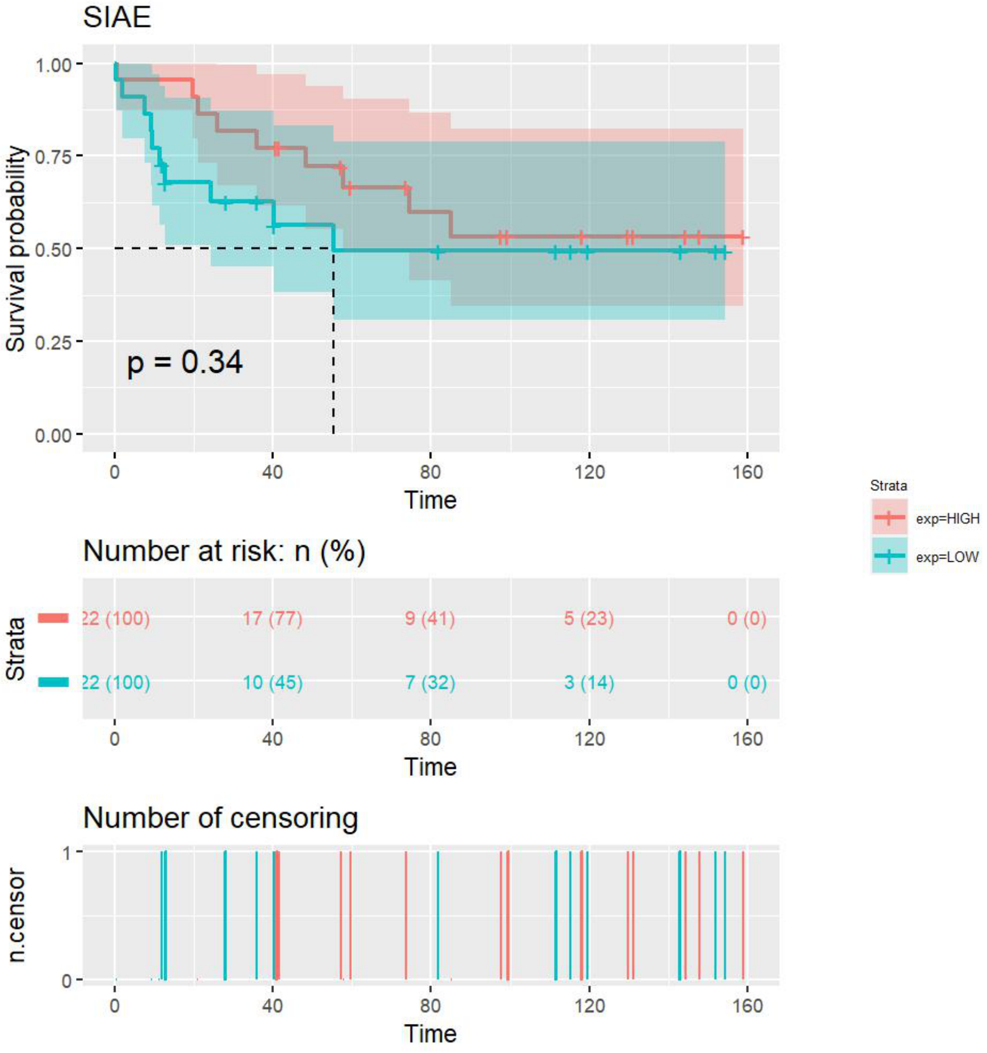

SIAE
Survival probability
1.00
0.75
0.50
0.25
0.00
p = 0.34
0
40
80
120
160
Time
Strata
exp=HIGH
exp=LOW
Number at risk: n (%)
Strata
22 (100)
17 (77)
9 (41)
5 (23)
0 (0)
22 (100)
10 (45)
7 (32)
3 (14)
0 (0)
Number of censoring
n.censor
1
0

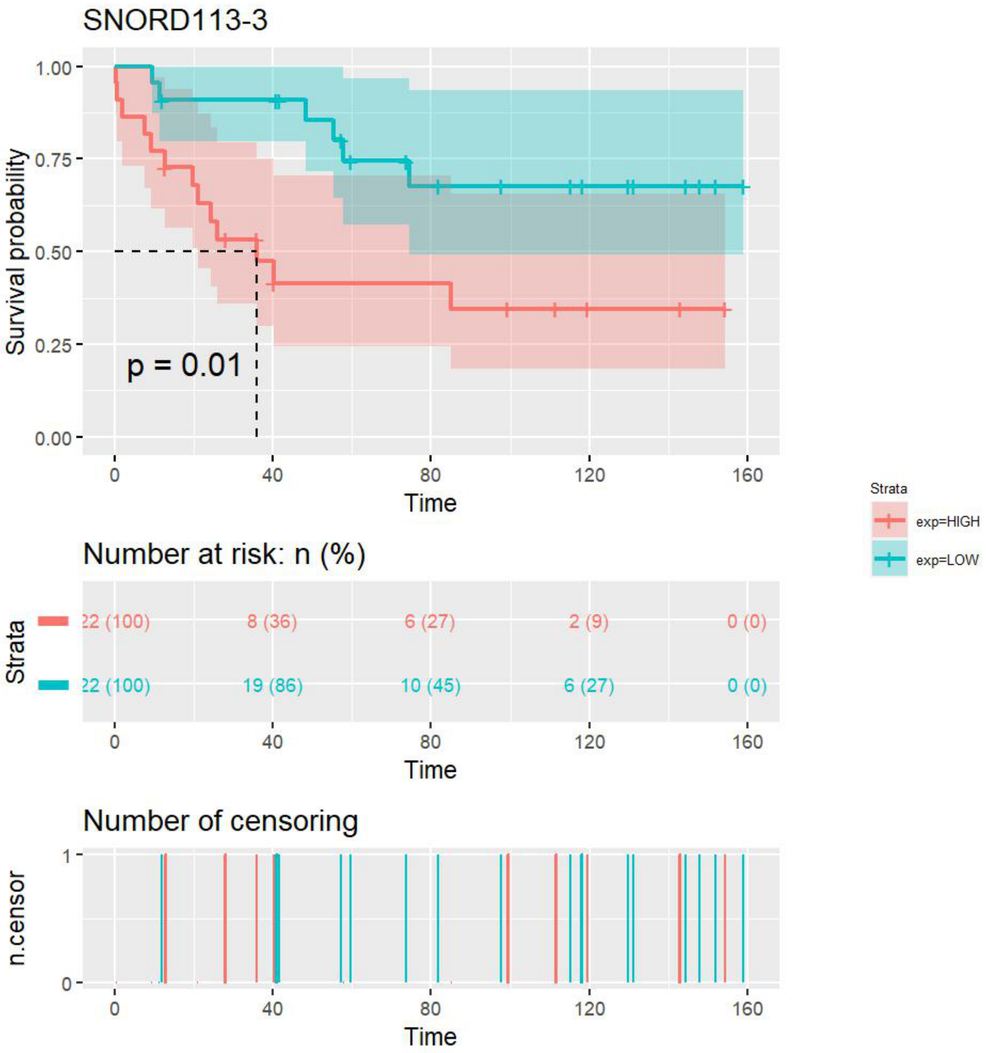
SNORD113-3
Survival probability
1.00
0.75
0.50
0.25
0.00
p = 0.01
0
40
80
120
160
Time
Strata
exp=HIGH
exp=LOW
Number at risk: n (%)
Strata
22 (100)
8 (36)
6 (27)
2 (9)
0 (0)
22 (100)
19 (86)
10 (45)
6 (27)
0 (0)
Time
Number of censoring
n.censor
1
0
Time

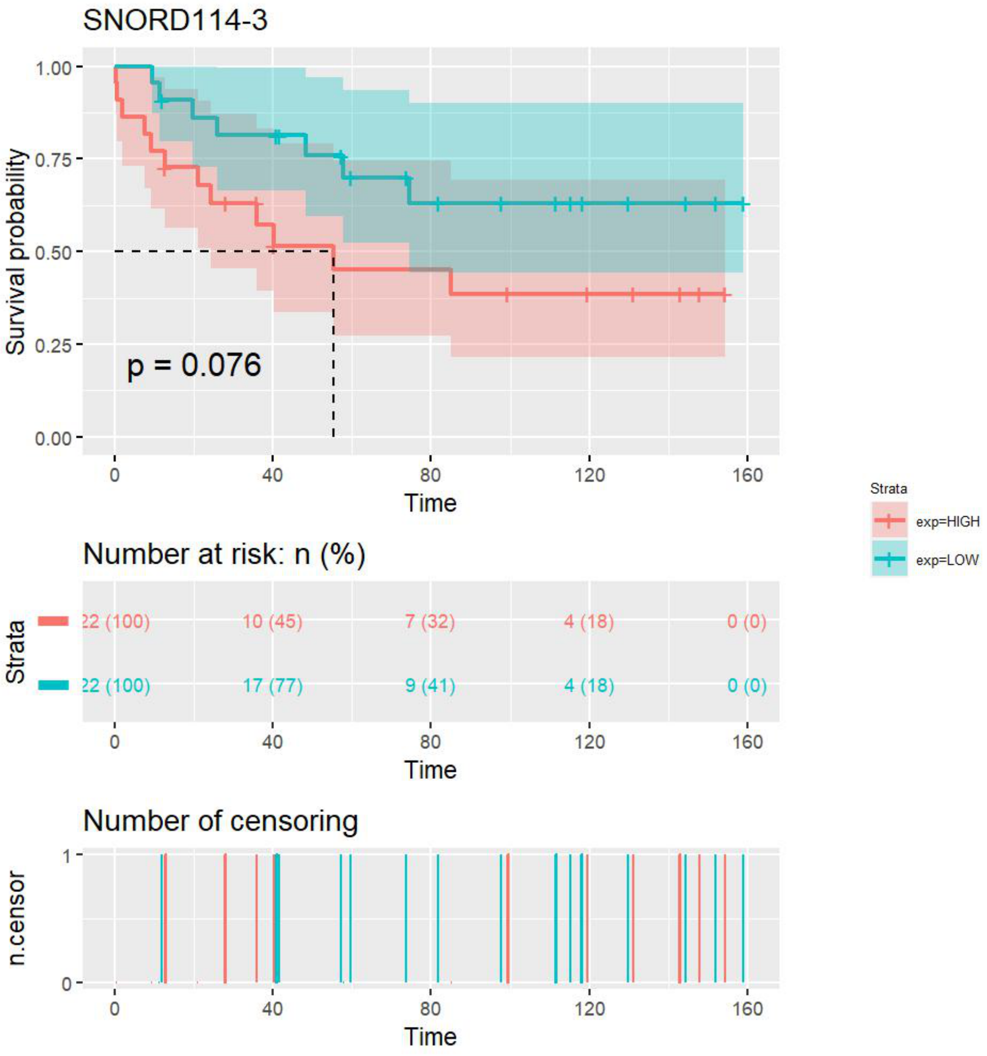

SNORD114-3
Survival probability
1.00
0.75
0.50
0.25
0.00
p = 0.076
0
40
80
120
160
Time
Strata
exp=HIGH
exp=LOW
Number at risk: n (%)
Strata
22 (100)
10 (45)
7 (32)
4 (18)
0 (0)
22 (100)
17 (77)
9 (41)
4 (18)
0 (0)
Time
Number of censoring
n.censor
1
0
Time

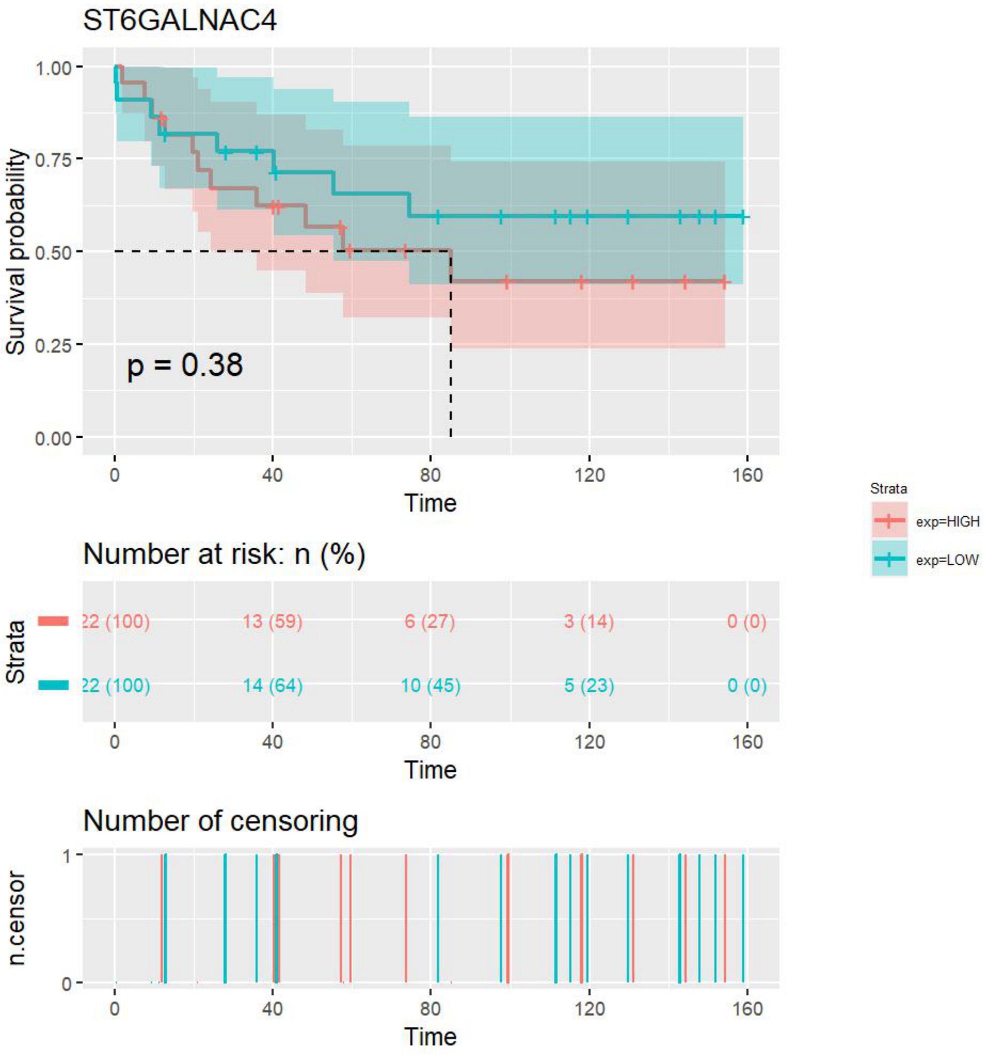
ST6GALNAC4
Survival probability
1.00
0.75
0.50
0.25
0.00
p = 0.38
0
40
80
120
160
Time
Strata
exp=HIGH
exp=LOW
Number at risk: n (%)
Strata
22 (100) 13 (59) 6 (27) 3 (14) 0 (0)
22 (100) 14 (64) 10 (45) 5 (23) 0 (0)
Time
Number of censoring
n.censor
1
0
Time

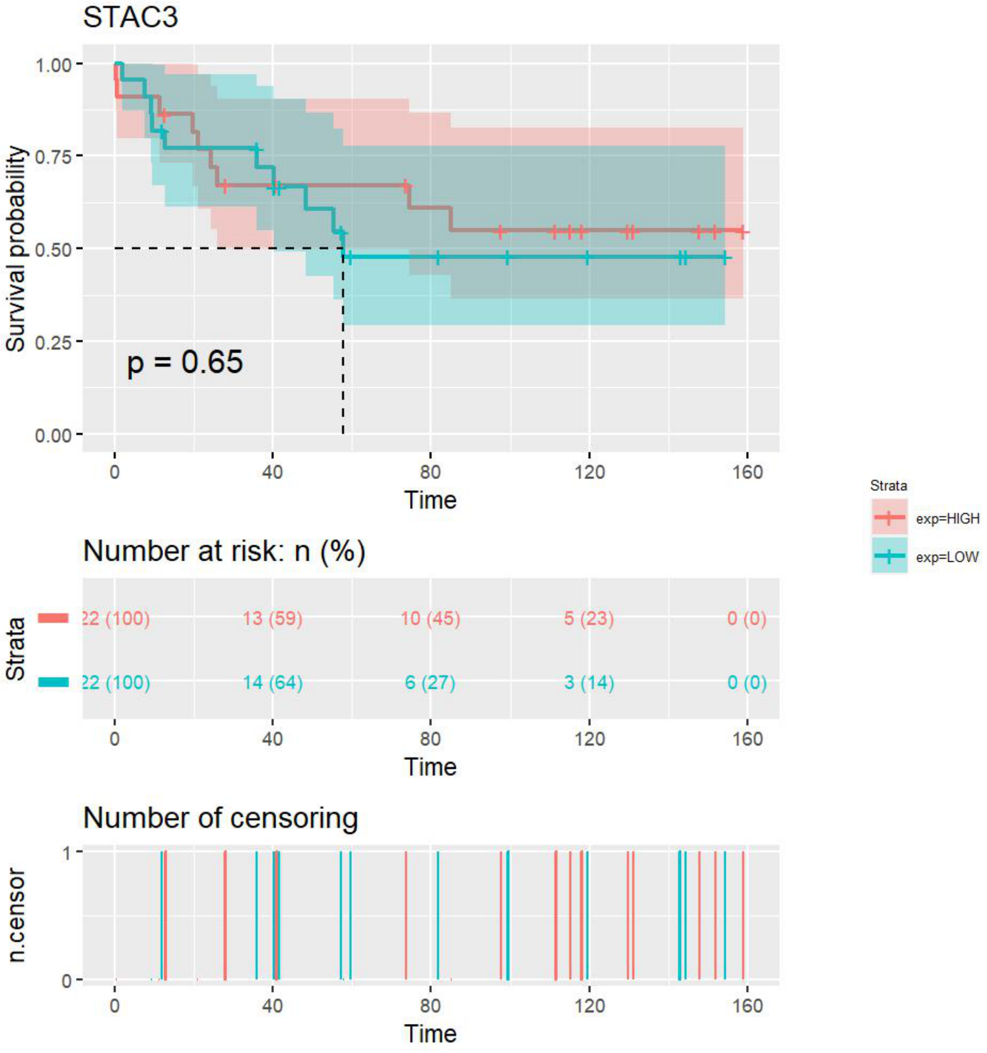

STAC3
Survival probability
1.00
0.75
0.50
0.25
0.00
p = 0.65
0
40
80
120
160
Time
Strata
exp=HIGH
exp=LOW
Number at risk: n (%)
Strata
22 (100)
13 (59)
10 (45)
5 (23)
0 (0)
22 (100)
14 (64)
6 (27)
3 (14)
0 (0)
Time
Number of censoring
n.censor
1
0
Time

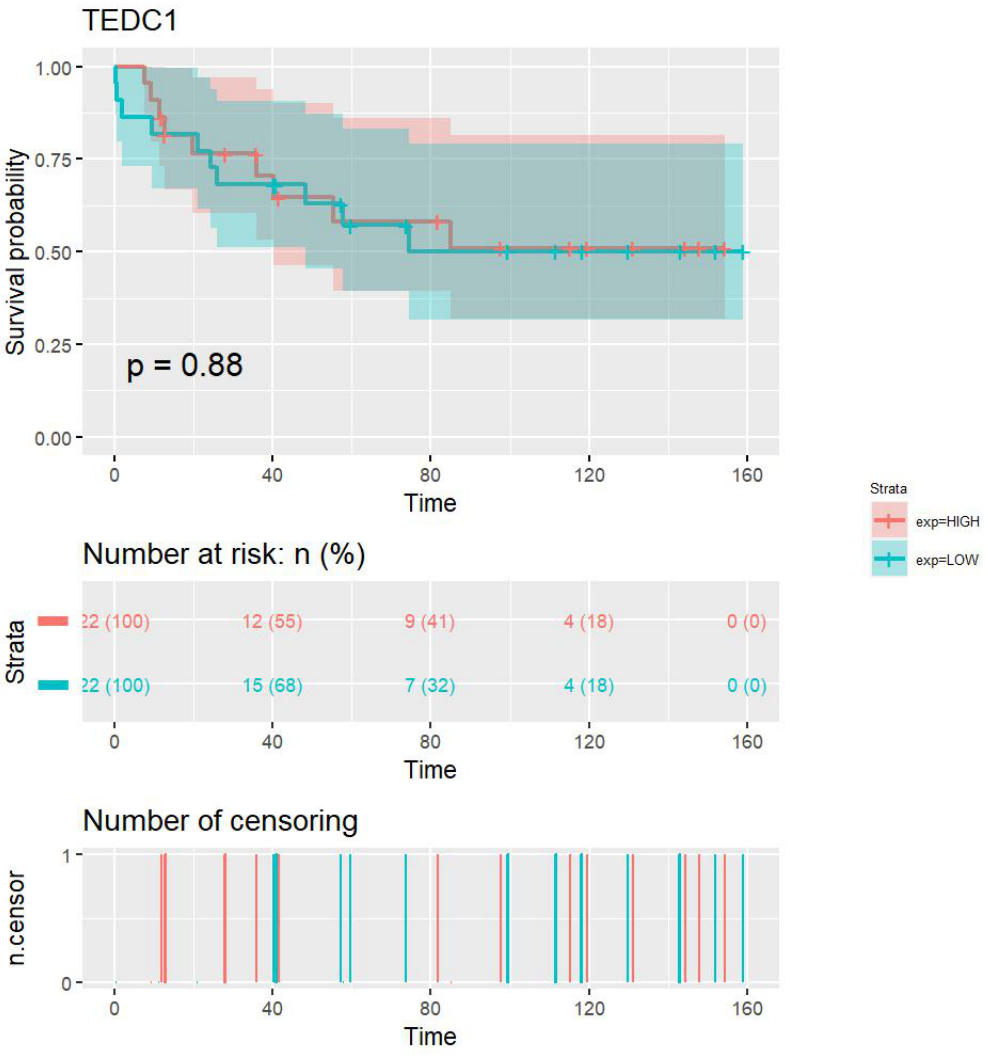
TEDC1
Survival probability
1.00
0.75
0.50
0.25
0.00
p = 0.88
0
40
80
120
160
Time
Strata
exp=HIGH
exp=LOW
Number at risk: n (%)
22 (100)
12 (55)
9 (41)
4 (18)
0 (0)
22 (100)
15 (68)
7 (32)
4 (18)
0 (0)
Number of censoring
n.censor
1
0

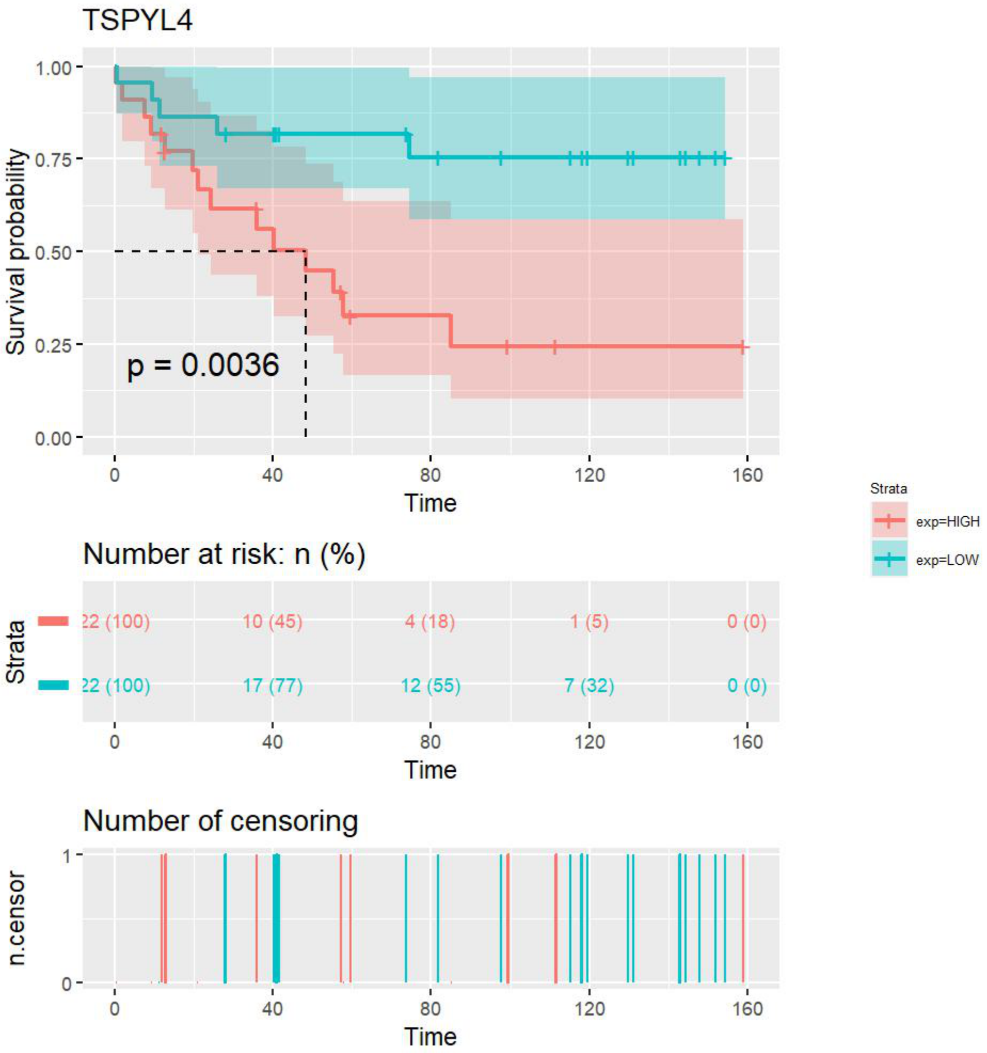
TSPYL4
Survival probability
1.00
0.75
0.50
0.25
0.00
p = 0.0036
0
40
80
120
160
Time
Strata
exp=HIGH
exp=LOW
Number at risk: n (%)
Strata
22 (100)
10 (45)
4 (18)
1 (5)
0 (0)
22 (100)
17 (77)
12 (55)
7 (32)
0 (0)
Time
Number of censoring
n.censor
1
0
Time

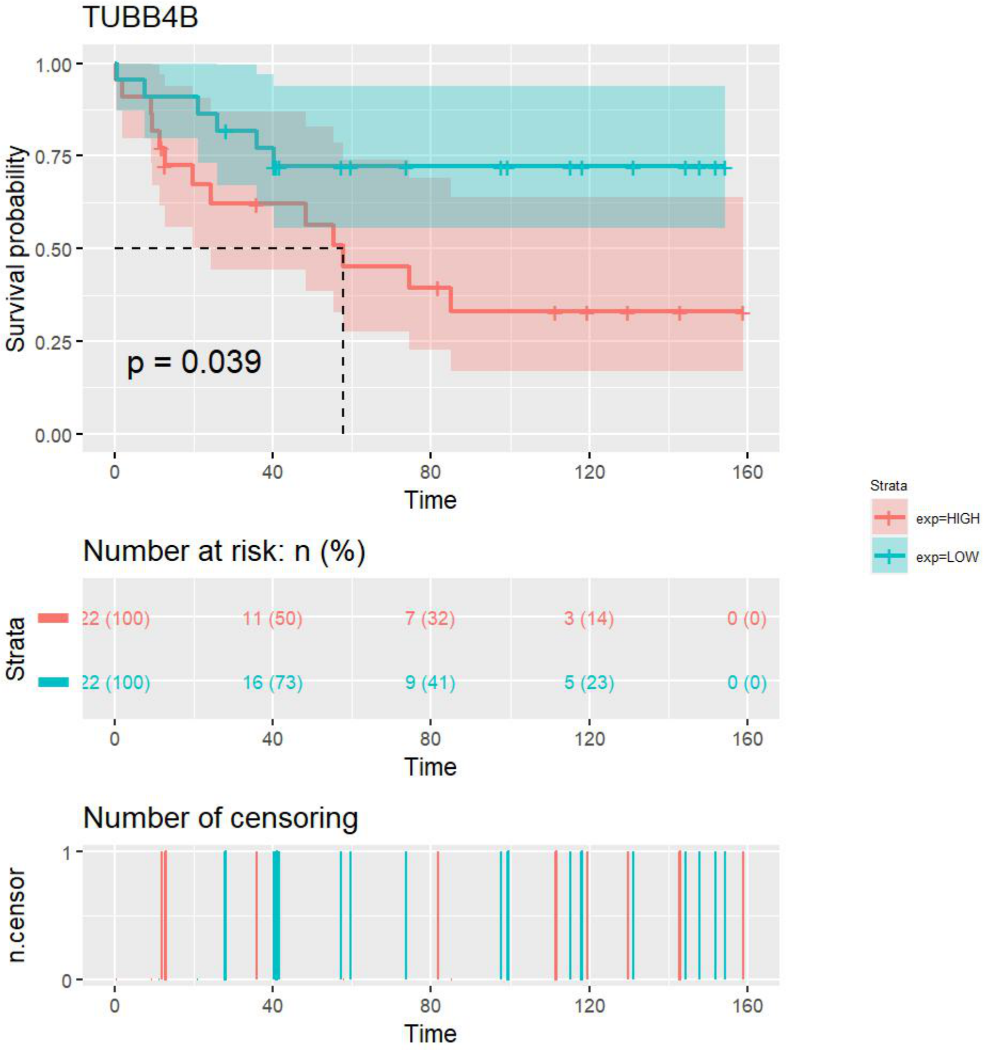
TUBB4B
Survival probability
1.00
0.75
0.50
0.25
0.00
p = 0.039
0
40
80
120
160
Time
Strata
exp=HIGH
exp=LOW
Number at risk: n (%)
Strata
22 (100)
11 (50)
7 (32)
3 (14)
0 (0)
22 (100)
16 (73)
9 (41)
5 (23)
0 (0)
Number of censoring
n.censor
1
0

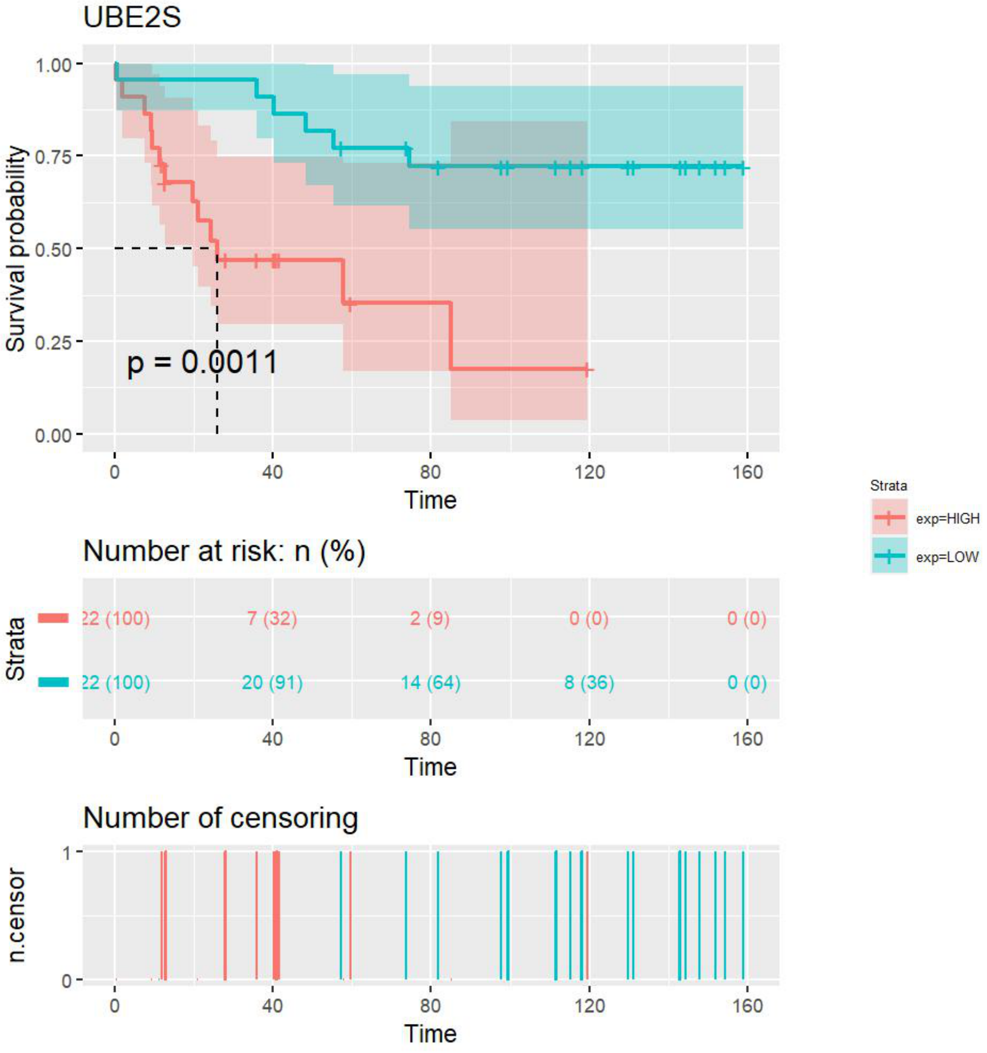
UBE2S
Survival probability
1.00
0.75
0.50
0.25
0.00
p = 0.0011
0
40
80
120
160
Time
Strata
exp=HIGH
exp=LOW
Number at risk: n (%)
Strata
22 (100)
7 (32)
2 (9)
0 (0)
0 (0)
22 (100)
20 (91)
14 (64)
8 (36)
0 (0)
Time
Number of censoring
n.censor
1
0
Time

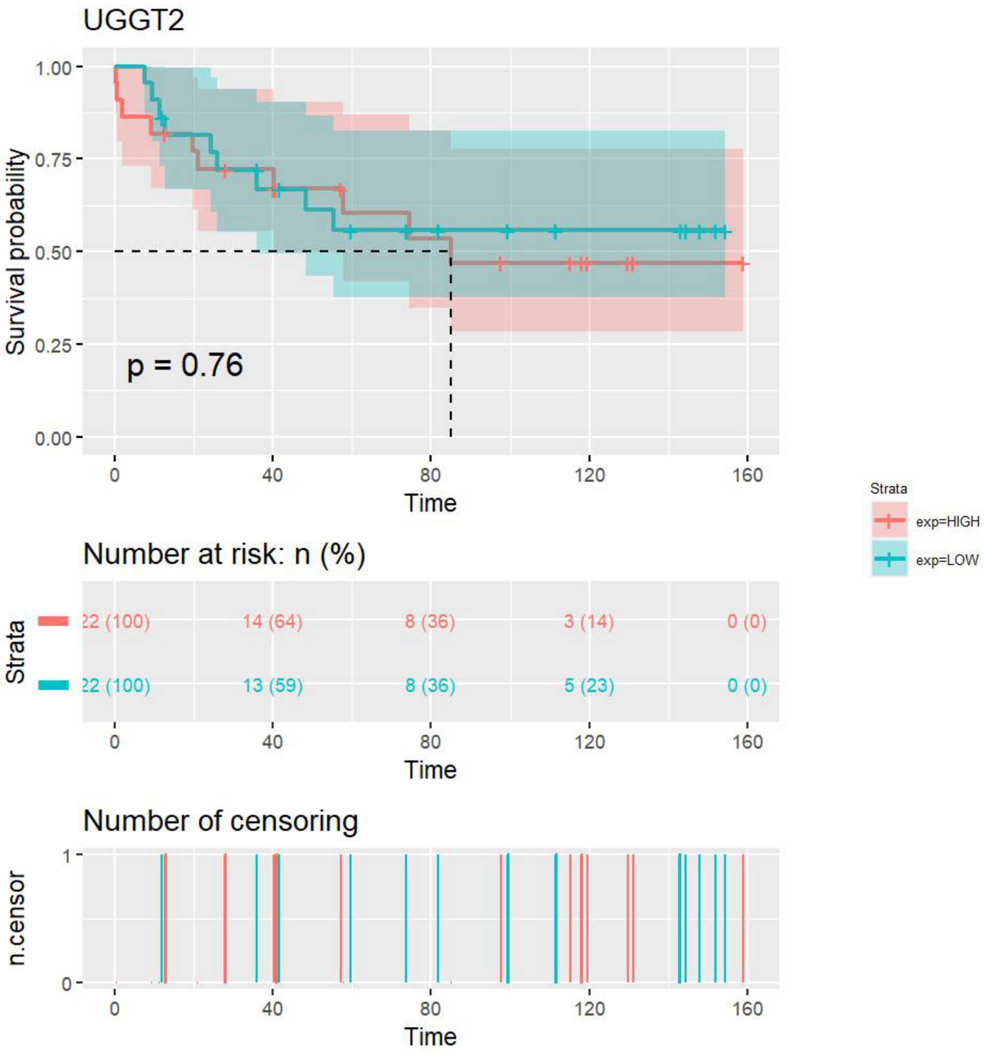

UGGT2
Survival probability
1.00
0.75
0.50
0.25
0.00
p = 0.76
0
40
80
120
160
Time
Strata
exp=HIGH
exp=LOW
Number at risk: n (%)
Strata
22 (100)
14 (64)
8 (36)
3 (14)
0 (0)
22 (100)
13 (59)
8 (36)
5 (23)
0 (0)
Time
Number of censoring
n.censor
1
0
Time

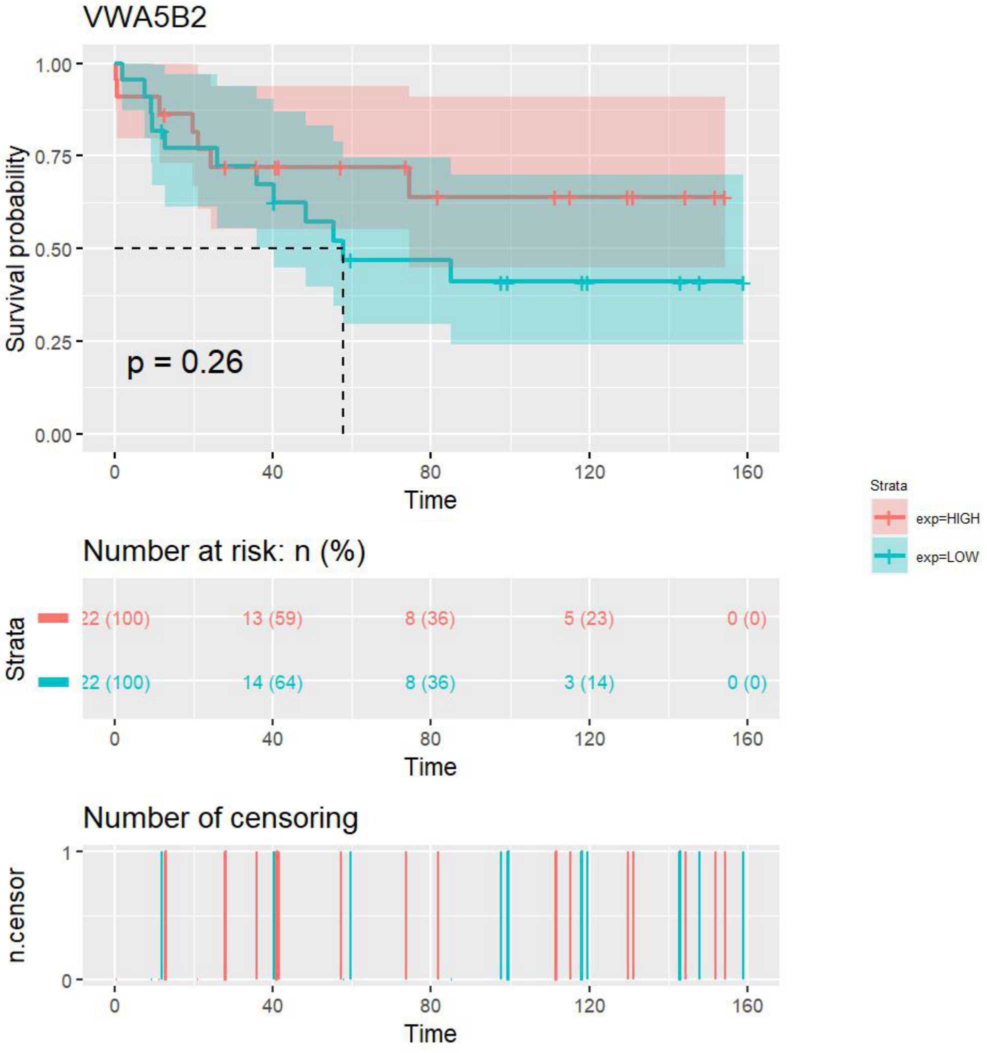
VWA5B2
Survival probability
1.00
0.75
0.50
0.25
0.00
p = 0.26
0
40
80
120
160
Time
Strata
exp=HIGH
exp=LOW
Number at risk: n (%)
Strata
22 (100)
13 (59)
8 (36)
5 (23)
0 (0)
22 (100)
14 (64)
8 (36)
3 (14)
0 (0)
Time
Number of censoring
n.censor
1
0
Time

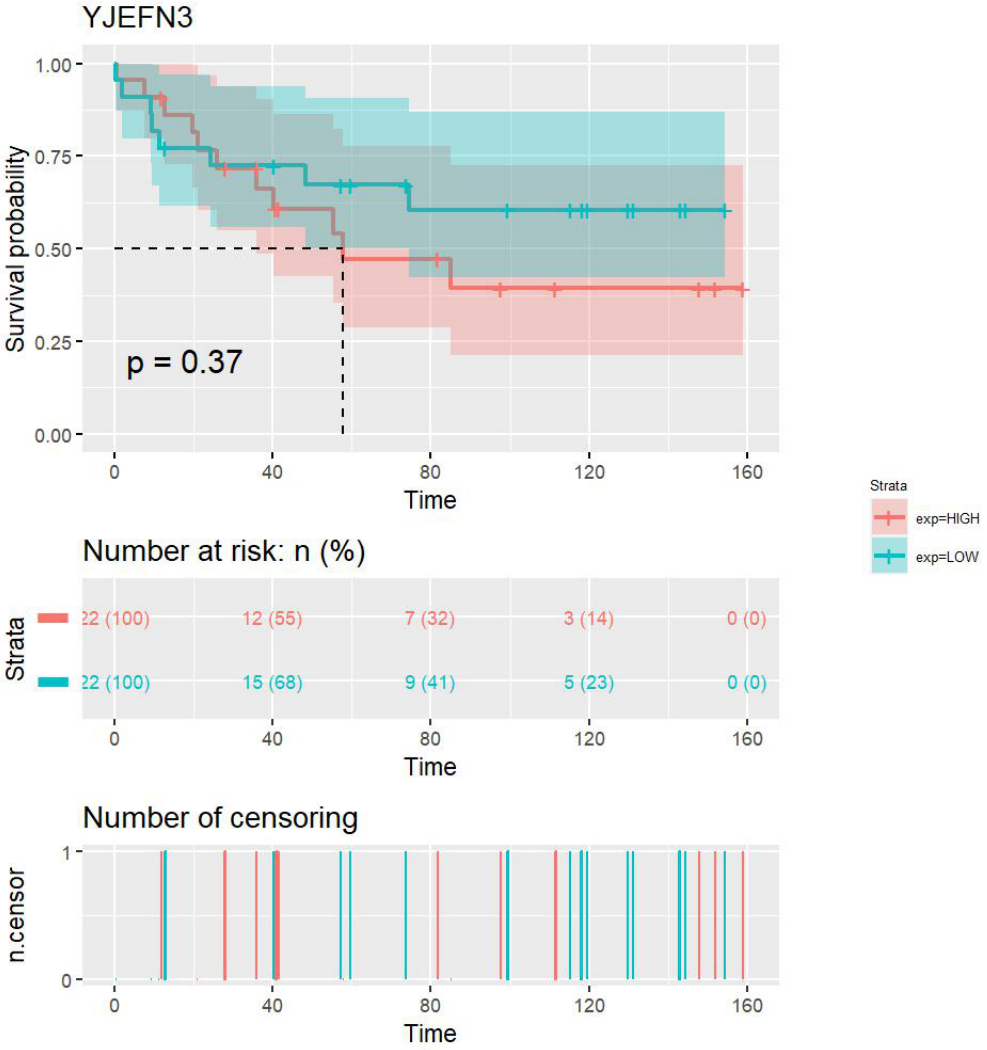

YJEFN3
Survival probability
1.00
0.75
0.50
0.25
0.00
p = 0.37
0
40
80
120
160
Time
Strata
exp=HIGH
exp=LOW
Number at risk: n (%)
Strata
22 (100)
12 (55)
7 (32)
3 (14)
0 (0)
22 (100)
15 (68)
9 (41)
5 (23)
0 (0)
Time
Number of censoring
n.censor
1
0
Time

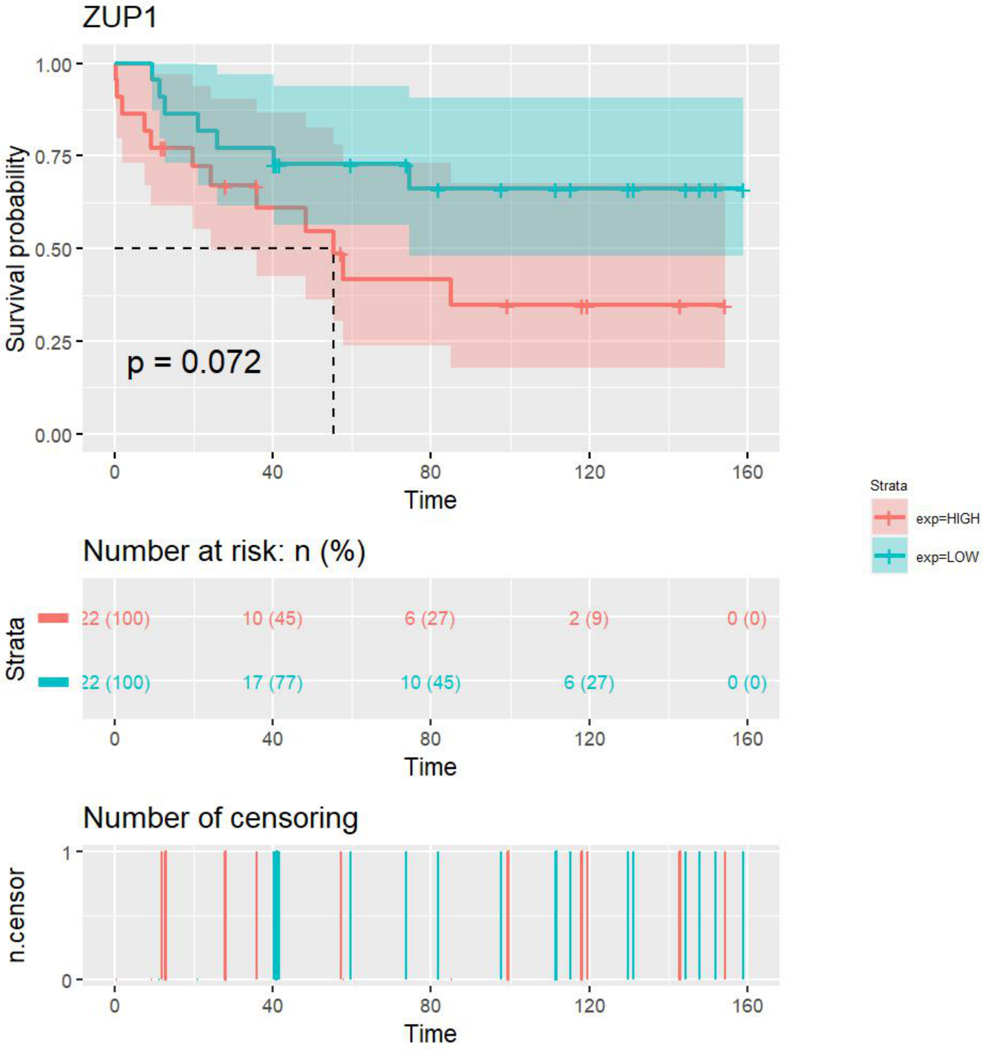
ZUP1
Survival probability
1.00
0.75
0.50
0.25
0.00
p = 0.072
0
40
80
120
160
Time
Strata
exp=HIGH
exp=LOW
Number at risk: n (%)
22 (100)
10 (45)
6 (27)
2 (9)
0 (0)
22 (100)
17 (77)
10 (45)
6 (27)
0 (0)
Number of censoring
n.censor
1
0
